# Expanding the Solid Form Landscape of Bipyridines

**DOI:** 10.1021/acs.cgd.1c01045

**Published:** 2021-11-10

**Authors:** Doris E. Braun, Patricia Hald, Volker Kahlenberg, Ulrich J. Griesser

**Affiliations:** † Institute of Pharmacy, University of Innsbruck, Innrain 52c, 6020 Innsbruck, Austria; ‡ Institute of Mineralogy and Petrography, University of Innsbruck, Innrain 52, 6020 Innsbruck, Austria

## Abstract

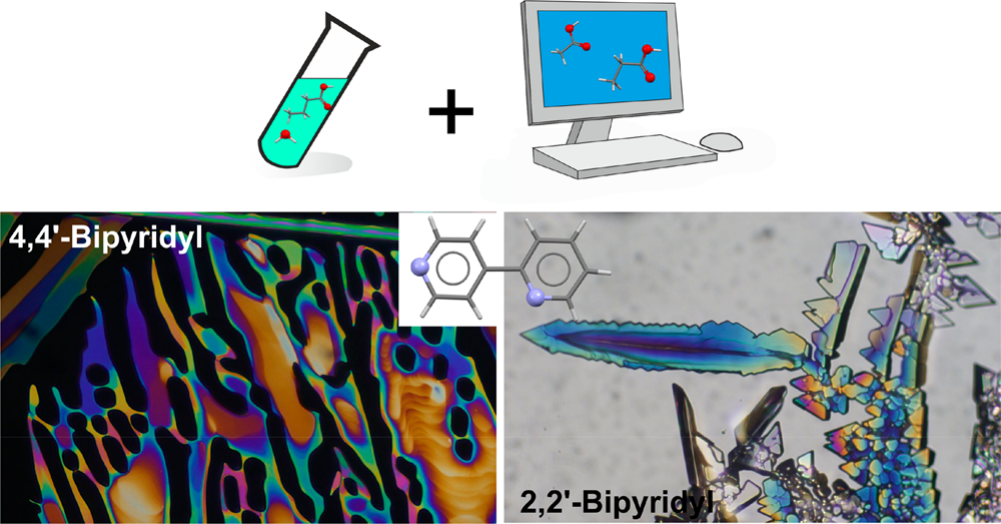

Two bipyridine isomers (2,2′- and 4,4′-), used as coformers and ligands in coordination chemistry, were subjected to solid form screening and crystal structure prediction. One anhydrate and a formic acid disolvate were crystallized for 2,2′-bipyridine, whereas multiple solid-state forms, anhydrate, dihydrate, and eight solvates with carboxylic acids, including a polymorphic acetic acid disolvate, were found for the 4,4′-isomer. Seven of the solvates are reported for the first time, and structural information is provided for six of the new solvates. All twelve solid-state forms were investigated comprehensively using experimental [thermal analysis, isothermal calorimetry, X-ray diffraction, gravimetric moisture (de)sorption, and IR spectroscopy] and computational approaches. Lattice and interaction energy calculations confirmed the thermodynamic driving force for disolvate formation, mediated by the absence of H-bond donor groups of the host molecules. The exposed location of the N atoms in 4,4′-bipyridine facilitates the accommodation of bigger carboxylic acids and leads to higher conformational flexibility compared to 2,2′-bipyridine. For the 4,4′-bipyridine anhydrate ↔ hydrate interconversion hardly any hysteresis and a fast transformation kinetics are observed, with the critical relative humidity being at 35% at room temperature. The computed anhydrate crystal energy landscapes have the 2,2′-bipyridine as the lowest energy structure and the 4,4′-bipyridine among the low-energy structures and suggest a different crystallization behavior of the two compounds.

## Abstract 2

The solid form landscapes of 2,2′- and 4,4′-bipyridine were explored experimentally and computationally. The position of the nitrogen atoms defines not only the conformational flexibility of the molecule but also the crystallization behavior. 4.4′-Bipyridine was found to be more prone to solvate (including hydrate) formation than its 2,2′-isomer.

## 1 Introduction

It is well-known that a given organic compound can exist in more than one solid-state form. These various solid forms, polymorphs, solvates, hydrates, or amorphous forms normally show different physicochemical properties and may coexist under some pressure and temperature conditions.^1−3^ Control of the solid-state form is required to ensure the optimal performance of a product. As an integral part of the drug development process, every compound is normally subjected to extensive crystal form screening in order to discover as many polymorphs and solvates as possible.^4−6^ Solid-form properties, such as stability, solubility, dissolution rate, and processability, may be optimized through considerable single- or multicomponent crystals and amorphous forms.^7,8^ The biggest problem with experimental solid-form screening is that scientists must still rely on trial-and-error experimentation with no well-defined end point.^9^ With the goal of an experimental polymorph screening being to find all practically relevant forms, employing crystal structure prediction (CSP) to calculate all thermodynamically feasible crystal structures from a diagram of the molecule could provide much-needed assurance that important forms have been found.^9,10^

Solvent molecules can be included in the crystal structure of a compound, leading to hydrates when the incorporated solvent is water or solvates for any other solvent. The possible interaction with water during certain processing steps (*e.g*., crystallization, wet granulation, freeze–drying), or simply exposure to atmospheric humidity during production or storage, renders hydrate formation extremely common among drug molecules.^11−13^ The tendency for solvates (hydrates) to crystallize more easily than solvent-free forms can be related to the following features: (i) unsatisfied intermolecular interactions, created by an imbalance between hydrogen-bonding donor and hydrogen-bonding acceptor groups in the parent molecule (*e.g*., codeine^14^), and (ii) more efficient packing (“bumps fit into hollows”^15^) as inclusion of the generally smaller solvent decreases the void space in the crystal. Most solvates include contributions from both driving forces.

**Figure 1:**
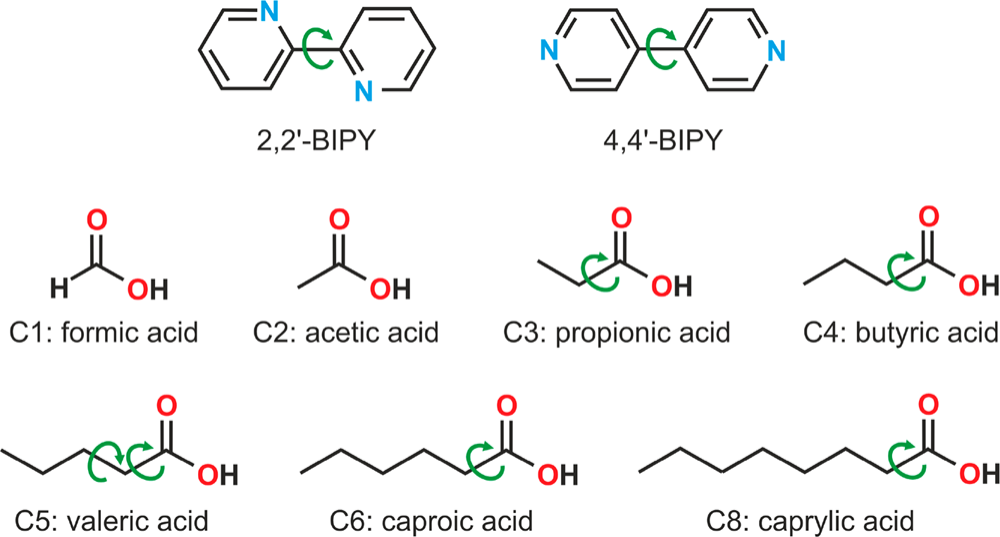
Molecular structures of 2,2′- and 4,4′-bipyridine (BIPY) and solvate forming solvent molecules. Dihedral angles, which were analyzed in more detail, are marked with green arrows.

The importance of bipyridines (Figure 1) as ligands in coordination chemistry has been reviewed.^16,17^ The 2,2′-BIPY ligand is extensively used as a metal chelating ligand due to its robust redox stability and ease of functionalization. 2,2′-BIPY forms charged complexes with metal cations, and this property has been heavily exploited.^17^ The 4,4′-BIPY ligand is an ideal connector between transition metal atoms for the propagation of coordination networks. In principle, the pyridyl groups can rotate along a central C–C bond. As the rotation does not affect the mutual orientation of the two lone pairs 4,4′-BIPY may be regarded in this context as rigid and a prototypical bridging ligand.^16^

Another, steadily increasing application of 2,2′-BIPY and 4,4′-BIPY in crystal engineering and related areas is their use as coformers for cocrystallization.^17−22^ The amino groups make them capable of forming strong H-bonding interactions with comolecules exhibiting H-bonding donor groups. Several pharmaceutical cocrystals have been described (*e.g*., sulfamethoxazole-4,4′-bipyridine,^23^ lamotrigine-2,2′-bipyridine,^24^ lamotrigine-4,4′-bipyridine,^24^*etc.*), for which the cocrystal modifies and improves key physicochemical properties, such as solubility, bioavailability, stability, dissolution rates, and mechanical properties.^25−28^ For example, the apigenin-4,4′-bipyridine cocrystal shows a higher solubility and, thus, enhanced bioavailabilty.^29^

In this work, we explore the solid-form landscapes of 2,2′-BIPY and 4,4′-BIPY. For the first compound, 2,2′-BIPY, only one form (anhydrate – **AH**_**22**_) has been reported in the literature and its structure was solved several times (CSD^30^-refcode family: BIPYRL).^31−34^ In contrast, 4,4′-BIPY is known to form an anhydrate (**AH**_**44**_, HIQWEJ^18,35−37^), dihydrate (**Hy2**_**44**_, WOVYEL^38−41^), and disolvates with formic acid (**S-C1**_**44**_, GOKCEQ^42^), as well as acetic acid (**S-C1**_**44**_, SITDIJA^43^). For all forms, the structures are known, but no stability data or phase interrelationships of the forms have been reported so far. Therefore, our experimental solid-form screen aimed at finding and characterizing the solvate forms of the selected bipyridines. Structural features were derived from single-crystal X-ray diffraction. Gravimetric moisture (de)sorption experiments, thermal analytical methods, and isothermal calorimetry provided the kinetic and thermodynamic stability data. The experimental findings were contrasted to computational investigations (CSP, structure minimizations, and intermolecular energy calculations). A successful CSP study had already been undertaken for 2,2′-BIPY, with the experimental form being the second lowest energy structure.^44^ Hence, we provide a more complete picture of the solid-state forms (five literature forms and seven new solvates) and their interconversion pathways, which is a prerequisite for the safe handling, production and storage of a fine chemical.

## 2 Materials and Methods

### 2.1 Potential Energy Surface Scans and Cambridge Structural Database (CSD) Analysis

PES scans were performed at the B3LYP/6-31G(d,p) level of theory using GAUSSIAN09.^45^ The dihedral angles specified in Figure 1 were scanned in 10°, 20°, or 30° steps.

The Cambridge Structural Database (version 5.42 plus updates 1–2)^30^ was searched for error-free, polymer-free, organic crystal structures of 2,2′-BIPY, 4,4′-BIPY, and carboxylic acids and dihedral angles were analyzed.

### 2.2 Computational Generation of the Anhydrate Crystal Energy Landscapes

The conformational analysis of 2,2′-BIPY and 4,4′-BIPY revealed that 2,2′-BIPY has one minimum within the intramolecular energy range of 29 kJ mol^–1^, which was chosen for a Z′ = 1 CSP search. For 4,4′-BIPY, the minimum conformation and additionally a maximum (only 6 kJ mol^–1^ less stable and the most frequent conformer seen among the CSD structures) were chosen for Z′ = 1 and Z′ = 2 searches. The choice of sampling Z′ = 1 and Z′ = 2 was based on the experimental findings.

The searches were performed using CrystalPredictor v2,^46−48^ covering the 59 most common space groups and keeping the molecular geometry rigid. The structures were first optimized with DMACRYS (covering the 19 kJ mol^–1^ range with respect to the search minimum), and then the pyridyl–pyridyl dihedral was optimized with CrystalOptimizer v2.4.6^49^ (197 2,2′-BIPY and 179 4,4′-BIPY structures) using a distributed multipole representation of the charge density^50^ within DMACRYS.^51^ The conformational energies and distributed multipoles used were calculated at the PBE0/6-31G(d,p) and PBE0/aug-cc-pVTz levels using Gaussian09,^45^ respectively, and all other intermolecular forces were modeled in an atom–atom *exp-6* form using the FIT potential.^51,52^

The most stable structures (60 2,2′-BIPY and 71 4,4′-BIPY) were then optimized with CASTEP v20.11,^53^ using the Perdew–Burke–Ernzerhof (PBE) generalized gradient approximation (GGA) exchange-correlation density functional^54^ and ultrasoft pseudopotentials,^55^ with the addition of the Tkatchenko and Scheffler (TS)^56^ semiempirical dispersion correction. The number of *k*-points were chosen to provide a maximum spacing of 2π·0.07 Å^–1^, and a basis set cutoff of 780 eV was applied. Optimizations were considered complete when energies were converged to better than 2 × 10^–5^ eV per atom, atomic displacements to 1 × 10^–3^ Å, maximum forces to 5 × 10^–2^ eV Å^–1^, and maximum stresses to 0.1 GPa. The energies of selected structures were recalculated, without optimization, using the same settings except for a basis set cutoff of 1100 eV and the MBD* dispersion correction.^57^ Isolated molecule minimizations to compute the isolated energy (*U*_gas_), necessary for calculating *E*_latt_, were performed by placing a single molecule in a fixed cubic 25 × 25 × 25 Å^3^ unit cell and then optimized with the same settings as used for the crystal calculations. For more details, see section 3.2 of the Supporting Information.

COMPACK^58^ and the CCDC API packing similarity dendrogram script were used for clustering the structures.

### 2.3 Intermolecular and Cluster Energy Calculations

The PBE-TS structures were used as starting points for pairwise intermolecular energy calculations,^59−61^ using CrystalExplorer v17^62^ and a 3.8 Å radius. B3LYP/6-31G(d,p) molecular wave functions, derived using Gaussian16,^63^ were employed to calculate the densities of unperturbed monomers to obtain the four separate energy components: electrostatic (*E*_E_), polarization (*E*_P_), dispersion (*E*_D_), and exchange-repulsion (*E*_R_).

### 2.4 Relative Stability Calculations of the Solvates

The method proposed by Dudek and Day^64^ was applied to compare the lattice energies between solvates and anhydrates. The energies of the solvent molecules were estimated by calculating the lattice energies of their crystalline phases (C1 – FORMAC01;^65^ C2 – ACETAC03;^66^ C3 – PRONAC;^67^ C4 – BUTRAC;^68^ C5 – VALRAC;^69^ C6 – ISENID;^70^ C8 – ISENUP^70^). An energy correction equal to 3/2 *RT* at 300 K was applied to account for the internal change of the solvent molecule in the liquid and solid state.^71,72^ The comparable BIPY energies were calculated according to eq 1
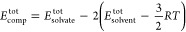
1where *E*_solvate_^tot^ and *E*_solvent_^tot^ correspond to the calculated lattice energies of the solvates and crystal structures of the solvent molecules (2 for disolvates).

### 2.5 Experimental Solid-Form Screen and Preparation of BIPY Solid-State Forms

2,2′-BIPY was bought from Sigma-Aldrich (≥99%) and 4,4′-BIPY from Aldrich (≥98%).

Evaporation, slurry, and cooling crystallization experiments were employed to screen for 2,2′-BIPY and 4,4′-BIPY solid-state forms using 40 different solvents. Details about the experiments are provided in section 1 of the Supporting Information.

The anhydrates (**AH**_**22**_ and **AH**_**44**_) were obtained in slurry experiments using organic solvents (except from solvate forming solvents and in the case of 4,4′-BIPY additionally from solvents with a water activity >0.35). Furthermore, **AH**_**44**_ was prepared by storing **Hy2**_**44**_ at a relative humidity (RH) < 35% at room temperature (RT).

**Hy2**_**44**_ was produced in cooling crystallization experiments and slurry experiments (10–30 °C) using water as solvent or by storing **AH**_**44**_ at RH > 35%.

The solvates (**S-C1**_**22**_, **S-C1**_**44**_, **S-C2**_**44**_, **S-C3**_**44**_, **S-C4**_**44**_, **S-C5**_**44**_, **S-C6**_**44**_, and **S-C8**_**44**_) were prepared either in slurry experiments starting from the anhydrates and stirring the bipyridines in carboxylic acids in the temperature range between 10 and 30 °C or cooling crystallization experiments (saturated solutions close to the boiling point of the solvents and naturally cooled to RT).

### 2.6 Single-Crystal and Powder X-ray Diffraction

#### 2.6.1 Single-Crystal X-ray Diffraction

The cooling crystallization experiments from the respective carboxylic acids yielded **S-C1**_**22**_, **S-C3**_**44**_, **S-C4**_**44**_, **S-C5**_**44**_, **S-C6**_**44**_, and **S-C8**_**44**_. At first glance, the majority of the crystals seemed to be of high quality. However, upon the slightest touch with a needle cleavage and polysynthetic twinning was observable for **S-C3**_**44**_, **S-C4**_**44**_, **S-C5**_**44**_, and **S-C6**_**44**_. The more stress that was applied, the more evident the twinning. Fragments of presumable untwinned crystals were chosen. Intensity data were recorded at 193 K, using a Rigaku Oxford Diffraction CCD Gemini Ultra diffractometer and Mo Kα radiation (λ = 0.71073 Å). The data were corrected for absorption effects using CrysAlis.^73^ The crystal structures were solved with SIR-2019^74^ and refined by full-matrix least-squares techniques using SHELXL.^75^ The polar H atoms and H atoms of the formic acid (of **S-C1**_**22**_) were identified from difference Fourier maps and refined with difference restraints (DFIX), the remaining H atoms were included in the refinement using a riding model. The hydrocarbon moieties in **S-C3**_**44**_ and **S-C6**_**44**_ were found to be disordered over two positions (**S-C3**_**44**_ – 0.60:0.40; **S-C6**_**44**_ – 0.34:0.66). All disorder components were refined using distance restraints for chemically equivalent C–C distances. For **S-C4**_**44**_, disorder was evident, but the quality of the data did not allow the disorder refinement, and therefore, only the major component was refined and data is provided as Supporting Information.

#### 2.6.2 Powder X-ray Diffraction

PXRD patterns were obtained using an X’Pert PRO diffractometer (PANalytical, Almelo, NL) equipped with a θ/θ coupled goniometer in transmission geometry, Cu K_α1,2_ radiation source and a solid state PIXcel detector. The patterns were recorded at a tube voltage of 40 kV and tube current of 40 mA, applying a step size of 2θ = 0.013° with 200 or 400 s per step in the 2θ range between 2° and 40°. For nonambient RH measurements, a VGI stage (VGI 2000M, Middlesex, UK) was used.

### 2.7 Thermal Analysis and Isothermal Calorimetry

Hot-stage microscopic investigations were performed using a BH2 polarizing microscope (Olympus, A), equipped with a Kofler hot-stage (Reichert, Austria) and an Olympus DP71 digital camera.

For thermogravimetric analysis (TGA) a TGA7 system (PerkinElmer, Norwalk, CT, USA) using the Pyris 8.0 Software was employed. Approximately 3 to 5 mg of sample was weighed into a platinum pan. Two-point calibration of the temperature was performed with ferromagnetic materials (Alumel and Ni, Curie-point standards, PerkinElmer). Heating rates of 0.5, 2, and 5 °C min^–1^ were applied and dry nitrogen was used as a purge gas (sample purge: 20 mL min^–1^, balance purge: 40 mL min^–1^).

Differential scanning calorimetry (DSC) was carried out with a DSC7 (PerkinElmer, Norwalk, Connecticut, USA) controlled by the Pyris 8.0 software. Approximately 1.5 to 3 mg of sample was weighed into perforated or sealed aluminum pans using a UM3 ultramicrobalance (Mettler, Greifensee, Switzerland). Heating rates of 0.5, 2, and 5 °C min^–1^ were applied and dry nitrogen was used as a purge gas (20 mL min^–1^). The instrument was calibrated for temperature with pure benzophenone (mp 48.0 °C) and caffeine (236.2 °C), and the energy calibration was performed with indium (mp 156.6 °C, heat of fusion 28.45 J g^–1^). The errors on the stated (extrapolated onset) temperatures and enthalpy values were calculated at 95% CIs and are based on at least three measurements.

Isothermal calorimetry (flow control) experiments using an RH perfusion cell were performed with the TAM III nanocalorimeter (TA Instruments, Eschborn, Germany). Four milliliter stainless steel ampules were used, and the RH was controlled with two mass flow controllers (dry N_2_ flow rate of 100 mL h^–1^). Freshly prepared **AH**_**44**_ (approximately 15 mg) was used, and the RH increased in one step from 20% to 80%. The RH was calibrated with saturated solutions of NaCl (75.3% RH) and LiCl (11.3% RH). Baseline stability was set to ±25 nW. The heat flow of the empty RH perfusion ampule (baseline runs with the same humidity steps) was subtracted from the heat flow of the sample measurements. The error on the stated hydration enthalpy was calculated at the 95% CI and is based on five measurements.

### 2.8 Infrared Spectroscopy

Temperature-controlled IR spectroscopy was carried out with a diamond ATR (PIKE GaldiATR) crystal on a Bruker Vertex 70 FTIR spectrometer (Bruker Analytische Messtechnik GmbH, D). The spectra were recorded between 4000 and 400 cm^–1^ at an instrument resolution of 2 cm^–1^ (32 scans per spectrum).

### 2.9 Gravimetric Moisture (De)sorption Experiments

The moisture (de)sorption isotherms were acquired using the automatic multisample gravimetric moisture sorption analyzer SPS23-10 μ (ProUmid, Ulm, D). The measurement cycles were started at ∼0% relative humidity (RH), increased in 5% steps to 95% RH (sorption), and then decreased in 5% steps to ∼0% RH (desorption). 200 ± 50 mg of sample was used for each analysis. The equilibrium conditions for each step were set to a mass constancy of ±0.001% over 60 min and a maximum time limit of 48 h. The instrument was calibrated according to the suppliers’ recommendations.

## 3 Results and Discussion

### 3.1 Experimental and Computational Screen for Solid-State Forms

The experimental screen for 2,2′-BIPY and 4,4′-BIPY solid forms using 40 solvents reproduced the known forms of the two isomers, **AH**_**22**_, **AH**_**44**_, **Hy2**_**44**_, **S-C1**_**44**_, and **S-C2**_**44**_. Furthermore, six novel solvates emerged from the evaporation, cooling crystallization and slurry experiments, namely, the only solvate of 2,2′-BIPY (**S-C1**_**22**_) and five additional carboxylic acid disolvates of 4,4′-BIPY (**S-C3**_**44**_, **S-C4**_**44**_, **S-C5**_**44**_, **S-C6**_**44**_, and **S-C8**_**44**_), as confirmed with X-ray diffraction and thermo-analytical methods. **AH**_**22**_ readily crystallized in the form of hexagonal plates, **AH**_**44**_ in hexagonal or rectangular plates, and **Hy2**_**44**_ in the shape of needles. The majority of the solvates formed big platy crystals (as illustrated in the Supporting Information, section 5).

**Figure 2:**
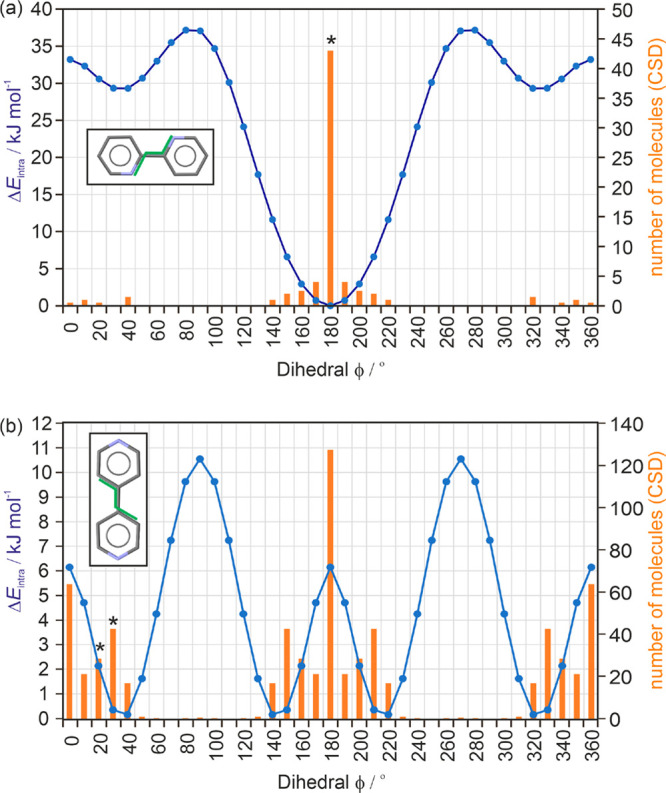
Potential energy surface scans of (a) 2,2′-BIPY and (b) 4,4′-BIPY were performed at the B3LYP/6-31G(d,p) level of theory, and the number of conformers found in the CSD (redeterminations excluded) are shown. Torsion angles scanned are highlighted in green in the insets. Asterisks (*) denote the conformers present in the experimental anhydrates. Note that due to the symmetry of the BIPY molecules, most conformers are present twice for 2,2′-BIPY and four times for 4,4′-BIPY in the 360° scans. Conformers were distributed uniformly, i.e., each conformer is counted only once, and therefore the experimental conformers are marked only once.

Potential energy surface scans were performed to investigate the conformational flexibility of the two isomers and the results contrasted to CSD searches. In the vast majority of the known 2,2′-BIPY structures, the molecule adopts the global minimum conformer, including **AH**_**22**_ (Figure 2a). In numerous structures, the C(pyridyl)–C(pyridyl′) bond lies in a special position (inversion symmetry). In only very few of the structures, a conformation close to that of the local minimum around 35° is seen, and only if the formation of strong H-bonding interactions with comolecules requires the deviation from planarity. The local minimum was calculated to be approximately 30 kJ mol^–1^ less stable than the global minimum and, therefore, was not considered in the CSP searches.

In contrast, the PES scan of 4,4′-BIPY shows four minima, which are identical due to the symmetry of the molecule. Interestingly, the majority of the structures relate to a maximum (ϕ = 180) because of internal inversion symmetry in 4,4′-BIPY. The maximum was calculated to be only 6 kJ mol^–1^ less stable in intramolecular energy than the global minimum and, therefore, considered in the CSP searches. The **AH**_**44**_ conformers, a Z′ = 2 structure, are located in proximity to the global minimum and are marked with (*) in Figure 2b. Thus, the energy barriers of the two isomers for a 360 °C(pyridyl)–C(pyridyl′) rotation vary considerably (in gas phase), as does the number of CSD structures, with 4,4′-BIPY being found more frequently than 2,2′-BIPY (Figure 2).

**Figure 3:**
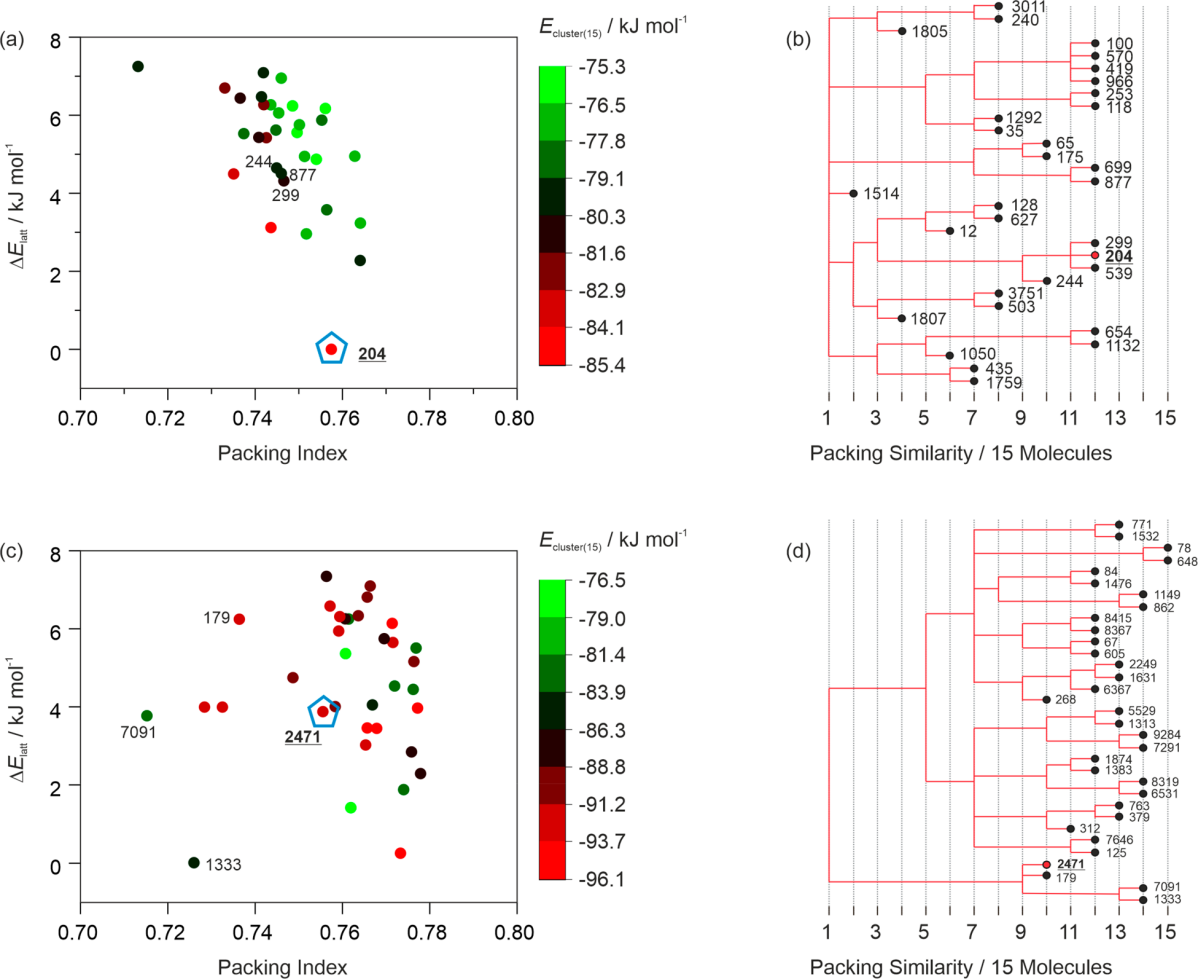
PBE-TS lattice energy landscape for (a) 2,2′-BIPY and (c) 4,4′-BIPY classified by the stability of 15 molecule clusters [B3LYP/6-31G(d,p) level of theory]. Each symbol denotes a crystal structure corresponding to a lattice energy minimum. Experimental structures are highlighted with a pentagon and selected hypothetical structures are labeled. (b,d) Structure family trees, showing the crystal packing similarity between the lowest-energy crystal structures from the CSP searches. Enlarged versions of the figures are provided in section 3.3 of the Supporting Information.

Crystal structure prediction was employed to complement the experimental findings and to contrast and rationalize the crystallization behavior of the two isomers. The 2,2′-BIPY lattice energy landscape presented in this study differs from an earlier study^44^ in that periodic electronic structure calculations have been applied as the final step. The most stable structure in the 2,2′-BIPY crystal energy landscape corresponds to the observed anhydrate and was calculated to be 2.3 kJ mol^–1^ more stable than the second lowest energy structure (Figure 3a). Overall, all of the computationally generated anhydrate structures show a high packing efficiency. The *CrystalExplorer* method was used to investigate the strengths of pairwise intermolecular interactions and intermolecular cluster energies (denoted as *E*_cluster_ hereafter). For **AH**_**22**_, the considered cluster (central 2,2′-BIPY molecule and 14 nearest neighbors) was estimated as the most stable of all computationally generated structures. Interestingly, the hypothetical structures showing slightly lower packing indices exhibit a lower cluster energy than the marginally denser packed structures. The 2,2′-BIPY structures lack, due to the absence of H-bonding donor groups, the ability to form strong H-bonding interactions. Thus, the strongest intermolecular interactions are of aromatic nature (π···π stacking < −27 kJ mol^–1^ in pairwise energy).

The facts that the *E*_latt_ and *E*_cluster_ calculations have the experimental form as the most stable and that an energy gap exists between the global minimum and other low-energy structures rationalize the easily crystallizing **AH**_**22**_. The packing similarity dendrogram (Figure 3b), calculated for the 31 most stable computed structures, indicates a broad packing diversity. The existence of alternative 2,2′-BIPY polymorphs cannot be excluded based on the (limited) CSP search. Structures 299 and 539 exhibit a 2D packing similarity^76^ with the experimental form (204 = **AH**_**22**_), sharing the same type of 2,2′-BIPY double layers (as illustrated in the Supporting Information, Figure 20). Furthermore, structures 244 and **AH**_**22**_ also have 2,2′-BIPY double layer sheets in common.

Among the 33 lowest-energy 4,4′-BIPY structures (Supporting Information, Tables S8) only 14 were found to be Z′ = 1, 8 Z′ = 2, and the remaining structures Z′ = 0.5 or 0.25 (11 structures). The lattice energy landscape (Figure 3c) has numerous structures, which were calculated to be more stable than **AH**_**44**_ indicating that alternative packing arrangements are feasible. Using PBE-MBD* instead of PBE-TS did not qualitatively improve the results, and therefore we decided to use the PBE-TS landscapes as a basis for the discussion of the differences between the two molecules.

The direct comparison between the lattice energy landscapes of the two isomers indicates that 4,4′-BIPY is at higher risk to be polymorphic, because (i) the lowest-energy structures are not observed and (ii) there are more structures within the lowest-energy range than for 2,2′-BIPY. Another feature of the 4,4′-BIPY lattice energy landscape is that the lowest-energy structures show slightly lower packing indices, compared to 2,2′-BIPY. The latter trend has been indicative of solvate and nonsolvate formation in structurally related molcules.^77^ Indeed, 4,4′-BIPY forms a hydrate and more solvates than 2,2′-BIPY. Nevertheless, the fact that in the 4,4′-BIPY molecule the aromatic N atoms are exposed and can act more easily as H-bonding acceptors than those in 2,2′-BIPY explains the higher tendency toward solvate (hydrate) formation for 4,4′-BIPY.

The structure similarity calculations (Figure 3d) reveal that **AH**_**44**_ shows packing similarity with several of the “lower” density structures. Structures 2471 (**AH**_**44**_), 179, 7091, and 1333 (lowest energy structure) form the same 2D layer of 4,4′-BIPY molecules. In adjacent layers, selected molecules deviate in orientation (Supporting Information, Figure S22). The 4,4′-BIPY structures show a higher degree of similarity with each other than the 2,2′-BIPY structures. A high proportion of 4,4′-BIPY structures differs only in the stacking of layers, i.e., subtle differences in close contact interactions. The chosen method(s) for deriving the lattice energy landscapes in this study do not provide a satisfactory result for 4,4′-BIPY. Reasons therefore might be that (i) alternative polymorphs exist and/or (ii) some of the structurally related low-energy structures are artifacts of approximating the free energy.^78^ More accurate methods are needed, going beyond (relative) lattice energy calculations, i.e., free energy approximations,^79^ but this was not the aim of this work.

### 3.2 Crystal Structures of 2,2′- and 4,4′-Bipyridine

The single crystal structures of **AH**_**22**_, **AH**_**44**_, **Hy2**_**44**_, **S-C1**_**44**_, and **S-C2**_**44**_ have already been reported and are discussed together with the new crystal structures of **S-C1**_**22**_, **S-C3**_**44**_, **S-C4**_**44**_, **S-C5**_**44**_, **S-C6**_**44**_, and **S-C8**_**44**_ below (Table 1).

**Table 1:** Compilation of Selected Crystallographic Data of 2,2′-BIPY and 4,4′-BIPY Solid-State Forms

	**AH_22_** (BIPYRL04)	**S-C1_22_**	**AH_44_** (HIQWEJ03)	**Hy2_44_** (WOVYEL02)	**S-C1_44_** (GOKCEQ)	**S-C2_44_** (SITDIJ)	**S-C3_44_**	**S-C4_44_**	**S-C5_44_**	**S-C6_44_**	**S-C8_44_**
Empirical formula	C_10_H_8_N_2_	C_10_H_8_N_2_·2 (CH_2_O_2_)	C_10_H_8_N_2_	(C_10_H_8_N_2_ ·2 (H_2_O)	C_10_H_8_N_2_·2 (CH_2_O_2_)	C_10_H_8_N_2_·2 (C_2_H_4_O_2_)	1/2(C_10_H_8_N_2_)·(C_3_H_6_O_2_)	1/2(C_10_H_8_N_2_)·(C_4_H_8_O_2_)	1/2 (C_10_H_8_N_2_)·(C_5_H_10_O_2_)	1/2 (C_10_H_8_N_2_)·(C_6_H_12_O_2_)	C_10_H_8_N_2_·2 (C_8_H_16_O_2_)
Formula weight	156.18	248.24	312.37	192.22	248.24	355.390	152.17	166.2	180.22	194.25	444.60
Temperature	123	193(2)	150	93	296(2)	291(2)	193(2)	193(2)	193(2)	193(2)	193(2)
Crystal system	monoclinic	orthorhombic	triclinic	monoclinic	monoclinic	monoclinic	triclinic	triclinic	triclinic	triclinic	triclinic
Space group	*P*2_1_/*n*	*Pbc*2_1_	*P*1̅	*P*2_1_	*P*2_1_	*Pc*	*P*1̅	*P*1̅	*P*1̅	*P*1̅	*P*1̅
*a*/Å	5.4862(1)	3.7087(6)	8.693(2)	9.12140(10)	3.786(5)	3.893(2)	5.2183(6)	5.230(3)	5.1355(11)	5.0207(7)	8.9108(9)
*b*/Å	6.1657(1)	15.9130(2)	8.735(2)	7.41040(10)	7.938(10)	8.181(5)	6.2686(7)	6.240(2)	6.3797(12)	6.654(1)	9.6304(12)
*c*/Å	11.6094(3)	19.8320(3)	10.982(2)	14.72100(10)	20.94(3)	22.563(15)	13.0250(2)	14.119(10)	15.658(4)	16.974(2)	16.4710(2)
α/°	90	90	85.14(2)	90	90	90	96.043(1)	87.52(4)	82.443(19)	100.654(12)	98.997(11)
β/°	95.276(1)	90	85.37(2)	100.9910(10)	95.16(3)	98.46(3)	100.824(1)	83.59(5)	89.93(2)	93.111(12)	93.962(10)
γ/°	90	90	98.95(2)	90	90	90	103.952(1)	78.10(4)	80.595(17)	97.602(12)	113.384(11)
Volume/Å^3^	391.04(1)	1170.42(19)	813.80(3)	976.788(19)	626.8(14)	710.7(7)	401.04(6)	448.0(4)	501.60(19)	550.58(13)	1267.9(2)
Z/Z′	2/0.5	4/1	4/2	4/2	2/1	2/1	2/1	2/1	2/1	2/1	2/1
Density (calc)/g cm^–3^	1.326	1.409	1.275	1.307	1.315	1.291	1.260	1.232	1.193	1.172	1.165
No. of measured, independent and observed [*I* > 2σ(*I*)] reflections	5555, 712, 668	5849, 2462, 1613	13284, 13284, 10401	65832, 3535, 3165	2246, 2185, 1474	6467, 1595, 995	2822, 1846, 1282	2825, 1838, 586	3024, 2036, 1032	3567, 2310, 1309	9155, 5833, 3635
*R*_int_	0.031	0.066	a	0.043	0.033	0.042	0.020	0.053	0.040	0.027	0.050
(sin θ/λ)_max_ (Å^–1^)	0.602	0.670	1.000	0.602	0.661	0.649	0.701	0.676	0.683	0.687	0.705
*R*[*F*^2^ > 2σ(*F*^2^)], *wR*(*F*^2^), *S*	0.030, 0.079, 1.05	0.08, 0.226, 1.06	0.047, 0.150, 1.02	0.020, 0.028, 0.76	0.048, 0.128, 1.05	0.050, 0.119, 1.04	0.051, 0.147, 1.03	0.119, 0.397, 0.98	0.064, 0.168, 1.00	0.056, 0.155, 0.98	0.054, 0.145, 1.02
No. of reflections	712	2462	13284	3240	2185	1595	1846	1838	2036	2310	5833
No. of parameters	72	177	281	253	211	185	126	112	124	180	300
No. of restraints	0	7	16	0	1	2	37	0	1	127	2
Largest diff. peak and hole/e Å^–3^	0.15, −0.15	0.53, −0.42	0.50, −0.23	0.10, −0.19	0.13, −0.16	0.15, −0.12	0.16, −0.21	0.34, −0.30	0.20, −0.24	0.16, −0.15	0.27, −0.21

a Information not provided.

**Figure 4:**
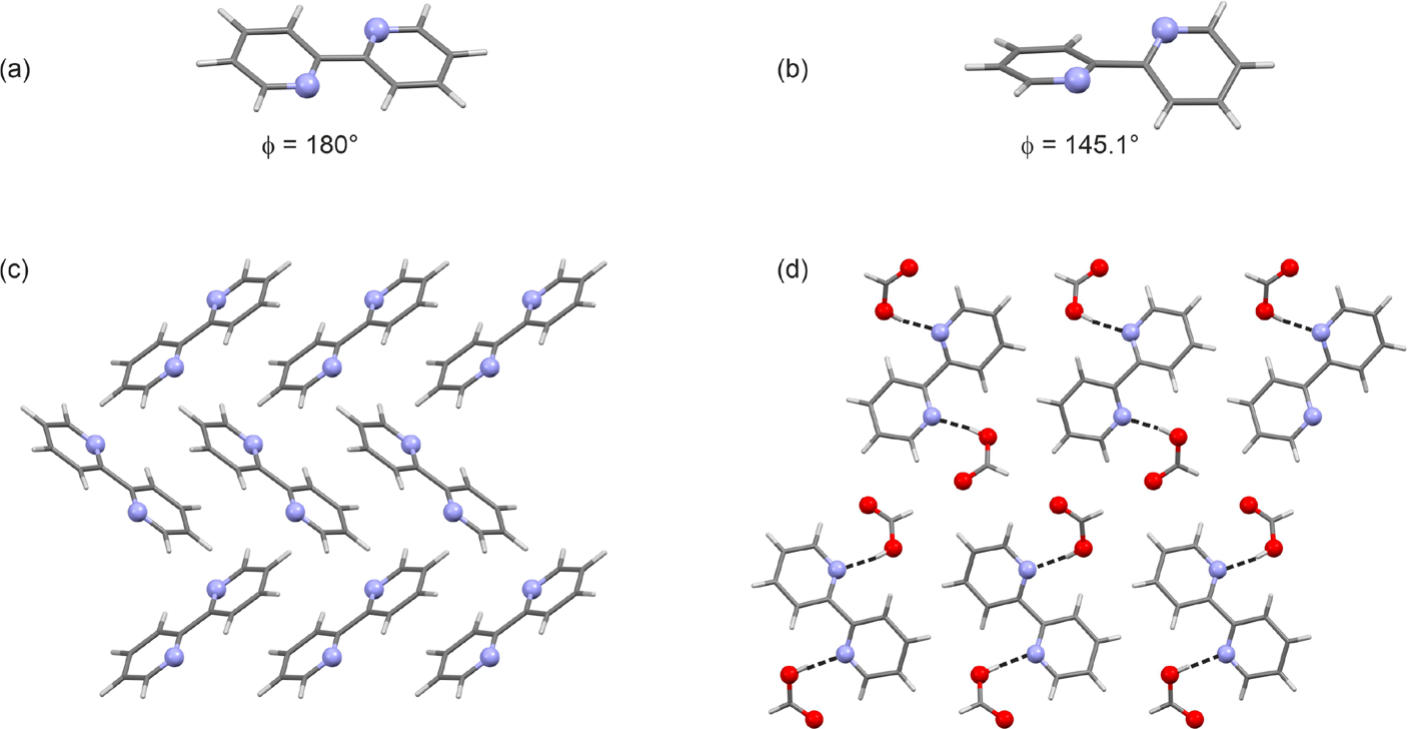
2,2′-BIPY conformers observed in (a) **AH**_**22**_ and (b) **S-C1**_**22**_. Packing diagrams of (c) **AH**_**22**_ and (d) **S-C1**_**22**_ are viewed along the respective crystallographic *a* axes. Strong H-bonding interactions are highlighted with dotted lines in (d).

The anhydrate of 2,2′-BIPY crystallizes in the monoclinic space group *P*2_1_/*n* with Z′ = 0.5. The 2,2′-BIPY molecule lies on a special position, a center of inversion, and adopts the planar global conformational energy minimum (Figures 2a and 4a). The 2,2′-BIPY molecules are stacked along the *b* crystallographic axis leading to aromatic interactions (π···π stacking), which were calculated to be the strongest pairwise intermolecular interactions in the structure (−18.3 kJ mol^–1^). Adjacent stacks of 2,2′-BIPY molecules are tilted by 69.4° (Figure 4c), and the pyridine N atoms form C–H···N interactions (−12.4 kJ mol^–1^).

The formic acid solvate of 2,2′-BIPY crystallizes in the orthorhombic space group *Pbc*2_1_ with one 2,2′-BIPY and two formic acid molecules in the asymmetric unit. In contrast to **AH**_**22**_, the 2,2′-BIPY conformer seen in **S-C1**_**22**_ (ϕ = 145.1°, Figure 4b) does not correspond to the global minimum (Figure 2a) and was calculated to be 11.2 kJ mol^–1^ less stable in intramolecular energy. The presence of a high energy 2,2′-BIPY conformation in **S-C1**_**22**_ can be related to the interplay of inter- and intramolecular interactions in the crystal structure. The intramolecular enthalpy penalty seen in **S-C1**_**22**_ is compensated for by the formation of strong intermolecular interactions. Each of the two acid molecules forms strong O–H···N H-bonding and C–H···O close contracts to 2,2′-BIPY (Figure 4d). The pairwise interactions were estimated to account for −56.7 and −56.4 kJ mol^–1^ in energy. Furthermore, π···π stacking strongly contributes to the stability of the solvate structure (−26.3 kJ mol^–1^, stacked along the *a* crystallographic axis). Adjacent H-bonded entities are related by 2_1_ and *b* glide plane symmetry, resulting in the **S-C1**_**22**_ packing.

**Figure 5:**
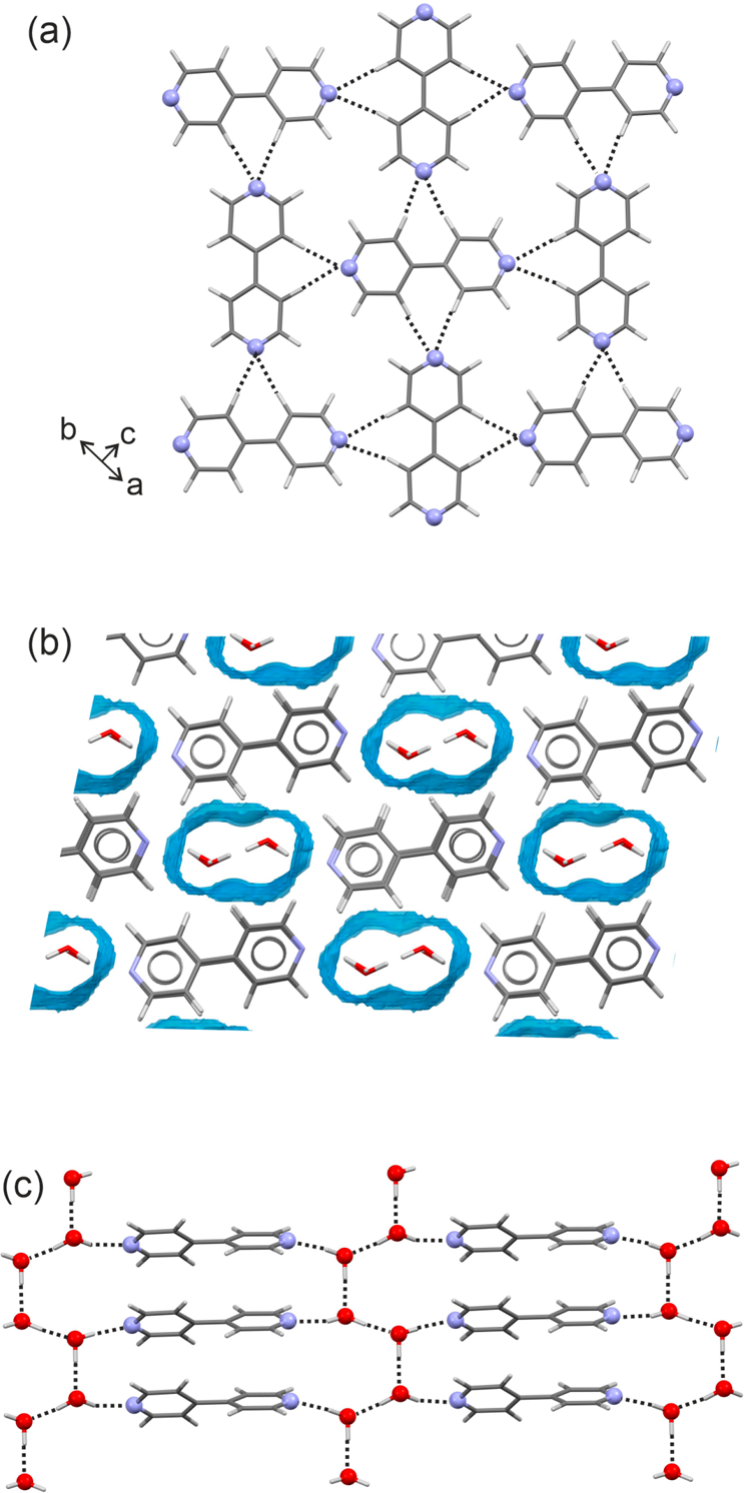
(a) Packing diagram of **AH**_**44**_. Dashed lines indicate the C–H···N interactions. (b) Water channels seen in **Hy**_**44**_ were calculated using the Hydrate Analyzer tool (probe radius: 1.0 Å, approximate grid spacing: 0.15 Å), viewed along the crystallographic *b* axis. (c) Strong H-bonding interactions (water channels) observed in **Hy**_**44**_ are depicted with dotted lines.

The **AH**_**44**_ adopts the triclinic *P*1̅ symmetry with Z′ = 2. The two conformers can be differentiated based on the dihedral angles between the connected pyridyl rings, as already reported.^35^ The conformers (ϕ approximately 19° and 35°) differ by only 0.6 and 1.8 kJ mol^–1^ in intramolecular energy from the gas-phase minimum (Figure 2b). The two crystallographically independent molecules form slightly corrugated layers, which are linked through bifractured C–H···N interactions (Figure 5a), with a pairwise energy of −18.4 to −21.7 kJ mol^–1^. Layer stacks are stabilized through aromatic interactions.

The **Hy**_**44**_ structure has been solved in three different monoclinic space groups, *P*2_1_ (Z′ = 2, WOVYEL,^38^ WOVYEL02^40^), *P*2_1_/*n* (Z′ = 1, WOVYEL01^39^), and *C*2 (Z′ = 0.5, WOVYEL03^41^). The *P*2_1_/*n* structure shows disorder of the 4,4′-BIPY and the *C*2 structure disorder in the water molecule positions. The 4,4′-BIPY molecule positions in the *P*2_1_ and *C*2 structures are identical, but the water molecule(s) differ in the *y* position (along the projection axis of Figure 5b). We have chosen the ordered *P*2_1_ structure for the discussion, as water proton positions are given and the energy calculations required an ordered structure. The dihedral angles between the connected pyridyl rings are approximately 41°. Each of the four water molecules forms an O–H···N H-bond to one of the 4,4′-BIPY molecules. The interactions between the water and the 4,4′-BIPY lead to 4,4′-BIPY···H_2_O···H_2_O···4,4′-BIPY chains, which proceed in the direction of the *c* axis. Furthermore, the water molecules are connected via O–H···N H-bonds to form two independent chains propagating parallel to the crystallographic *b* axis, each involving two of the four independent water molecules (Figure 8c). The combination of the H-bonded water chains leads to the formation of sheets, which are parallel to the *a* axis. The 4,4′-BIPY molecules form stacks (π···π interactions) in direction of the *b* axis, which were calculated as nearly equally as strong as the 4,4′-BIPY···H_2_O interactions (−24.6 vs −26 to −29 kJ mol^–1^).

**Figure 6:**
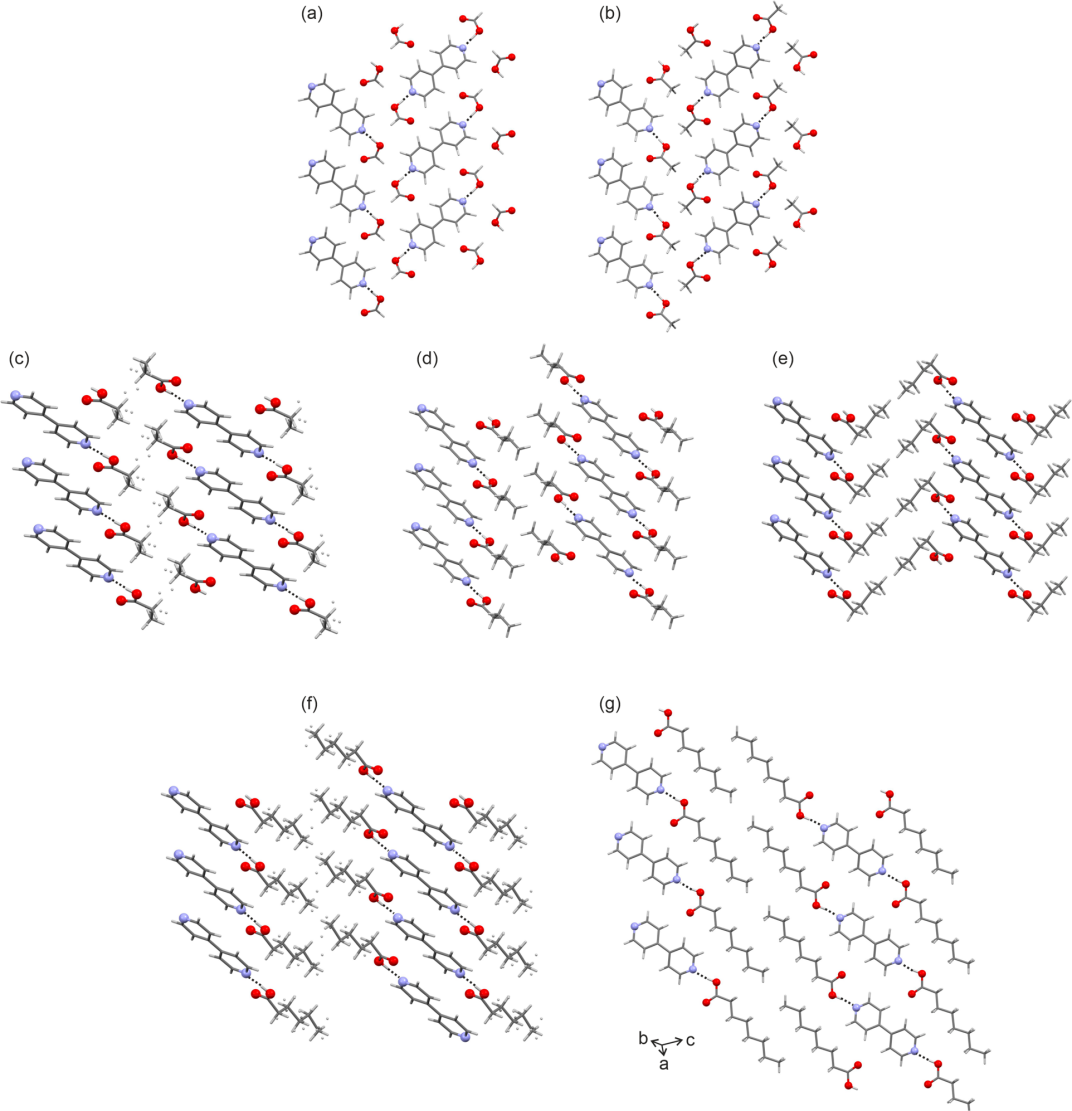
Packing diagrams of (a) **S-C1**_**44**_, (b) **S-C2**_**44**_, (c) **S-C3**_**44**_, (d) **S-C4**_**44**_, (e) **S-C5**_**44**_, (f) **S-C6**_**44**_, and (g) **S-C8**_**44**_, viewed along the crystallographic *a* (a,b) or *b* (c–f) axes. Dotted lines denote the strong H-bonding interactions between 4,4′-BIPY and the acids. For (c) and (f), alternative acid orientations (hydrocarbon parts) are indicated as small balls.

All of the BIPY solvates are disolvates, but crystallize in different space groups. The two literature solvates of 4,4′-BIPY, **S-C1**_**44**_ and **S-C2**_**44**_, both adopt the monoclinic crystal system, with each having 4,4′-BIPY and two solvent molecules in the asymmetric unit. The pyridyl rings are tilted by 29.5° to 32° in the two structures. The guest molecules, formic acid and acetic acid, are linked by strong O–H···N H-bonds. Adjacent H-bonded entities are either related by 2_1_ (**S-C1**_**44**_) or *c* glide plane symmetry (**S-C2**_**44**_), leading to highly related sheets (Figure 6a,b) and also very similar crystal structures. The sheets are stacked parallel to *a* and interact through aromatic (π···π) interactions. The acid···4,4′-BIPY H-bonds were identified as the strongest interactions, ranging from −49.6 to −53.3 kJ mol^–1^ (Table 2). Interestingly, the H-bonding interactions seen in the 4,4′-isomer are slightly higher in energy than the interactions seen in 2,2′-BIPY.

**Table 2:** Compilation of the Strongest Pairwise Interactions for Each Independent Acid Molecule in the 2,2′- and 4,4′-BIPY Carboxylic Acid Solvates

solvate	pairwise energy /kJ mol^–1^	interaction
**S-C1_22_**	–56.7	O–H···N and C–H···O
**S-C1_22_**	–56.4	O–H···N and C–H···O
**S-C1_44_**	–53.3	O–H···N and C–H···O
**S-C1_44_**	–51.2	O–H···N and C–H···O
**S-C2_44_**	–51.8	O–H···N and C–H···O
**S-C2_44_**	–49.6	O–H···N and C–H···O
**S-C3_44_**	–46.9	O–H···N and C–H···O
**S-C4_44_**	–49.3	O–H···N and C–H···O
**S-C5_44_**	–51.5	O–H···N and C–H···O
**S-C6_44_**	–50.9	O–H···N and C–H···O
**S-C8_44_**	–46.8	O–H···N and C–H···O
**S-C8_44_**	–46.2	O–H···N and C–H···O

The other five solvates, **S-C3**_**44**_, **S-C4**_**44**_, **S-C5**_**44**_, **S-C6**_**44**_, and **S-C8**_**44**_, crystallize in the triclinic *P*1̅ space group (Table 1). With the exception of **S-C8**_**44**_, the solvates have one acid and half a 4,4′-BIPY molecule in the asymmetric unit (inversion symmetry). Thus, the 4,4′-BIPY molecules in **S-C3**_**44**_, **S-C4**_**44**_, **S-C5**_**44**_, and **S-C6**_**44**_ adopt the planar maximum (ϕ = 180, Figure 2) conformation. In all of the solvate structures, the acid molecule forms the same type of strong H-bonding interactions as already discussed for **S-C1**_**44**_ and **S-C2**_**44**_. These interactions were calculated to account for −46.2 to −56.4 kJ mol^–1^ in intermolecular energy (Table 2). The strengths of the interactions can be seen as one of the driving forces for the high tendency of 4,4′-BIPY to form disolvates (and cocrystals) with carboxylic acids. A common packing feature of the triclinic 4,4′-BIPY solvates, but also of the monoclinic solvates, are the sheets built of 4,4′-BIPY and acid molecules, with the strong H-bonding interactions being located within the sheets (Figure 6). Stacking of the sheets proceeds in the direction of the *b* axis (**S-C3**_**44**_ to **S-C6**_**44**_) and the sheets interact through π···π contacts (4,4′-BIPY) and hydrocarbon close contacts of the acid molecules.

In **S-C3**_**44**_, the hydrocarbon tail of the propionic acid is disordered over two positions and refined in a ratio of 2:1. The conformation of the major component was found to be close to the optimized conformation of propionic acid, deviating only by 0.1 kJ mol^–1^ in intramolecular energy at the PBE0/6-31G(d,p) level of theory. The poor data quality (due to the features of the **S-C4**_**44**_ single crystals; see Materials and Methods) allowed only the refinement of one orientation of the butyric acid, but an alternative orientation is likely. The COOH function in the butyric acid deviates by approximately 50° from the stable gas phase conformation, which corresponds to approximately 2 kJ mol^–1^ (Supporting Information, Figure S3). The conformation of the valeric acid in **S-C5**_**44**_ deviates from the other acids in that valeric acid adopts a bent conformation, and thus, the acid molecules are not aligned with the 4,4′-BIPY molecules but form a zigzag motif with the host molecules (Figure 6e). The bent conformation was calculated to be 3.7 kJ mol^–1^ less stable than the elongated gas phase minimum (Supporting Information, Figure S5). The caproic acid solvate (**S-C6**_**44**_) is isostructural with **S-C3**_**44**_ and **S-C4**_**44**_ and the disorder ratio between two hydrocarbon orientations of the acid molecules refined to 3:2. The major disorder component adopts a conformation related to the COOH orientation seen in **S-C4**_**44**_ (+1.3 kJ mol^–1^ in intramolecular energy).

The pyridyl rings in **S-C8**_**44**_ are tilted by 16.7°; thus, no internal inversion symmetry relation is present. The conformations of the two caprylic acid molecules are very similar and closely related to the gas phase minimum. Another differentiation criterion between the **S-C8**_**44**_ and the other triclinic solvate structures is the orientation of the molecules within the sheets (Figure 6).

### 3.3 Anhydrates of 2,2′- and 4,4′-Bipyridine

**Figure 7:**
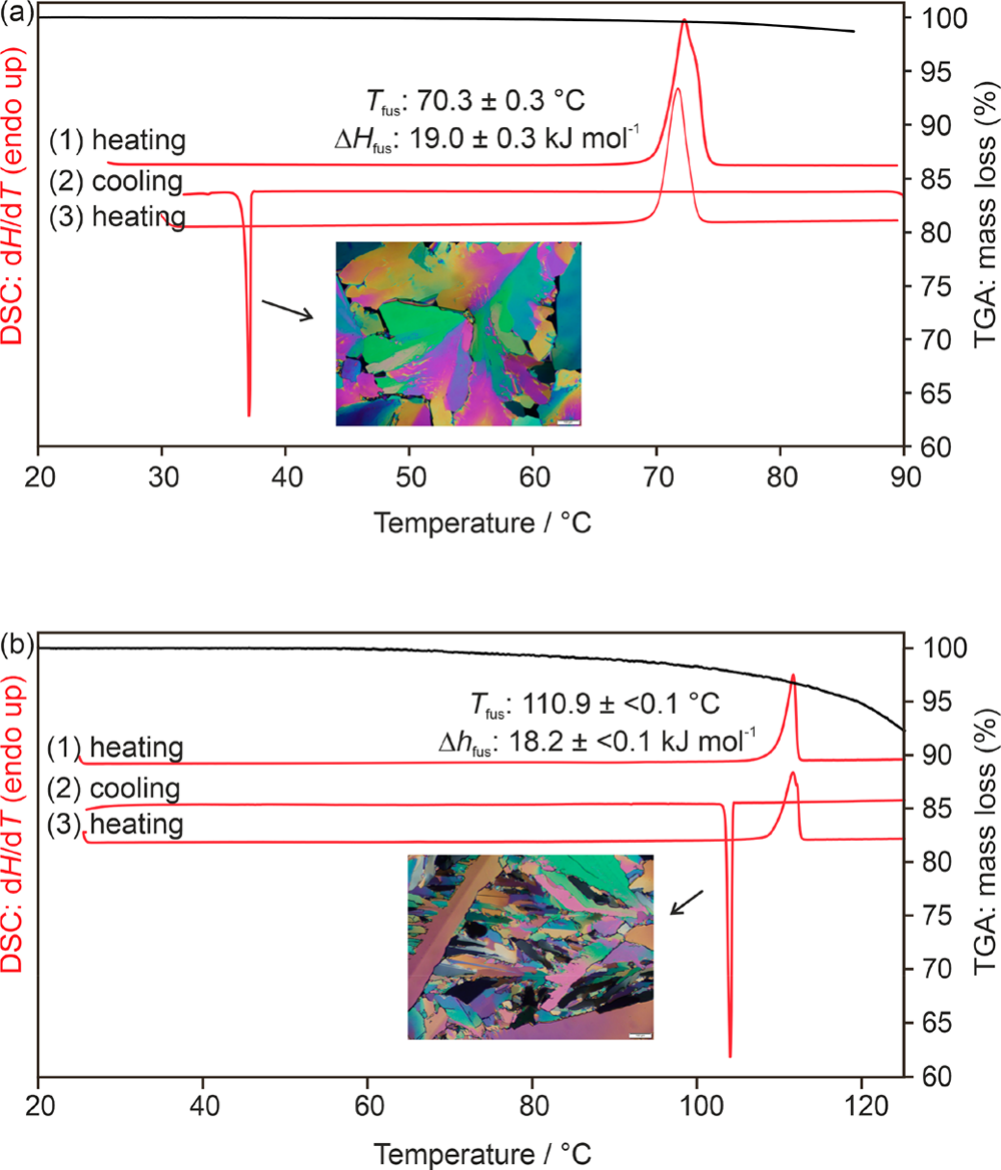
DSC and TGA thermograms of (a) 2,2′-BIPY and (b) 4,4′-BIPY anhydrates recorded at a heating and cooling rate of 5 °C min^–1^. Photomicrographs show the anhydrates crystallized from the melt.

The phase pure anhydrates of the two isomers, as confirmed with PXRD and IR spectroscopy (Supporting Information, section 7), were subjected to thermoanalytical measurements. Upon heating, **AH**_**22**_ shows one thermal event with an onset temperature at 70.3 °C, the melting point (*T*_fus_) of the compound (Figure 7a). The heat of fusion (Δ_fus_*H*) was measured to be 19.0 kJ mol^–1^. Recrystallization of the same anhydrous phase occurs upon cooling the melt at approximately 35 °C. Sublimation of **AH**_**22**_ starts at 55 °C and leads to a mass loss seen in TGA heating experiments.

The endotherm recorded in the **AH**_**44**_ DSC heating experiments corresponds to the melting of the anhydrate (*T*_fus_ = 110.9 °C, Δ_fus_*H* = 18.2 kJ mol^–1^). Upon cooling the melt, immediate recrystallization is observed at ca. 105 °C, and **AH**_**44**_ is obtained (Figure 7b). The mass loss observed in the TGA experiments corresponds to sublimation, which starts at ca. 60 °C. Neither sublimation nor annealing the melt of the two compounds facilitated the formation of a polymorph.

### 3.4 Solvates of 2,2′- and 4,4′-Bipyridine

All nine structurally characterized solvates (including **Hy2**_**44**_) crystallize in a stoichiometric ratio of 1:2 (BIPY:solvent). Each of the bulk crystallization products was confirmed to correspond to the disolvate by comparison of the experimental PXRD and the diffraction patterns simulated from the single-crystal structure (see Supporting Information, section 8). The samples used for the DSC and TGA analyses were of similar particle size.

#### 3.4.1 4,4′-Bipyridine Dihydrate

**Figure 8:**
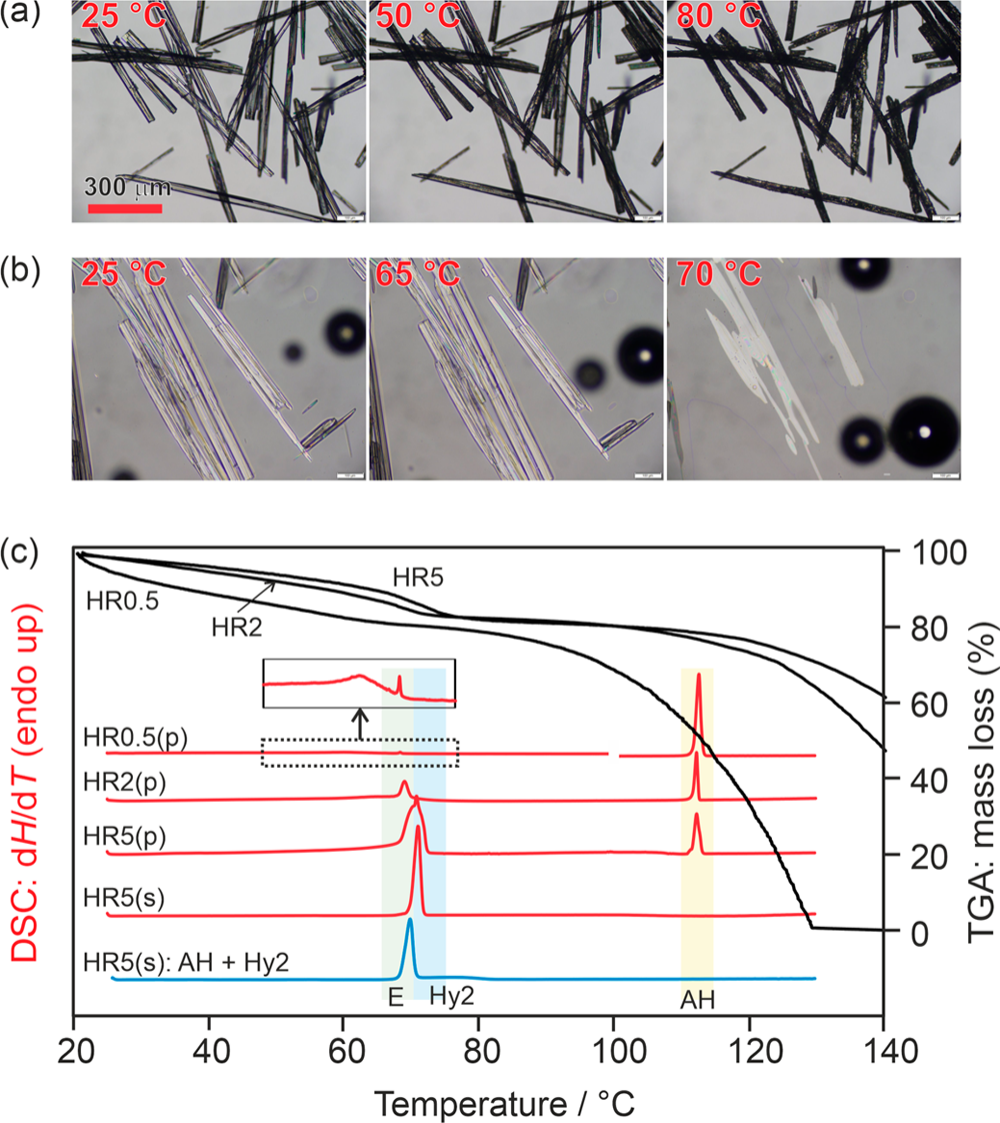
(a,b) Photomicrographs of **Hy2**_**44**_, showing the dehydration process (a) in a dry preparation and (b) embedded in high-viscosity silicon oil. (c) DSC and TGA thermograms of the hydrate recorded at heating rates (HR) of 0.5, 2, or 5 °C min^–1^ using open (TGA), perforated (p), or sealed (s) pans. The fifth DSC curve corresponds to a 1:1 mixture of **Hy**_**44**_ and **AH**_**44**_. Melting points of the anhydrate (AH), hydrate (Hy2), and the eutectic temperature (E) are highlighted.

The hydrate crystals (Figure 8a) turn opaque when slowly heated from RT to 80 °C, while the original shape of the crystals is maintained (called pseudomorphosis^80^). The latter phenomenon is characteristic for the desolvation of a stoichiometric solvate. The melting of the dehydration product (**AH**_**44**_) is observed at 111 °C. Heating **Hy2**_**44**_ crystals in a high-viscosity silicon oil embedding (Figure 8b) allowed the determination of the congruent melting point of the hydrate at 70–71 °C.

Based on the TGA mass loss of 18.68 ± 0.15%, which corresponds to 1.99 mol of water per mol 4,4′-BIPY, the dihydrate stoichiometry could be confirmed (theoretical value 18.75%). The 2 or 5 °C min^–1^ heating rates TGA curves show, starting from RT up to ca. 65 °C, a slow but continuous mass loss (Figure 8c). In the temperature range from 65 to 75 °C, a faster release of the water molecules is detectable (temperature region of the eutectic and hydrate melting point). Upon further increasing the temperature, a substantial mass loss can be seen, due to strong sublimation/evaporation of the compound, resulting in the evaporation of the entire sample up to 130 °C at a heating rate of 0.5 °C min^–1^. In contrast to the TGA curves, which were recorded in open pans, the DSC experiments were measured using pans with 5-pin-holed lids as well as hermetically sealed ones. Even at the lowest heating rate of 0.5 °C min^–1^, it was not possible to completely dehydrate **Hy2**_**44**_ in the DSC experiments. The peak at 68.5 °C corresponds to the eutectic between **AH**_**44**_ and **Hy2**_**44**_ (Figure 8c, fifth DSC curve) and can be detected in the **Hy2**_**44**_ DSC experiments using heating rates of 0.5 or 2 °C min^–1^ (5-pin-holed lid). The 5 °C min^–1^ heating rate experiment shows an overlap of the eutectic and melting of the hydrate at approximately 70 °C. The endotherm seen at ca. 111 °C corresponds to the **AH**_**44**_ melting peak. By using sealed DSC crucibles, it is possible to determine the homogeneous **Hy2**_**44**_ melting point at 69.9 °C.

**Figure 9:**
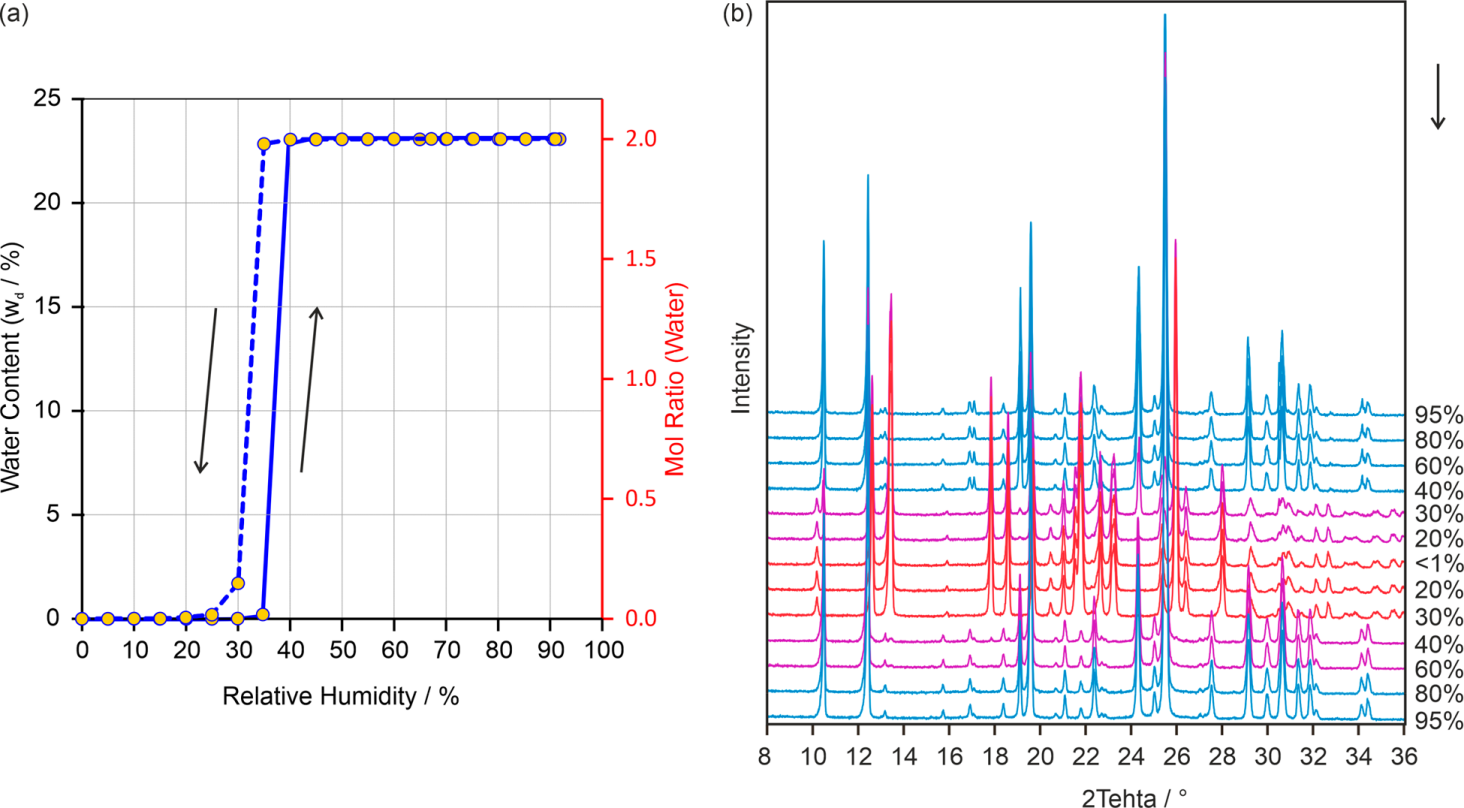
(a) Gravimetric moisture (de)sorption isotherms starting from **AH**_**44**_ at 25 °C. Arrows indicate the direction, i.e., sorption (full line) and desorption (dashed line). (b) Variable humidity PXRD experiments following the reversible **Hy**_**44**_ ↔ **AH**_**44**_ transformation at 25 °C. The arrow indicates the order of the subsequent measurements.

The stability of **AH**_**44**_ and **Hy2**_**44**_ was then investigated under different moisture conditions (0–95% relative humidity) at 25 °C. The **AH**_**44**_ absorbs water at a RH of 40% and transforms within approximately 2 h to **Hy2**_**44**_ (Figure 9a). No further mass uptake was seen upon increasing the RH to 95%. Upon decreasing the RH, dehydration occurs at an RH value of 30% within roughly two hours. From the very small hysteresis of the sorption and desorption curves, it can be deduced that the critical water activity for **AH**_**44**_ ↔ **Hy2**_**44**_ lies at 0.35. This information is crucial for handling, especially weighing, 4,4′-BIPY, as hydration and dehydration can occur at ambient conditions and at a relatively high rate. Another interesting fact derived from the (de)sorption data is that **AH**_**44**_ strongly sublimes at RT (mass loss due to sublimation was offset in Figure 9a). This finding is in contrast to **Hy2**_**44**_, which showed hardly any mass loss due to sublimation. Similar to **AH**_**44**_, the isomer **AH**_**22**_ shows sublimation at RT (Supporting Information, Figure S34).

The reversible **AH**_**44**_ ↔ **Hy2**_**44**_ transition was additionally investigated using RH dependent PXRD measurements. The RH was decreased and increased in 10% steps to mimic the gravimetric moisture (de)sorption experiments (Figure 9b). In agreement with Figure 9a, the (de)hydration reaction was recorded between 40% and 30% RH. Hydration and dehydration were observed to be slower in the RH dependent PXRD experiments than the gravimetric moisture (de)sorption measurements, which can be related to a different experimental setup.

#### 3.4.2 2,2′-Bipyridine Formic Acid Solvate

**Figure 10:**
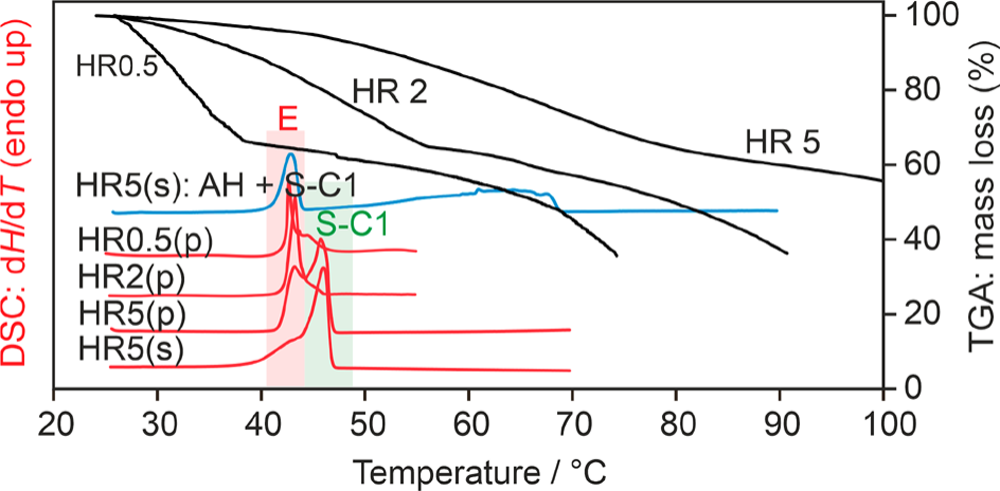
DSC and TGA thermograms of **S-C1**_**22**_ recorded at heating rates (HR) of 0.5, 2, or 5 °C min^–1^ using open (TGA), perforated (p), or sealed (s) pans. The first DSC curve corresponds to a 1:1 mixture of **S-C1**_**22**_ and **AH**_**22**_. The melting point of the solvate (S-C1) and the eutectic temperature (E) between solvate and anhydrate are highlighted.

Desolvation of the formic acid solvate starts immediately under dry conditions (N_2_ purge), as clearly evident from the 0.5 °C min^–1^ TGA heating-rate experiment (Figure 10). The mass loss corresponds to approximately 1.9 mol of formic acid per mole 2,2′-BIPY. The 5 °C min^–1^ curve shows a higher mass loss, which can be attributed to an overlap of desolvation and sublimation of the compound. The DSC heating curves of the solvate (red curves) show two peaks. The relative intensities vary depending on the heating rate and atmospheric conditions in the sample pan (hermetically sealed pans or such with perforated lids). The first endotherm, with an onset temperature at 41 °C, corresponds to the eutectic temperature between **S-C1**_**22**_ and **AH**_**22**_, as confirmed by heating a mixture of the two solid-state forms (blue thermogram—first DSC curve). The second endotherm corresponds to the melting of **S-C1**_**22**_ at 44 °C.

**Figure 11:**
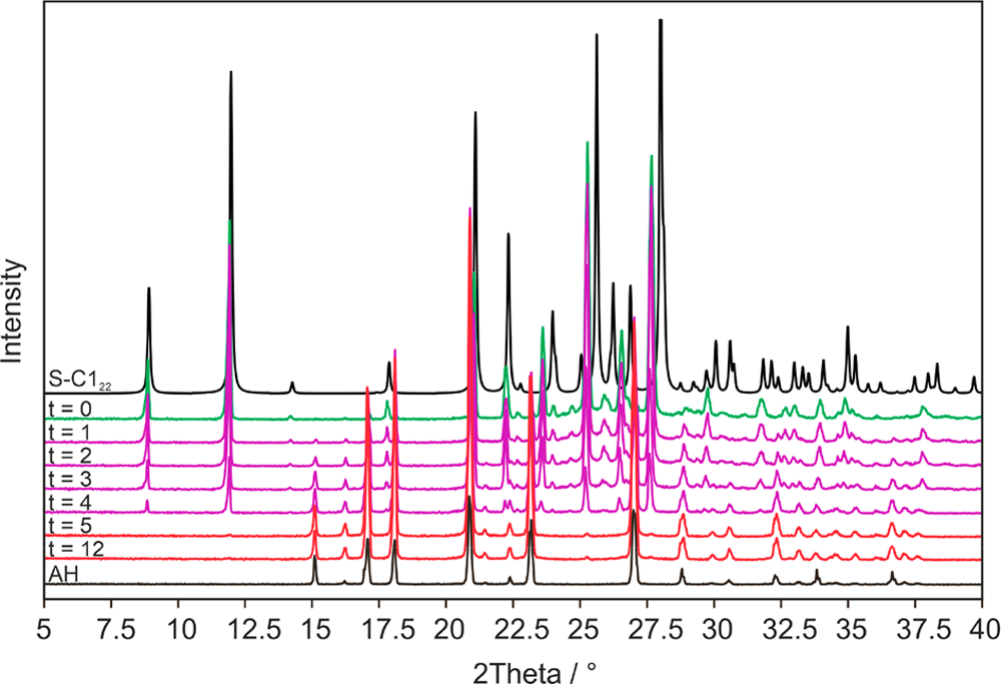
PXRD measurements monitoring the desolvation process of **S-C1**_**22**_ at ambient conditions (*t* = *x* hours). Reference patterns are provided for the anhydrate (AH) and the solvate (S-C1_22_, simulated from the single crystal structure).

The solvate was then stored at ambient conditions (25 °C and ∼30% RH). PXRD patterns were recorded in hourly intervals to monitor the stability and desolvation process of **S-C1**_**22**_ (Figure 11, a selection of the patterns is given). The first PXRD pattern already showed traces of **AH**_**22**_, which continuously increased over time. After five hours, the desolvation process was found to be completed. Therefore, it can be concluded that an unstable solvate is present. The product obtained upon desolvation corresponded to phase pure **AH**_**22**_.

#### 3.4.3 4,4′-Bipyridine Carboxylic Acid Solvates

The thermal stability of the solvates was investigated with HSM, TGA, and DSC. Furthermore, 1:1 mixtures (molar ratios) of the solvates and **AH**_**44**_, and for selected solvates mixtures of the solvate and **Hy**_**44**_, were prepared to derive the eutectic temperatures, to facilitate the interpretation of the DSC curves.

Based on the TGA results of **S-C1**_**44**_, it is evident that a disolvate is present and the solvate shows a higher thermal stability (Figure 13a) than its isomer, **S-C1**_**22**_. The fact that with a faster heating rate desolvation occurs at a higher temperature is a kinetic effect. The melting point of the solvate was determined at 98.3 °C (Table 3) using sealed DSC crucibles. The slowest heating rate applied (0.5 °C min^–1^) clearly shows the eutectic temperature between the **S-C1**_**44**_ and **AH**_**44**_ at 72 °C. Prior to that temperature, a broad low-intensity endotherm occurs (barely visible), the desolvation of the solvate to **AH**_**44**_.

**Table 3:** Compilation of Melting Data of 2,2′-BIPY and 4,4′-BIPY

solid form	*T*_fus_^a^ /°C	*T*_et_ (AH)^b^ /°C
**AH_22_**	70.3 ± 0.3	
**AH_44_**	110.9 ± <0.1	
**Hy_44_**	69.9 ± <0.1	68.5 ± 0.1
**S-C1_22_**	44.0 ± 0.3	41.1 ± 0.3
**S-C1_44_**	98.3 ± 0.4	72.0 ± 0.2
**S-C2_44-HT_**	96.0 ± 0.1	70.3 ± 0.2
**S-C3_44_**	67.3 ± 0.1	56.9 ± 0.4
**S-C4_44_**	70.7 ± 0.2	53.0 ± 0.2
**S-C5_44_**	50.1 ± 0.1	39.3 ± 0.3
**S-C6_44_**	58.3 ± 0.1	47.1 ± 0.2
**S-C8_44_**	68.4 ± 0.3	57.6 ± 0.2

a Melting point.

b Eutectic temperature.

**Figure 12:**
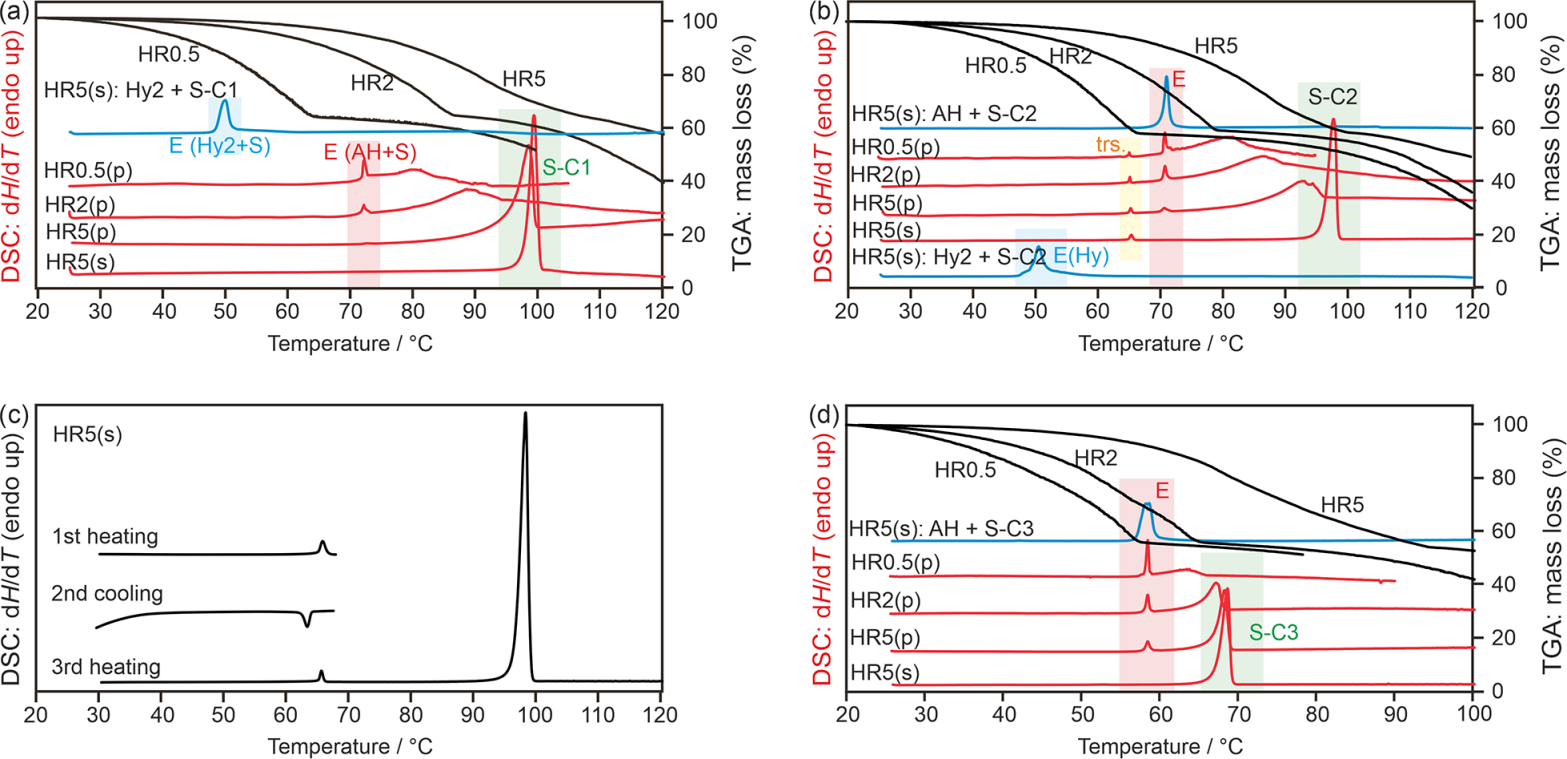
DSC and TGA thermograms of 4,4′-BIPY carboxylic acid solvates recorded at heating rates (HR) of 0.5, 2, or 5 °C min^–1^ using open (TGA), perforated (p), or sealed (s) pans. The blue curves correspond to 1:1 mixtures of the solvate and **AH**_**44**_ (or **Hy**_**44**_). Melting points of the solvates (S) and the eutectic temperatures (E) are highlighted: (a) **S-C1**_**44**_, (b,c) **S-C2**_**44**_, and (d) **S-C3**_**44**_. In (c), the reversible polymorphic phase transition of **S-C2**_**44**_ is displayed (**S-C2**_**44**_ ↔ **S-C2**_**44-HT**_).

**S-C2**_**44**_ shows an exact mass loss of 2 mol of acetic acid per mole 4,4′-BIPY (Figure 12b). The **S-C2**_**44**_ DSC curves show more than one endothermic event (red curves). Therefore, DSC curves of 1:1 mixtures of (1) **S-C2**_**44**_ and **AH**_**44**_ and (2) **S-C2**_**44**_ and **Hy**_**44**_ were measured. The closed-pan DSC measurement (5th curve) shows two endotherms; the first small endotherm occurs at 64.1 ± 0.1 °C and the second at 96.0 °C (melting of the solvate). The eutectic temperatures between **S-C2**_**44**_ and **AH**_**44**_ and **S-C2**_**44**_ and **Hy**_**44**_ occur at 70.3 °C and approximately 49 °C, respectively. Thus, neither of the two eutectic temperatures coincides with the event recorded at 64 °C. The DSC traces recorded using 5-pinholed lids all show three clearly visible events: (1) an endotherm at 64 °C, (2) the eutectic between **S-C2**_**44**_ and **AH**_**44**_, and (3) the dissolution of the solvate in the already present melt (liquidus). Furthermore, upon careful inspection of the DSC curves, a very broad endotherm below 60 °C is detectable, corresponding to the partial desolvation of **S-C2**_**44**_ to **AH**_**44**_, which is indirectly confirmed by the presence of the eutectic.

**Figure 13:**
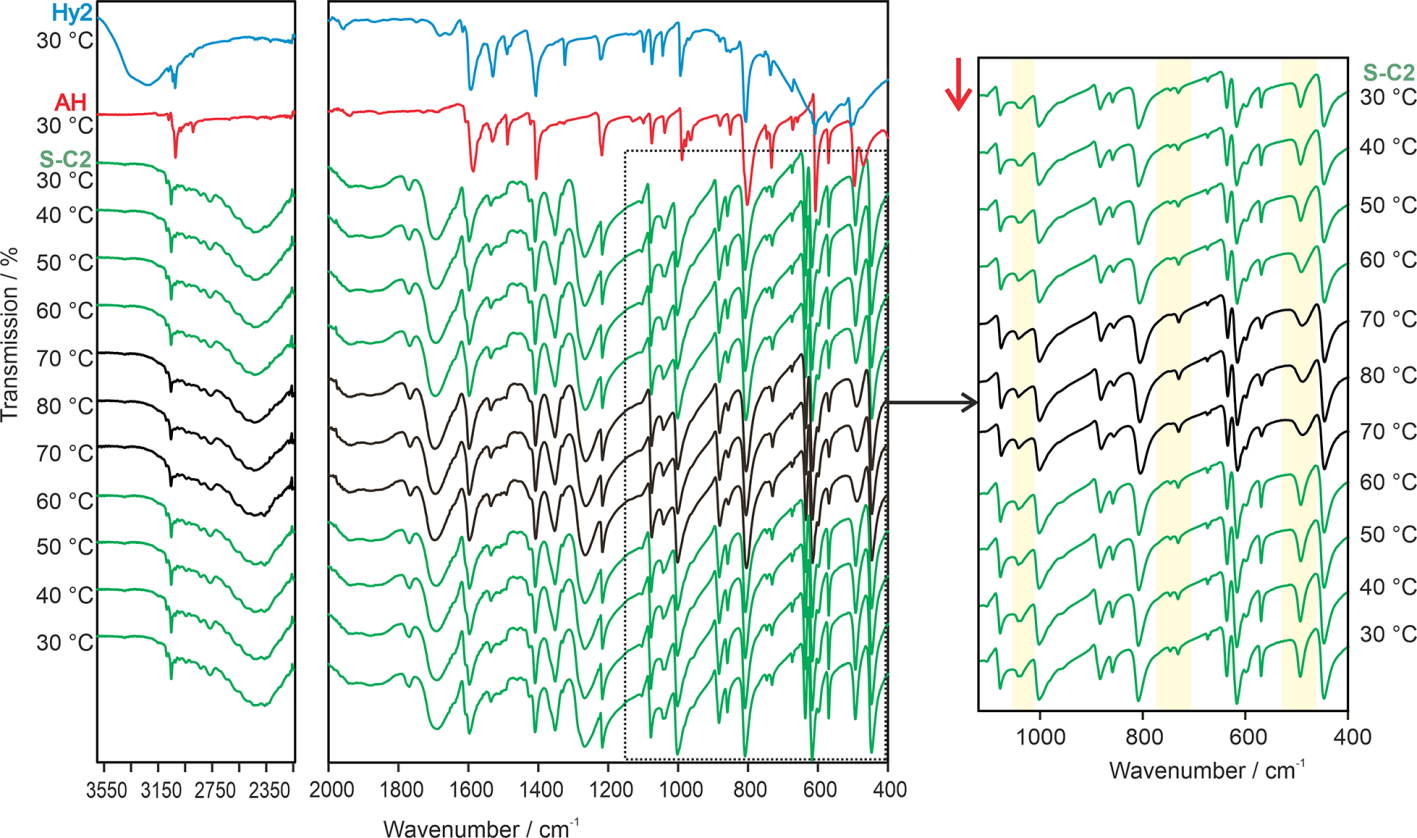
Variable temperature IR spectra of **S-C2**_**44**_ (green), contrasted to the reference spectra of **Hy**_**44**_ (blue) and **AH**_**44**_ (red). Spectral regions showing differences upon heating/cooling **S-C2**_**44**_ are highlighted on the right-hand side. Red arrow indicates the course of the experiment.

Additional DSC curves were recorded to investigate the event at 64 °C. The heating/cooling runs reveal reversibility of the process (Figure 12c), and the heats of transition were determined as 0.75 ± <0.1 kJ mol^–1^ upon heating and −0.75 ± <0.1 kJ mol^–1^ upon cooling. To support the assumption of the presence of a polymorphic phase transition (**S-C2**_**44**_ ↔ **S-C2**_**44-HT**_, with **S-C2**_**44**_ corresponding to the low/room temperature form and **S-C2**_**44-HT**_ to the high temperature form) IR heating/cooling experiments were undertaken in the relevant temperature range and contrasted to the spectra of **Hy**_**44**_ and **AH**_**44**_ (Figure 13). In the spectral range around 490, 750, and 1040 cm^–1^ subtle but reversible changes are observable upon heating to/below 70 °C, in agreement with the temperature range derived from the DSC measurements and with the presence of an enantiotropically related pair of 4,4′-BIPY acetic acid disolvates. PXRD measurements before heating and after cooling confirmed the reversibility. The second polymorph likely bears high structural similarity with **S-C2**_**44**_, indicated by the high resemblance of the IR spectra and the small enthalpy difference measured in DSC experiments.

TGA measurements of **S-C3**_**44**_ applying slower heating rates confirm a disolvate stoichiometry (Figure 12d). From the DSC experiments, it is possible to derive the melting point of **S-C3**_**44**_ at 67.3 °C and the eutectic between the solvate and **AH**_**44**_ at 56.9 °C (Table 3). The thermo-analytical measurements of the solvates **S-C1**_**44**_, **S-C2**_**44**_, and **S-C3**_**44**_ reveal that only by applying the slowest heating rates and conditions as in the TGA is it possible to desolvate the solvates prior to the eutectic and solvate melting temperatures.

**Figure 14:**
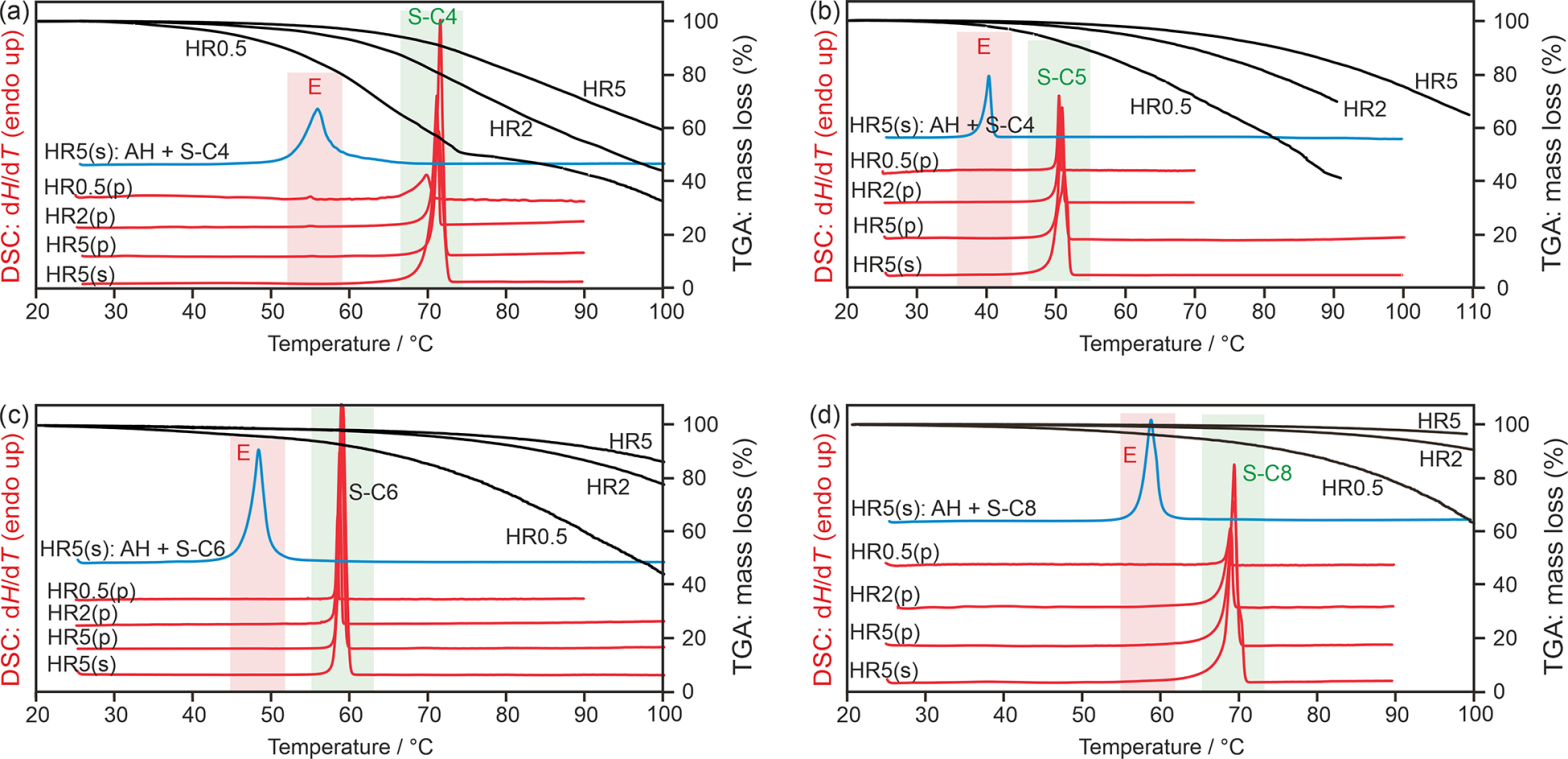
DSC and TGA thermograms of 4,4′-BIPY carboxylic acid solvates recorded at heating rates (HR) of 0.5, 2, or 5 °C min^–1^ using open (TGA), perforated (p), or sealed (s) pans. The blue curves correspond to 1:1 mixtures of the solvate and **AH**_**44**_. Melting points of the solvates (S) and eutectic temperatures (E) are highlighted: (a) **S-C4**_**44**_, (b) **S-C5**_**44**_, (c) **S-C6**_**44**_, and (d) **S-C8**_**44**_.

The TGA and DSC results of the remaining four 4,4′-BIPY solvates are displayed in Figure 14. The melting point of the solvates were derived as follows: **S-C4**_**44**_: 70.7 °C, **S-C5**_**44**_: 50.1 °C, **S-C6**_**44**_: 58.3 °C, and **S-C8**_**44**_: 68.4 °C (Table 3). The stoichiometry could only be determined for **S-C4**_**44**_ (heating rate 0.5 °C min^–1^). For the three other solvates (**S-C5**_**44**_, **S-C6**_**44**_, and **S-C8**_**44**_), desolvation starts at a higher temperature and overlaps with the melting temperature of the solvates and sublimation of the compound.

The slow heating-rate DSC measurements of **S-C4**_**44**_ show the eutectic temperature between the solvate and **AH**_**44**_ (Figure 14a) at 56.9 °C. For **S-C5**_**44**_ (Figure 14b), **S-C6**_**44**_ (Figure 14c), and **S-C8**_**44**_ (Figure 14d), independent of the used heating rate and DSC pans, only the melting point of the solvates could be determined. The eutectic temperatures were additionally measured using 1:1 mixtures. The melting points and eutectic temperatures (Table 3) reveal that **S-C1**_**44**_ and **S-C2**_**44**_ are the highest melting solvates (≥96 °C) of the 4,4′-BIPY solvates, followed by **S-C4**_**44**_, **S-C8**_**44**_, and **S-C3**_**44**_ (approximately 70 °C), then **S-C6**_**44**_ and finally **S-C5**_**44**_ (50 °C). Thus, the monoclinic solvates show higher melting points than the triclinic solvates, and within the group of the triclinic solvates **S-C5**_**44**_, the only structure with a bent acid conformation, shows the lowest melting point.

The thermo-analytical measurements of the 4,4′-BIPY solvates were complemented with storage stability experiments at ambient conditions (RT, 30–40% RH), monitored first hourly and then daily with PXRD (Supporting Information, section 9). Within a few days, **S-C1**_**44**_, **S-C2**_**44**_, and **S-C3**_**44**_ desolvated to either **Hy2**_**44**_ or **AH**_**44**_, depending on the moisture conditions present. Upon further storing the compounds at room conditions, transitions between the **Hy2**_**44**_ and **AH**_**44**_ were observed, highlighting the problematic phase control of 4,4′-BIPY if handled at not very precisely controlled RH conditions. At ambient conditions, the desolvation of **S-C4**_**44**_ and **S-C5**_**44**_ took approximately twice as long as for **S-C1**_**44**_, **S-C2**_**44**_, and **S-C3**_**44**_ (<9–10 days as opposed to <3–5 days) and resulted again in either **AH**_**44**_ or **Hy2**_**44**_. The desolvation process of **S-C6**_**44**_ and **S-C8**_**44**_ proceeded very slowly. In the case of **S-C6**_**44**_, the solvate was still detectable after 6 weeks, and no desolvation was visible for **S-C8**_**44**_ after 12 weeks (end of the investigation time). To conclude, with an increase in the chain length of the acid the kinetic stability of the solvate at ambient conditions increases.

### 3.5 Energy Comparisons of Single and Multicomponent Forms

The direct energy comparison (enthalpy) of anhydrates and different solvates is not straightforward. Therefore, computational methods were employed, and in the case of the **AH**_**44**_ ↔ **Hy2**_**44**_, the calculated values are contrasted with calorimetric measurements.

#### 3.5.1 Lattice Energy Comparison of 2,2′-BIPY and 4,4′-BIPY Solid-State Forms

Lattice energy calculations were performed to estimate whether the solvate structures are more stable than the anhydrates, i.e., whether solvate formation is thermodynamically driven. Only the main disorder components of the disordered solvate structures were considered. To be able to compare the lattice energies between the different disolvates (including the dihydrate) and anhydrates, the lattice energies of the solvates were corrected by the energetic cost of removing the solvent molecules and an energy correction term equal to 3/2 *RT* at 300 K, as described in section 2.4. Table 4 lists the energy comparison for BIPY solvates, the solvent crystal structures, and solvent corrected solvate structures (*E*_comp_^tot^*)*.

**Table 4:** Lattice Energy Comparisons for BIPY Solvates (*E*_solvate_^tot^), Solvent Crystal Structures (*E*_solvent_^tot^), Anhydrates, and Solvent Corrected Solvate Structures (*E*_comp_^tot^)

solvate	*E*_solvate_^tot^ /kJ mol^–1^		*E*_solvent_^tot^ /kJ mol^–1^		*E*_comp_^tot^ /kJ mol^–1^^a^	
	PBE-TS	PBE-MBD*	PBE-TS	PBE-MBD*	PBE-TS	PBE-MBD*
2,2′-BIPY						
**AH_22_**					–137.04^b^	–96.21^b^
**S-C1_22_**	–307.70	–259.65	–74.48	–70.77	–155.00	–114.37
4,4′-BIPY						
**AH_44_**					–143.07^b^	–102.45^b^
**Hy_44_**	–293.24	–252.42	–68.62	–68.41	–152.26	–111.86
**S-C1_44_**	–326.88	–281.39	–74.48	–70.77	–174.18	–136.11
**S-C2_44_**	–343.36	–287.57	–86.79	–79.39	–166.04	–125.05
**S-C3_44_**	–357.30	–291.69	–98.16	–85.11	–157.24	–117.73
**S-C4_44_**	–389.17	–311.21	–110.17	–91.50	–165.09	–124.47
**S-C5_44_**	–412.95	–324.45	–125.28	–98.93	–158.65	–122.85
**S-C6_44_**	–438.47	–338.15	–137.03	–106.33	–160.67	–121.75
**S-C8_44_**	–510.87	–383.98	–169.13	–125.29	–168.87	–129.66

a *E*_comp_^tot^ calculated according to eq 1.

b Anhydrate lattice energy.

An *E*_comp_^tot^ value, which is lower (more stable) than the lattice energy of the corresponding anhydrate, indicates that the second component present in the solvate (hydrate) structure stabilizes the multicomponent system and rationalizes its formation. In the case of the nine experimental solvates (including the hydrate), the calculations provide quantitative values, confirming that the formation of the additional strong H-bonding interactions between BIPY and acid or water molecules is very favorable. The latter can be related to the well-known driving force for solvate/cocrystal formation, an imbalance in H-bond donor and acceptor groups (BIPY has no donor group).

The direct comparison of the *E*_comp_^tot^ for the formic acid solvates **S-C1**_**22**_ and **S-C1**_**44**_ reveals that the inclusion of formic acid into the crystal lattice adds more to the stability of the 4,4′-BIPY solvate than its 2,2′- isomer. This observation correlates well with the stability data derived from the TGA and DSC measurements at ambient conditions, which indicated a lower stability of **S-C1**_**22**_ than **S-C1**_**44**_.

Considering only the series of 4,4′-BIPY solvates, no clear trends can be derived from the calculations. **S-C1**_**44**_ is calculated as the most stable, followed by **S-C8**_**44**_ and **S-C2**_**44**_, although with a considerable energy gap (Table 4). Interestingly, one of the smallest and the biggest considered acid solvate structure were identified as equally stable. Thus, not only the strong H-bonding interaction but also the hydrocarbon close contacts (more in **S-C8**_**44**_ than **S-C2**_**44**_) contribute to the stability of the structure. **S-C3**_**44**_ was estimated as the least stable solvate, although **S-C3**_**44**_, **S-C4**_**44**_, and **S-C6**_**44**_ show disorder, and this will entropically stabilize the structures (not considered in the calculations).

#### 3.5.2 Enthalpy of Dehydration

As already shown for other hydrate systems,^81,82^ it is possible to estimate the transition enthalpy of hydrate ↔ anhydrate systems from calorimetric measurements and lattice energy calculations. In this study, the dehydration energy was derived with isothermal calorimetry using an RH perfusion cell. The heat of transition was calculated by subtracting the heat of condensation of 2 mol of water (−43.99 kJ mol^–1^, at 25 °C)^83^ from the measured heat of hydration (Δ_hy_*H*_AH-Hy2_ = −106.0 ± 1.4 kJ mol^–1^) as −14.4 ± 1.4 kJ mol^–1^, a value slightly lower than measured for other dihyrate systems.^14,81,82^ Overall, **Hy2**_**44**_ shows a considerable energy difference from **AH**_**44**_, despite being a channel hydrate (Figure 5b). The latter fact explains the fast dehydration kinetics, as the water channels facilitate the water release, and the empty channels provide space for the 90° turns of every alternate stack of 4,4′-BIPY molecules (Figure 5a). The driving force for hydration can clearly be attributed, similarly to the carboxylic acid solvate formation, to the formation of strong H-bonding interactions, as evident by the intermolecular interaction energy calculations and the energy gain of approximately −14 kJ mol^–1^ (heat of transition).

Using the lattice energy values calculated in Table 4, i.e., subtracting from the *E*_comp_^tot^ value of **Hy2**_**44**_ the lattice energy of **AH**_**44**_, the potential energy difference between **Hy2**_**44**_ and **AH**_**44**_ is obtained. The energy difference of 9.2 (PBE-TS) and 9.4 kJ mol^–1^ (PBE-MBD*), with **Hy2**_**44**_ being more stable, is roughly the magnitude of the experimental value (Δ_hy_*H* ∼ −Δ*E*_latt_) and confirms hydrate formation of the compound.

## 4 Conclusions

The two investigated compounds, 2,2′-BIPY and 4,4′-BIPY, which are intensively used in coordination chemistry and also as coformers, show a different tendency toward hydrate and solvate formation with carboxylic acids. 2,2′-BIPY was found to form a solvate with formic acid only, whereas for the 4,4′-isomer, solvates were identified for all investigated carboxylic acids (formic, acetic, propionic, butyric, valeric, caproic, and caprylic). For 4,4′-BIPY, the existence of a polymorphic solvate could be shown. The anhydrates and formic acid solvates of the two isomers are structurally distinct. Thus, the position of the N defines the packing arrangements. The terminal location of the N atoms in the 4,4′-isomer (no sterical hindrance) facilitates the accommodation of bigger carboxylic acids and also a high conformational flexibility. The tendency toward the formation of multicomponent crystal forms with carboxylic acids agrees with the high number of CSD cocrystals, and the lack of H-bonding donor groups of the bipyridines has to be seen as the driving force for the formation of multicomponent solid-state forms. This conclusion is backed up by the high energy contribution (−46.2 to −56.4 kJ mol^–1^ in pairwise energy) of the strong O–H···N H-bonds. Furthermore, the lattice energy calculations clearly indicate a thermodynamic driving force for solvate formation.

Hydrate formation was only seen for 4,4′-BIPY, with the critical water activity being at ambient conditions (0.35 at RT), complicating storage and handling, especially weighting operations, of the compound. Surprisingly, this information, crucial for the synthesis of any metal complex or cocrystal, has not been reported or addressed in the literature so far. The fast and facile dehydration mechanism can be rationalized based on the location of the water molecules in open channels and the hydration by the energy gain observed upon the inclusion of the water molecules.

The CSP studies revealed differences in the two compounds. More packing diversity was seen for 2,2′-BIPY, which has the easily crystallizing anhydrate as the global minimum structure. This is in contrast to 4,4′-BIPY, where structurally related packings were found to be lower in lattice energy than the experimental form, either indicating that more stable not yet discovered polymorph(s) of 4,4′-BIPY exist or reflecting the limitations of the thermodynamic modeling accuracy.^78^

## Supporting Information Available

Experimental solid form screen; potential energy surface scans; crystal structure prediction results; representation of the experimental forms; structure comparisons; substitution calculations; morphologies of the solvate crystals; crystallographic information; PXRD, IR, and moisture (de)sorption data; stability experiments at ambient conditions (PDF)

## Accession Codes

CCDC 2108062–2108066 contain the supplementary crystallographic data for this paper. These data can be obtained free of charge via www.ccdc.cam.ac.uk/data_request/cif, or by emailing data_request@ccdc.cam.ac.uk, or by contacting The Cambridge Crystallographic Data Centre, 12 Union Road, Cambridge CB2 1EZ, UK; fax: +44 1223 336033.
