# Supplementary material for: Expanding the Solid Form Landscape of Bipyridines
Source: Cryst Growth Des. 2021 Nov 10;21(12):7201–17. doi: 10.1021/acs.cgd.1c01045 (PMC8640990; doi:10.1021/acs.cgd.1c01045)
Supplement: Supplementary file 1 — cg1c01045_si_001.pdf [file cg1c01045_si_001.pdf]

Supplementary Information

Expanding the solid form landscape of Bipyridines

Doris E. Braun,[†,] Patricia Hald,[†] Volker Kahlenberg[‡] and Ulrich J. Griesser[†]*

[†]Institute of Pharmacy, University of Innsbruck, Innrain 52c, 6020 Innsbruck, Austria

[‡]Institute of Mineralogy and Petrography, University of Innsbruck, Innrain 52, 6020 Innsbruck, Austria

*Email: doris.braun@uibk.ac.at

Table of Contents

1. **Experimental solid form screen** 3
2. **Potential energy surface scans (PES) of the carboxylic acids** 6
3. **Representation of the experimental structures and crystal structure prediction** 10
4. **Substitution calculations** 21
5. **Morphology of the solvates** 23
6. **Crystallographic information** 26
7. **2,2’-BIPY and 4,4’-BIPY Anhydrates and Hydrate** 33
8. **Solvates of 2,2’-BIPY and 4,4’-BIPY** 35
9. **Stability at ambient conditions** 37

References 41

1. Experimental solid form screen

1.1. Evaporation experiments

Fast evaporation experiments were performed by dissolving 20 mg of the anhydrate in 0.1 mL of solvent. In case the compound dissolved in the used amount the solution was directly transferred onto a watch-glass, otherwise solvent was added in 0.1 mL steps under stirring until a clear solution was obtained and the solution filtered and transferred onto the watch-glass and covered with a filter paper. The crystallization product was characterized using polarized microscopy and powder X-ray diffractometry. Few of the crystallization products showed a transformation, as indicated in Table S1 (2,2'-BIPY) and Table S2 (4,4'-BIPY).

For 2,2'-BIPY only the in the literature described anhydrate (**AH_{22}**) was obtained according to the PXRD data. However, in case of the formic acid evaporation experiment a microscopic observation of the particles confirmed that the initially formed solvate **S-$C1_{22}$** desolvated already to the anhydrate **AH_{22}**.

Table S1. Evaporation screen of **2,2'-BIPY.**

Solvent (mL)	Solid-state form	Solvent (mL)	Solid-state form
Water (3.3)	AH_{22}	Butyl acetate (0.1)	AH_{22}
Methanol (0.1)	AH_{22}	Dimethyl carbonate (0.1)	AH_{22}
Ethanol (0.1)	AH_{22}	Acetonitrile (0.1)	AH_{22}
n-Propanol (0.1)	AH_{22}	Nitromethane (0.1)	AH_{22}
i-Propanol (0.1)	AH_{22}	Dimethyl formamide (0.1)	AH_{22}
n-Butanol (0.1)	AH_{22}	Dimethyl acetamide (0.1)	AH_{22}
t-Butanol (0.2)	AH_{22}	Dimethyl sulfoxide (0.1)	AH_{22}
n-Octanol (0.1)	AH_{22}	Dichloroethane (0.1)	AH_{22}
Ethylene glycol (0.4)	AH_{22}	Chloroform (0.1)	AH_{22}
Tetrahydrofuran (0.1)	AH_{22}	Cyclohexane (0.1)	AH_{22}
1,4-Dioxane (0.1)	AH_{22}	n-Heptane (0.4)	AH_{22}
Diethyl ether (0.1)	AH_{22}	Toluene (0.1)	AH_{22}
Di-isopropyl ether (0.2)	AH_{22}	Xylene (0.1)	AH_{22}
Methyl-t-butyl ether (0.1)	AH_{22}	Formic acid (C1) (0.1)	AH_{22}
Acetone (0.1)	AH_{22}	Acetic acid (C2) (0.1)	S-$C1_{22}$ → AH_{22}[a]
Methyl ethyl ketone (0.1)	AH_{22}	Propionic acid (C3) (0.1)	AH_{22}
Methyl isobutyl ketone (0.1)	AH_{22}	Butyric acid (C4) (0.1)	AH_{22}
Cyclohexanone (0.1)	AH_{22}	Valeric acid (C5) (0.1)	AH_{22}
Ethyl acetate (0.1)	AH_{22}	Caproic acid (C6) (0.1)	AH_{22}
i-Butyl acetate (0.1)	AH_{22}	Caprylic acid (C8) (0.1)	AH_{22}

[a]Pseudomorphosis

The majority of the 4,4'-BIPY evaporation experiments resulted in the known anhydrate form (**AH_{44}**). The evaporation experiment from water resulted in needle-shaped particles with a pronounced secondary structure due to the dehydration after removal of the mother liquor. Similar needle-shaped "pseudomorphs" were also seen together with anhydrate crystals in case of the butyl acetate, nitromethane and dichloroethane evaporation experiments, indicating that concomitant crystallization of **AH_{44}** and **$Hy2_{44}$** had occurred. Evaporation experiments from formic acid (immediately measured), caproic acid and caprylic acid showed each distinct PXRD patterns. Crystals obtained from acetic acid, propionic acid, butyric acid and valeric acid were mixtures of new forms and **AH_{44}**. Thus, nine solid-state forms were identified in the 4,4'-BIPY RT watch-glass evaporation experiments.

Table S2. Evaporation screen of **4,4'-BIPY.**

Solvent (mL)	Solid-state form	Solvent (mL)	Solid-state form
Water (4.2)	$Hy2_{44}$ → AH_{44} [a]	Butyl acetate (0.1)	$Hy2_{44}$ → AH_{44}[a] + AH_{44}
Methanol (0.1)	AH_{44}	Dimethyl carbonate (0.2)	AH_{44}
Ethanol (0.1)	AH_{44}	Acetonitrile (0.1)	AH_{44}
n-Propanol (0.1)	AH_{44}	Nitromethane (0.1)	$Hy2_{44}$ → AH_{44} [a] + AH_{44}
i-Propanol (0.1)	AH_{44}	Dimethyl formamide (0.1)	AH_{44}
n-Butanol (0.1)	AH_{44}	Dimethyl acetamide (0.1)	AH_{44}
t-Butanol (0.3)	AH_{44}	Dimethyl sulfoxide (0.1)	AH_{44}
n-Octanol (0.3)	[b]	Dichloroethane (0.1)	$Hy2_{44}$ → AH_{44}[a] + AH_{44}
Ethylene glycol (0.2)	[b]	Chloroform (0.1)	AH_{44}
Tetrahydrofuran (0.1)	AH_{44}	Cyclohexane (>4.2)	AH_{44}
1,4-Dioxane (0.1)	AH_{44}	n-Heptane (4.0)	AH_{44}
Diethyl ether (0.8)	AH_{44}	Toluene (0.9)	AH_{44}
Di-isopropyl ether (2.7)	AH_{44}	Xylene (0.1)	AH_{44}
Methyl-t-butyl ether (0.7)	AH_{44}	Formic acid (C1) (0.1)	$S\text{-}C1_{44}$
Acetone (0.1)	AH_{44}	Acetic acid (C2) (0.4)	$S\text{-}C2_{44}$ → AH_{44} [c]
Methyl ethyl ketone (0.1)	AH_{44}	Propionic acid (C3) (0.2)	$S\text{-}C3_{44}$ → AH_{44} [c]
Methyl isobutyl ketone (0.1)	AH_{44}	Butyric acid (C4) (0.4)	$S\text{-}C4_{44}$ → AH_{44} [c]
Cyclohexanone (>9.5)	AH_{44}	Valeric acid (C5) (0.2)	$S\text{-}C5_{44}$ → AH_{44} [c]
Ethyl acetate (0.2)	AH_{44}	Caproic acid (C6) (0.4)	$S\text{-}C6_{44}$
i-Butyl acetate (0.3)	AH_{44}	Caprylic acid (C8) (>2,5)	$S\text{-}C8_{44}$

[a]only needle shaped crystals showed pseudomorphosis; [b]no crystallization occurred within 3 months; [c]pseudomorphosis.

1.2. Slurry experiments in water and selected organic solvents

Slurry experiments in eleven organic solvents, chosen based on the watch-glass evaporation experiments, were performed in the temperature range between 10 and 30 °C (cycling). Samples were withdrawn periodically and analysed "wet" using PXRD. All literature forms

were confirmed and six new solvates found (one for 2,2'-BIPY and five for 4,4'-BIPY). The results are given in Table S3.

Table S3. **Slurry screen of** 2,2'-BIPY **and** 4,4'-BIPY.

Solvent	Solid-state form (2,2'-BIPY)	Solid-state form (4,4'-BIPY)
Water	AH_{22}	$Hy2_{44}$
Butyl acetate	AH_{22}	AH_{44}
Nitromethane	AH_{22}	AH_{44}
Dichloroethane	AH_{22}	AH_{44}
Formic acid (C1)	$S\text{-}C1_{22}$	$S\text{-}C1_{44}$
Acetic acid (C2)	AH_{22}	$S\text{-}C2_{44}$
Propionic acid (C3)	AH_{22}	$S\text{-}C3_{44}$
Butyric acid (C4)	AH_{22}	$S\text{-}C4_{44}$
Valeric acid (C5)	AH_{22}	$S\text{-}C5_{44}$
Caproic acid (C6)	AH_{22}	$S\text{-}C6_{44}$
Caprylic acid (C8)	AH_{22}	$S\text{-}C8_{44}$

1.3. Cooling crystallization experiments

The cooling crystallization screen was designed from eleven solvents that were selected based on the results of the watch-glass evaporation experiments. Hot saturated solutions of 50 – 75 mg of the compounds were prepared and then crystallization was induced by natural cooling to RT. In case of the 2,2'- isomer only the formic acid experiment resulted in a solid-state form different from the anhydrate (**AH_{22}**). For 4,4'-BIPY the limited cooling crystallization screen resulted in the hydrate (**$Hy2_{44}$**), anhydrate (**AH_{44}**) and seven different carboxylic acid solvates (Table S4).

Table S4. Summary of cooling crystallization experiments

Solvent	Solid-state form (2,2'-BIPY)	Solid-state form (4,4'-BIPY)
Water	AH_{22}	$Hy2_{44}$
Butyl acetate	AH_{22}	AH_{44}
Nitromethane	AH_{22}	AH_{44}
Dichloroethane	AH_{22}	AH_{44}
Formic acid (C1)	$S\text{-}C1_{22}$	$S\text{-}C1_{44}$
Acetic acid (C2)	AH_{22}	$S\text{-}C2_{44}$
Propionic acid (C3)	AH_{22}	$S\text{-}C3_{44}$
Butyric acid (C4)	AH_{22}	$S\text{-}C4_{44}$
Valeric acid (C5)	AH_{22}	$S\text{-}C5_{44}$
Caproic acid (C6)	AH_{22}	$S\text{-}C6_{44}$
Caprylic acid (C8)	AH_{22}	$S\text{-}C8_{44}$

2. Potential energy surface scans (PES) of the carboxylic acids

PES scans were performed at the B3LYP/6-31G(d,p) level of theory using GAUSSIAN09.[1] The dihedral angels marked in Figure S1 were scanned in 20° (1-dimensional scans) or 30° (2-dimensional) scans. Dihedral angles not scanned were optimized (extended conformation of the acid).

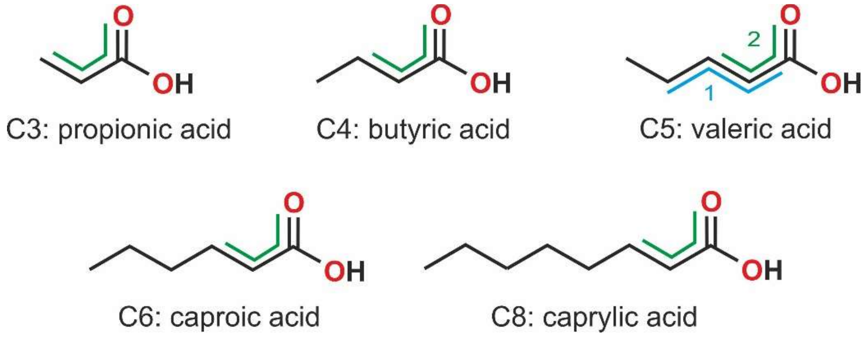

Figure S1. Carboxylic acids with the for the analyses selected dihedral angles marked in green and blue (C5 only).

2.1. Propionic acid (C3)

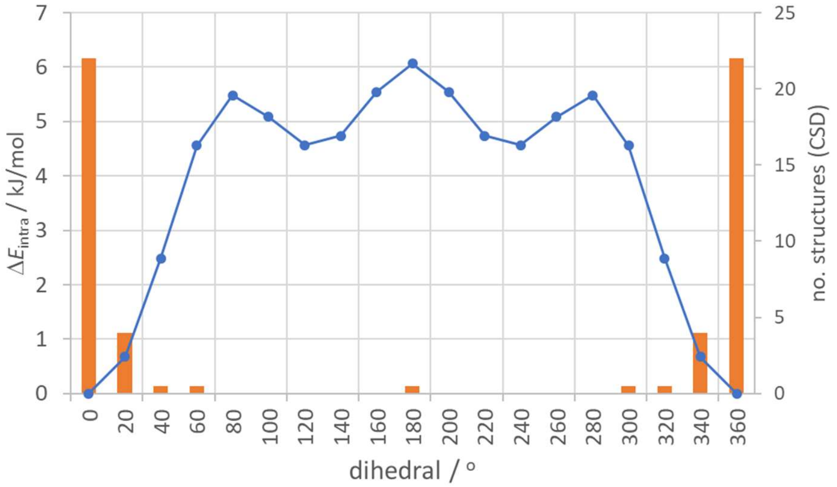

Figure S2. Potential energy surface scan of propionic acid performed at the B3LYP/6-31G(d,p) level of theory and number of conformers found in the CSD (orange bars). Note that due to the symmetry of acid molecule most conformers are present twice in the 360° scan. Conformers were distributed uniformly, i.e. each CSD conformer is counted only once.

2.2. Butyric Acid (C4)

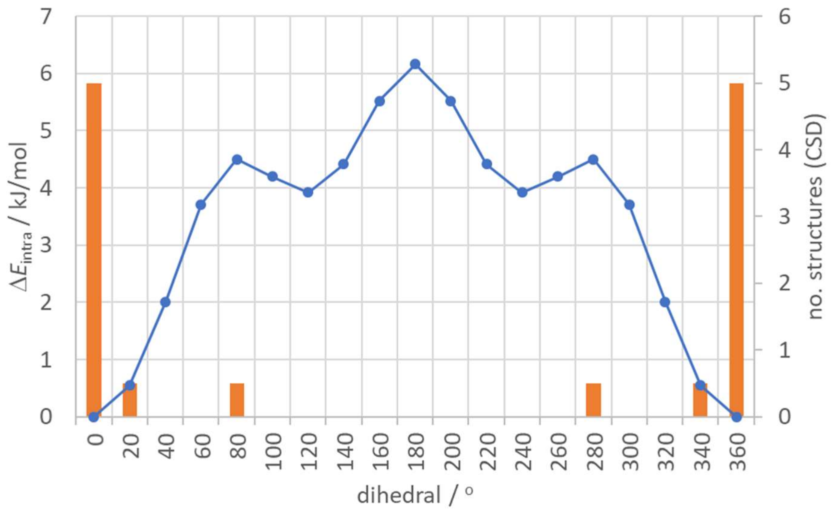

Figure S3. Potential energy surface scan of butyric acid performed at the B3LYP/6-31G(d,p) level of theory and number of conformers found in the CSD (orange bars). Note that due to the symmetry of acid molecule most conformers are present twice in the 360° scan. Conformers were distributed uniformly, i.e. each CSD conformer is counted only once.

2.3. Valeric acid (C5)

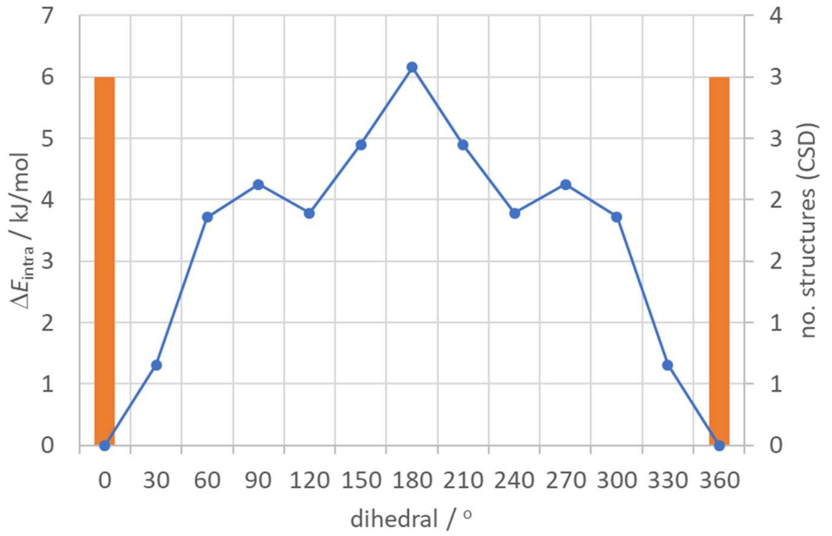

Figure S4. Potential energy surface scan of valeric acid performed at the B3LYP/6-31G(d,p) level of theory and number of conformers found in the CSD (orange bars). Note that due to the symmetry of acid molecule most conformers are present twice in the 360° scan. Conformers were distributed uniformly, i.e. each CSD conformer is counted only once.

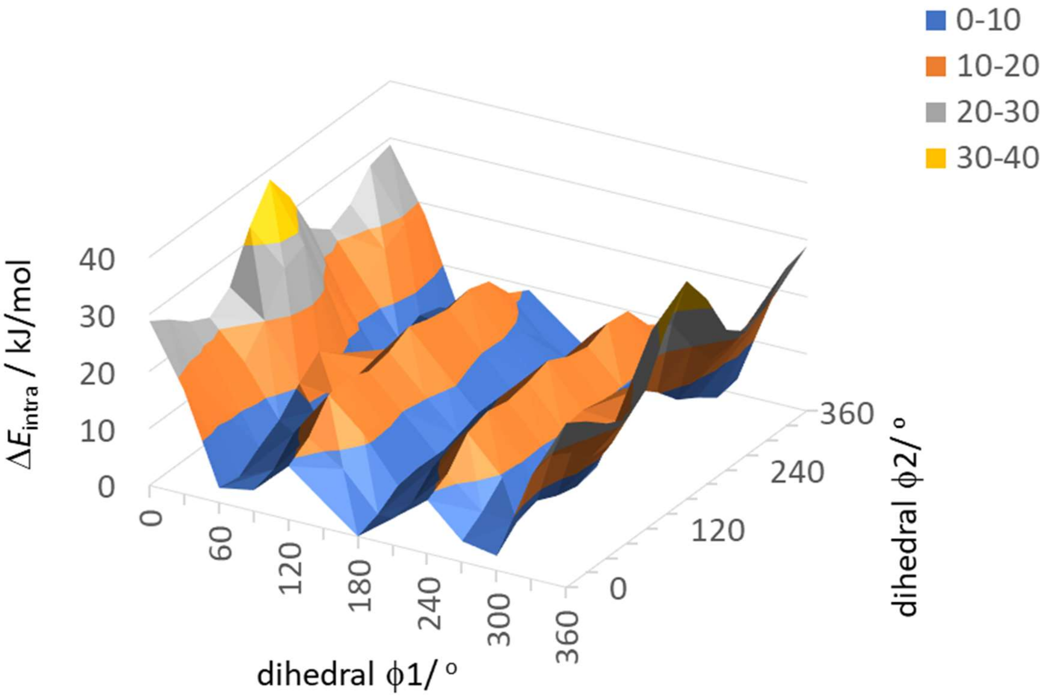

Figure S5. 2D-Potential energy surface scan of valeric acid performed at the B3LYP/6-31G(d,p) level of theory, color coded according to intramolecular energy difference with respect to the global minimum conformation (in kJ mol^{-1}).

2.4. Caproic acid (C6)

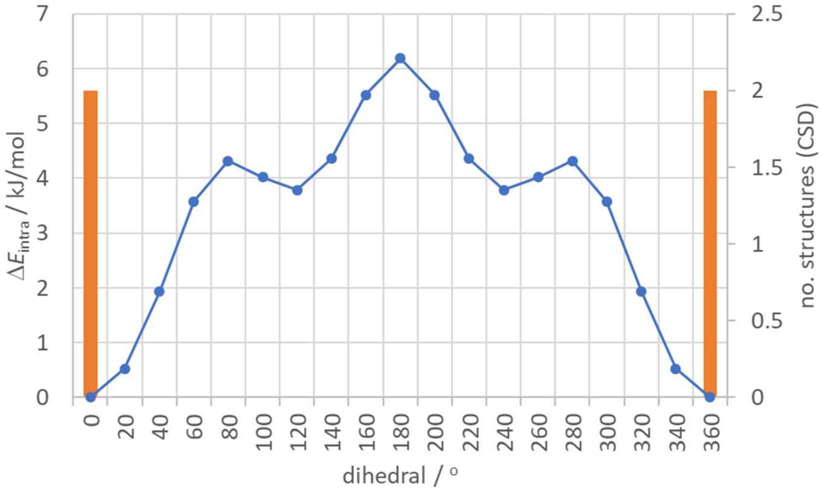

Figure S6. Potential energy surface scan of caproic acid performed at the B3LYP/6-31G(d,p) level of theory and number of conformers found in the CSD (orange bars). Note that due to the symmetry of acid molecule most conformers are present twice in the 360° scan. Conformers were distributed uniformly, i.e. each CSD conformer is counted only once.

2.5. Caprylic acid (C8)

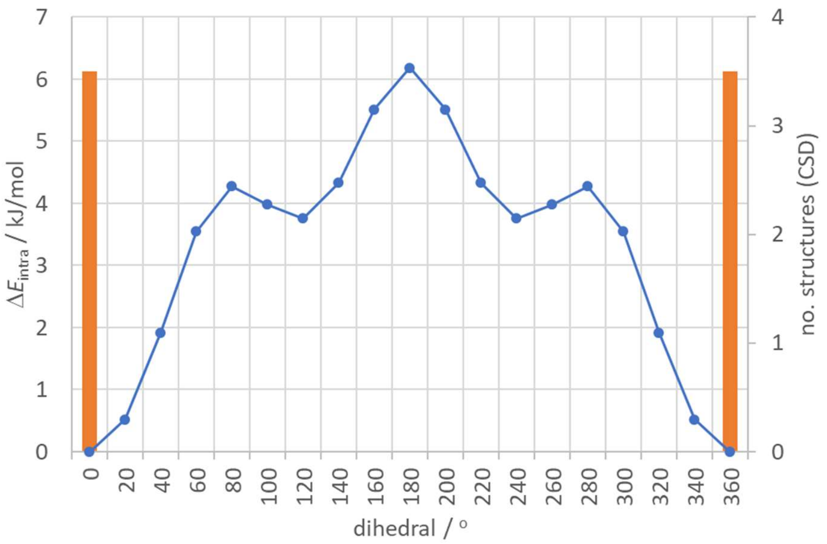

Figure S7. Potential energy surface scans of caprylic acid performed at the B3LYP/6-31G(d,p) level of theory and number of conformers found in the CSD (orange bars). Note that due to the symmetry of acid molecule most conformers are present twice in the 360° scan. Conformers were distributed uniformly, i.e. each CSD conformer is counted only once.

3. Representation of the experimental structures and crystal structure prediction

3.1. Representation of the experimental forms

The computational models were successful in reproducing the experimental structures (Table S5). The structures were compared using the Molecular Similarity Module in Mercury to determine the root mean square deviation of the non-hydrogen atoms in a cluster of 15 molecules ($rmsd_{15}$).[2]

Table S5. Quality of the representation of the experimental structures of 2,2'-BIPY and 4,4'-BIPY.

Solid Form	Lattice parameters (cell vectors/Å, angles/°)						$rmsd_{15}$ (Å)
	a / Å	b / Å	c / Å	α / °	β / °	γ / °	
2,2'-Bipyridine							
*Anhydrate (**AH_{22}**)*							
BIPYRL, $P2_1/c$, RT	5.660	6.240	13.460	90	118.73	90	
BIPYRL01, $P2_1/c$, RT	5.069	6.189	13.400	90	118.80	90	
BIPYRL02, $P2_1/n$, RT	11.920	6.240	5.510	90	96.40	90	
BIPYRL03, $P2_1/c$, 110 K	5.485(1)	6.177(1)	12.356(4)	90	110.83(1)	90	
BIPYRL04, $P2_1/n$, 123 K	5.486(<1)	6.166(<1)	11.609(<1)	90	95.28(<1)	90	
CryOpt, Pc ($P2_1/c$), 0 K	5.655	6.280	13.407	90	120.84	90	0.16
PBE-TS, Pc ($P2_1/c$), 0 K	5.514	6.157	11.378	90	96.69	90	0.12
*Formic Acid Solvate (**S-$C1_{22}$**)*							
S-$C1_{22}$, $Pbc2_1$, 173 K	3.709(<1)	15.9139(<1)	19.832(<1)	90	90	90	
CryOpt, $Pbc2_1$, 0 K	3.659	16.226	19.826	90	90	90	0.14
PBE-TS, $Pca2_1$, 0 K	15.888	3.633	19.822	90	90	90	0.05
4,4'-Bipyridine							
*Anhydrate (**AH_{44}**)*							
HIQWEJ, P-1, 203 K	8.778 (2)	8.781(1)	10.998(2)	85.52(1)	85.42(2)	75.57(1)	
HIQWEJ01, P-1, RT	8.830(2)	8.894(1)	11.024(2)	85.48(2)	85.41(1)	77.83(3)	
HIQWEJ02, P-1, 193 K	8.761(<1)	8.779(<1)	11.011(<1)	85.26(<1)	85.40(<1)	78.71(<1)	
HIQWEJ03, P-1, 150 K	8.693(<1)	8.735(<1)	10.982(<1)	85.14(<1)	85.37(<1)	78.95(<1)	
CryOpt, Pc, P-1, 0 K	9.003	8.760	11.072	92.77	93.45	78.40	0.20
PBE-TS, Pc, P-1, 0 K	8.450	8.792	10.920	85.79	85.81	79.66	0.15
*Dihydrate (**$Hy2_{44}$**)*							
WOVYEL, $P2_1$, 130 K	9.133 (<1)	7.431(<1)	14.717(1)	90	101.05(<1)	90	
WOVYEL01, $P2_1/n$, RT	9.249(1)	7.583(1)	14.816(3)	90	100.75(3)	90	
WOVYEL02, $P2_1$, 93 K	9.121(<1)	7.410(<1)	14.721(<1)	90	100.99(<1)	90	
WOVYEL03, $C2$, 120 K	15.816(4)	3.713(<1)	9.177(2)	90	113.71(1)	90	
CryOpt, $P2_1$, 0 K	9.504	7.254	14.466	90	102.92	90	0.26
PBE-TS, $P2_1$, 0 K	9.068	7.275	14.621	90	101.15	90	0.06
*Formic Acid Solvate (**S-$C1_{44}$**)*							
GOKCEQ, $P2_1$, RT	3.786(5)	7.938(10)	20.940(30)	90	95.16(3)	90	
CryOpt, $P2_1$, 0 K	3.640	7.676	22.339	90	96.79	90	0.36
PBE-TS, $P2_1$, 0 K	3.615	7.926	20.747	90	94.78	90	0.11
*Acetic Acid Solvate (**S-$C2_{44}$**)*							
SITDIJ, Pc, RT	3.893(2)	8.181(5)	22.563(15)	90	98.43(3)	90	
CryOpt, Pc, 0 K	3.793	8.129	23.109	90	95.28	90	0.23
PBE-TS, Pc, 0 K	3.693	8.191	22.272	90	91.13	90	0.12
*Propionic Acid Solvate (**S-$C3_{44}$**)*							
S-$C4_{44}$, P-1, 173 K	5.218(<1)	6.269(<1)	13.025(<1)	96.04(<1)	100.82(<1)	103.95(<1)	
CryOpt, $P1$ (P-1), 0 K	5.359	6.274	13.376	99.57	101.37	107.70	0.25
PBE-TS, $P1$ (P-1), 0 K	5.207	6.244	12.906	97.19	101.20	106.35	0.13

Table S5 (continued)**.** Quality of the representation of the experimental structures of 2,2'-BIPY and 4,4'-BIPY.

Solid Form	Lattice parameters (cell vectors/Å, angles/°)						*rmsd*$_{15}$ (Å)
	a / Å	*b* / Å	*c* / Å	α / °	β / °	γ / °	
*Butyric Acid Solvate (**S-C4$_{44}$**)*							
S-C3$_{44}$, P-1, 173 K	5.230(3)	6.240(2)	14.119(10)	87.52(4)	83.59(5)	78.10(4)	
CryOpt, *P*1 (*P*-1), 0 K	5.248	6.223	14.656	85.52	81.51	71.70	0.39
PBE-TS, *P*1 (*P*-1), 0 K	5.161	6.198	13.836	89.25	83.17	75.72	0.15
*Valeric Acid Solvate (**S-C5$_{44}$**)*							
S-C5$_{44}$, P-1, 173 K	5.136(1)	6.380(1)	15.658(4)	82.44(2)	89.93(2)	80.60(2)	
CryOpt, *P*1 (*P*-1), 0 K	5.350	5.876	16.554	85.71	90.49	79.42	0.48
PBE-TS, *P*1 (*P*-1), 0 K	5.089	6.354	15.463	82.39	89.68	79.73	0.07
*Caproic Acid Solvate (**S-C6$_{44}$**)*							
S-C6$_{44}$, P-1, 173 K	5.021(<1)	6.654(1)	16.974(2)	100.65(1)	93.11(1)	97.62(1)	
CryOpt, *P*1 (*P*-1), 0 K	5.064	6.856	15.662	102.18	96.57	96.14	0.73
PBE-TS, *P*1 (*P*-1), 0 K	5.237	7.445	14.784	103.94	95.38	95.23	0.45
*Caprylic Acid Solvate (**S-C8$_{44}$**)*							
S-C8$_{44}$, P-1, 173 K	8.911(<1)	9.630(1)	16.471(<1)	99.00(1)	93.96(1)	113.38(1)	
CryOpt, *P*1 (*P*-1), 0 K	8.998	9.828	16.521	100.07	94.32	112.10	0.21
PBE-TS, *P*1 (*P*-1), 0 K	8.924	9.493	16.166	100.38	93.73	114.09	0.11

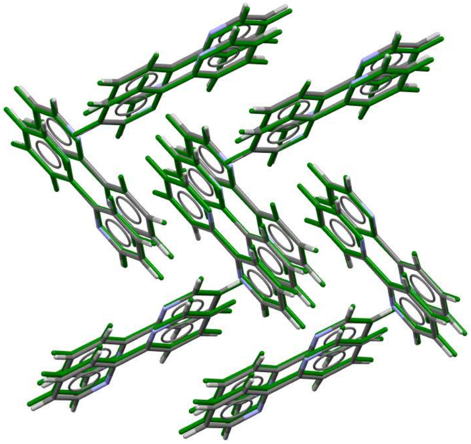

Figure S8. Overlay of the 15-molecule cluster of the observed structure of **AH$_{22}$** (BIPYRL04[3], colored by element) and calculated PBE-TS structure (green), *rmsd*$_{15}$=0.12 Å.

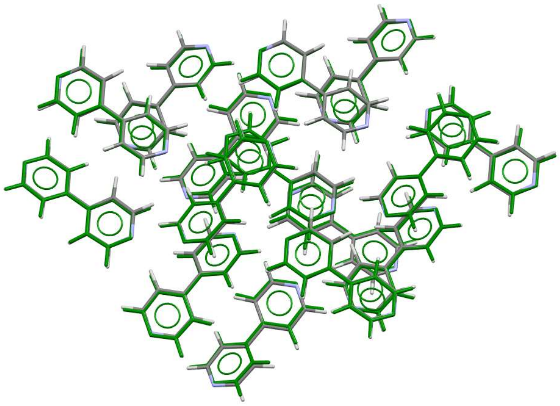

Figure S9. Overlay of the 15-molecule cluster of the observed structure of **AH$_{44}$** (HIQWEJ02[4], colored by element) and calculated PBE-TS structure (green), *rmsd*$_{15}$=0.15 Å.

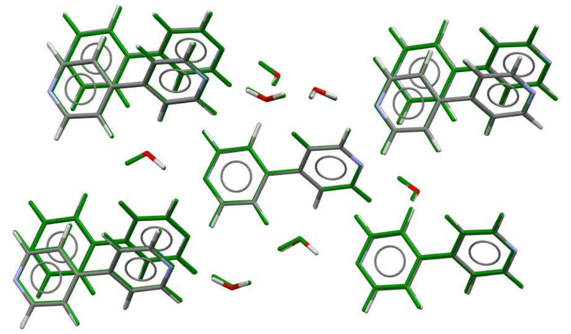

Figure S10. Overlay of the 15-molecule cluster of the observed structure of **Hy2$_{44}$** (WOVYEL02[5], colored by element) and calculated PBE-TS structure (green), *rmsd*$_{15}$=0.06 Å.

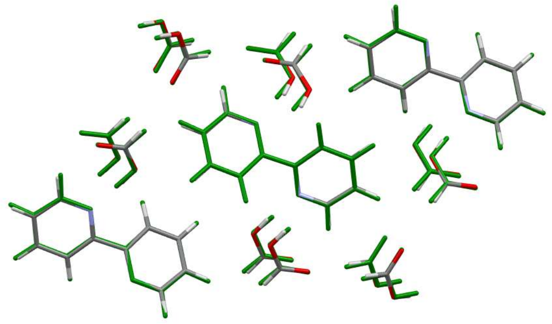

Figure S11. Overlay of the 15-molecule cluster of the observed structure of **S-C1$_{22}$** (colored by element) and calculated PBE-TS structure (green), *rmsd*$_{15}$=0.05 Å.

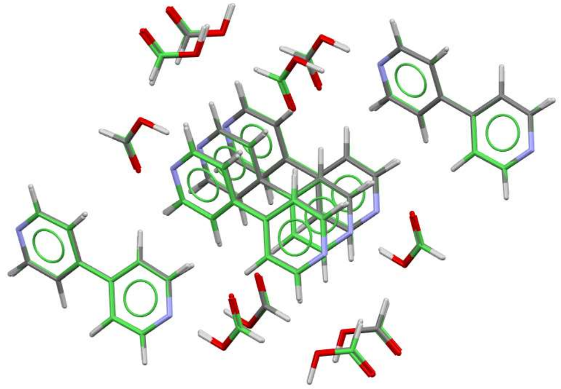

Figure S12. Overlay of the 15-molecule cluster of the observed structure of **S-C1$_{44}$** (GOKCEQ[6], colored by element) and calculated PBE-TS structure (green), *rmsd*$_{15}$=0.11 Å.

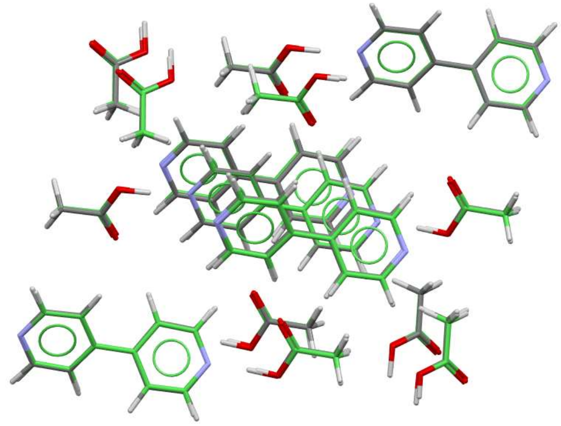

Figure S13. Overlay of the 15-molecule cluster of the observed structure of **S-C2$_{44}$** (SITDIJ[7], colored by element) and calculated PBE-TS structure (green), $rmsd_{15}$=0.12 Å.

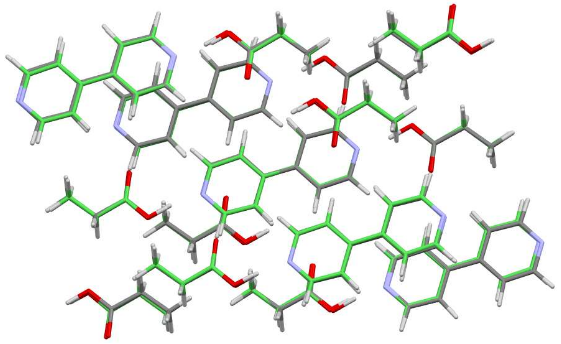

Figure S14. Overlay of the 15-molecule cluster of the observed structure of **S-C3$_{44}$** (colored by element, main disorder component only) and calculated PBE-TS structure (green), $rmsd_{15}$=0.13 Å.

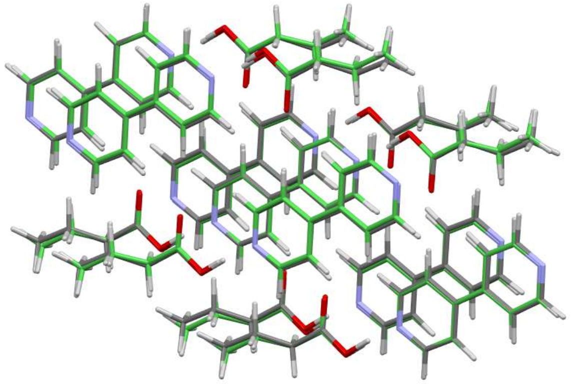

Figure S15. Overlay of the 15-molecule cluster of the observed structure of **S-C4$_{44}$** (colored by element) and calculated PBE-TS structure (green), $rmsd_{15}$=0.15 Å.

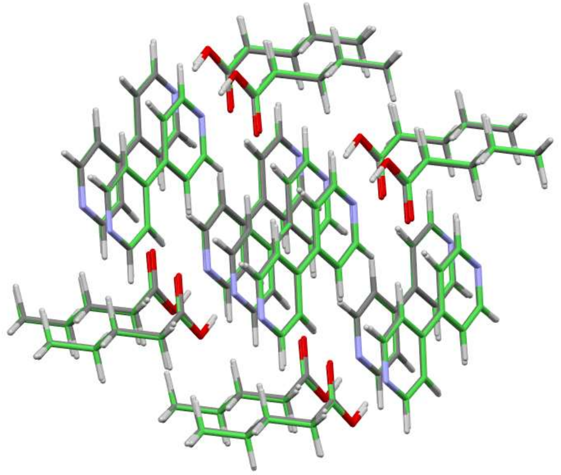

Figure S16. Overlay of the 15-molecule cluster of the observed structure of **S-C5$_{44}$** (colored by element) and calculated PBE-TS structure (green), *rmsd*$_{15}$=0.07 Å.

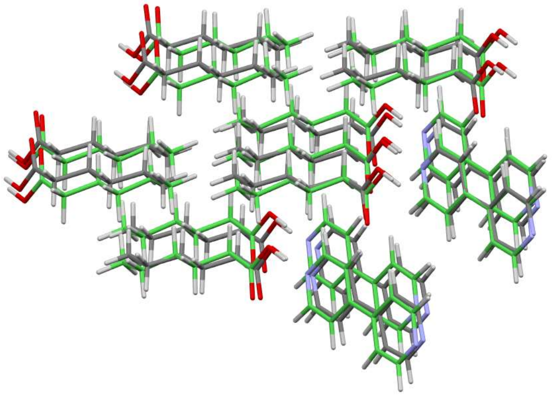

Figure S17. Overlay of the 15-molecule cluster of the observed structure of **S-C6$_{44}$** (one disorder component only, colored by element) and calculated PBE-TS structure (green), *rmsd*$_{15}$=0.45 Å.

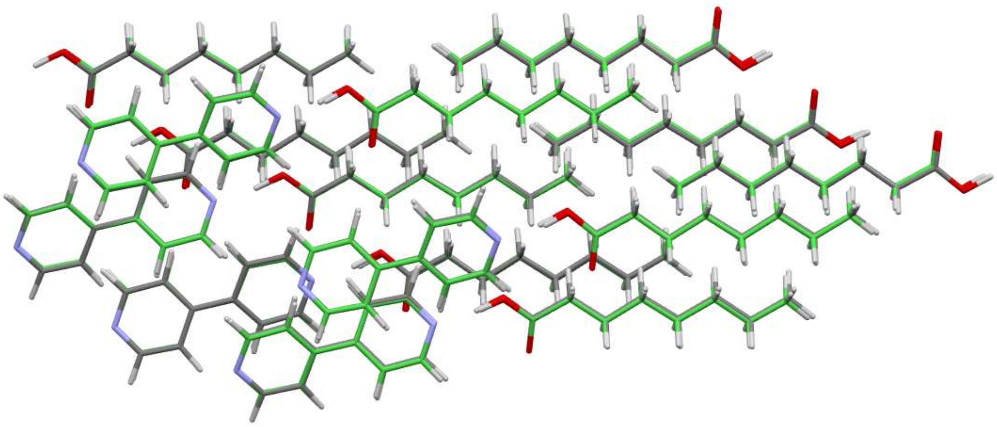

Figure S18. Overlay of the 15-molecule cluster of the observed structure of **S-C8$_{44}$** (colored by element) and calculated PBE-TS structure (green), *rmsd*$_{15}$=0.11 Å.

3.2. Computational generation of low-energy structures

Table S6 provides an overview over the number of structures and energy ranges of the structures selected to be generated/optimized at the different stages of the generation of the lattice energy landscapes.

Table S6. Overview Computational Generation of the Anhydrate Crystal Energy Landscapes.

	**2,2'-Bipyridine (Z'=1)**	**4,4'-Bipyridine (Z'=1)**	**4,4'-Bipyridine (Z'=2)**
CrystalPredictor (rigid body)			
Charges	PBE0/aug-cc-pVTz	PBE0/aug-cc-pVTz	PBE0/aug-cc-pVTz
Number of structures	500000	1000000	1000000
DMACRYS (rigid body)			
Multipoles	PBE0/aug-cc-pVTz	PBE0/aug-cc-pVTz	PBE0/aug-cc-pVTz
Energy range	19 kJ mol^{-1}	19 kJ mol^{-1} (Z'=1)	19 kJ mol^{-1}
Number of structures	15107	12784	10002
CrystalOptimizer (flexible)			
Multipoles	PBE0/aug-cc-pVTz	PBE0/aug-cc-pVTz	PBE0/aug-cc-pVTz
Energy range	7.5 kJ mol^{-1}	7.5 kJ mol^{-1}	7.5 kJ mol^{-1}
Number of structures	197	88	91
CASTEP PBE-TS (cut-off: 780 eV, k-points: 0.07)			
Energy range	5 kJ mol^{-1}	7.5 kJ mol^{-1}	7.5 kJ mol^{-1}
Number of structures	60	40	31
CASTEP PBE-MBD (cut-off: 1100 eV, k-points: 0.07, single point calculations)*			
Energy range	7.5 kJ mol^{-1}	7.5 kJ mol^{-1}	7.5 kJ mol^{-1}
Number of structures	31	25	8

The lowest-energy PBE-TS structures of 2,2′-BIPY and 4,4′-BIPY are given in Tables S6 and S7, respectively.

Table S7. Computationally generated low-energy 2,2′-BIPY PBE-TS structures. The experimental structure is highlighted in grey.

Str.[a]	Space group	Cell parameters						E_{latt}/	PI[b]	$E_{cluster}(15)$[c]/
	(Z′)	*a*/Å	*b*/Å	*c*/Å	*α*/°	*β*/°	*γ*/°	kJ mol⁻¹		kJ mol⁻¹
204	$P2_1/n$ (0.5)	5.514	6.157	11.378	90	96.69	90	−137.04	0.757	−85.4
966	$P2_1/n$ (0.5)	3.713	9.135	11.215	90	95.05	90	−134.77	0.764	−80.2
503	$P2_1/n$ (1)	5.805	11.142	11.916	90	90.35	90	−133.47	0.752	−77.65
240	$P2_1/n$ (1)	6.795	10.977	10.670	90	101.57	90	−132.55	0.744	−84.2
35	$Pna2_1$ (1)	17.992	3.724	11.409	90	90	90	−132.17	0.764	−77.4
118	$P2_1/c$ (0.5)	3.723	9.483	11.008	90	99.32	90	-134.09	0.756	−78
299	$Pbca$ (1)	5.983	12.301	21.143	90	90	90	−133.92	0.747	−80.7
877	$P2_1/n$ (0.5)	5.770	11.786	6.157	90	109.22	90	−133.81	0.735	−82.9
3751	$P2_1/n$ (1)	5.044	13.211	11.754	90	96.79	90	−132.73	0.746	−79.5
244	$C2/c$ (1)	19.643	5.951	13.369	90	94.90	90	−132.54	0.745	−80.05
175	$P2_1/c$ (0.5)	3.734	9.645	10.798	90	94.09	90	−132.40	0.754	−75.3
1050	$P2_1/n$ (1)	5.647	11.954	11.566	90	98.41	90	−132.10	0.751	−77.2
570	$P2_12_12_1$ (1)	3.720	9.048	22.695	90	90	90	−132.10	0.763	−76.6
3011	$P2_12_12_1$ (1)	7.393	9.981	10.675	90	90	90	−131.62	0.743	−82.4
1759	$Pbca$ (1)	11.684	10.657	12.575	90	90	90	−131.61	0.741	−81.15
12	$Pna2_1$ (1)	13.605	4.977	11.669	90	90	90	−131.52	0.737	−78
419	$Pca2_1$ (1)	23.115	3.705	9.092	90	90	90	−131.49	0.750	−76.4
128	$P2_12_12_1$ (1)	5.086	11.739	13.094	90	90	90	−131.43	0.745	−78.6
1132	$P2_1/n$ (1)	5.659	9.271	14.773	90	92.67	90	−131.29	0.750	−77.7
1807	$C2/c$ (1)	10.627	8.572	17.519	90	105.46	90	−131.17	0.755	−78.05
435	$I2/c$ (1)	11.218	11.421	13.212	90	113.51	90	−130.99	0.745	−76.7
65	$Pna2_1$ (1)	18.572	3.742	11.105	90	90	90	−130.87	0.756	−75.3
100	$P2_1/n$ (1)	3.726	9.036	23.024	90	92.75	90	−130.81	0.749	−75.25
654	$P2_1/n$ (1)	5.744	11.130	12.375	90	98.6	90	−130.78	0.744	−77.5
1514	$Pca2_1$ (1)	11.413	9.284	7.415	90	90	90	−130.78	0.742	−82.2
699	$Aba2$ (1)	22.950	6.969	9.863	90	90	90	−130.61	0.737	−80.55
627	$P2_12_12_1$ (1)	5.185	10.519	14.463	90	90	90	−130.57	0.741	−79.7
539	$P2_1/c$ (1)	10.749	6.314	12.177	90	106.65	90	−130.35	0.733	−81.6
253	$P2_1/n$ (1)	7.208	9.501	11.689	90	104.30	90	−130.10	0.746	−77.45
1292	$P2_1/c$ (1)	9.205	11.635	7.339	90	96.81	90	−129.96	0.742	−79.25
1805	$P2_12_12_1$ (1)	6.395	10.951	11.520	90	90	90	−129.79	0.713	−79.7

[a]Structure ID: CrystalPredictor rank; [b]Packing Index calculated using PLATON[8]; [c]Calculated using Crystal Explorer (see manuscript section 2.3).

Table S8. Computationally generated low-energy 4,4′-BIPY PBE-TS structures. The experimental structure is highlighted in grey.

Str.[a]	Space group	Cell parameters						E_{latt}/	PI[b]	$E_{cluster}$(15)[c]/
	(Z′)	*a*/Å	*b*/Å	*c*/Å	*α*/°	*β*/°	*γ*/°	kJ mol^{-1}		kJ mol^{-1}
1333	$P4_22_12$ (0.25)	10.447	10.447	3.633	90	90	90	−147.53	0.726	−83.90
1149	*Pbca* (1)	7.149	11.424	18.420	90	90	90	−147.29	0.773	−96.05
8319	*I*222 (0.25)	3.658	5.783	17.895	90	90	90	−146.13	0.762	−77.26
1874	*C*2/*c* (1)	18.773	3.663	22.765	90	106.14	90	−145.67	0.774	−82.13
5529	*C*2/*c* (0.5)	18.786	3.661	11.210	90	103.90	90	−145.26	0.778	−86.50
7291	*Pbccn* (0.5)	3.659	11.220	18.166	90	90	90	−144.70	0.776	−86.60
862	*Pbca* (2)	18.425	7.115	23.227	90	90.00	90	−144.53	0.765	−93.30
6367	*C*2/*c* (0.5)	6.382	18.476	6.983	90	115.12	90	−144.10	0.768	−96.10
648b	*Pbca* (2)	7.143	11.564	36.738	90	90.00	90	−144.09	0.766	−94.40
7091	*P*2/*c* (2)	14.809	3.631	29.920	90	91.75	90	−143.78	0.715	−82.73
1476	*C*2/*c* (0.5)	11.771	5.883	11.503	90	105.55	90	−143.67	0.755	−91.60
379	*P*21/*c* (0.5)	8.906	5.612	7.480	90	95.00	90	−143.58	0.777	−95.20
2471	*P*-1 (2)	8.557	8.583	10.958	85.18	85.31	80.08	−143.55	0.732	−93.43
67	*C*2/*c* (1)	16.924	5.895	16.674	90	113.2	90	−143.54	0.758	−89.10
9284	*Pbca* (2)	14.648	11.278	18.395	90	90	90	−143.50	0.767	−85.95
125	$P2_1/n$ (0.5)	3.773	5.495	17.987	90	94.84	90	−143.10	0.776	−82.00
763	*I*2/*a* (1)	7.421	5.740	35.475	90	94.58	90	−143.01	0.772	−82.73
84	*Pbcn* (0.5)	5.854	18.532	7.096	90	90	90	−142.80	0.749	−90.60
7646	$P2_1/n$ (1)	12.467	3.718	16.268	90	99.16	90	−142.39	0.776	−89.50
6531	$P2_1/c$ (1)	3.659	5.779	36.016	90	90.75	90	−142.18	0.761	−76.50
692	*Cc* (2)	7.203	5.850	9.039	90	97.65	90	−142.04	0.777	−81.98
771	*I*2/*c* (1)	7.303	5.648	18.490	90	100.49	90	−141.90	0.771	−91.40
1313	*I*2/*a* (1)	19.329	3.650	22.651	90	110.20	90	−141.80	0.769	−87.10
78	$P2_1/c$ (1)	9.883	7.055	11.732	90	110.78	90	−141.60	0.759	−91.95
312	$P2_1/n$ (1)	8.037	5.717	16.403	90	94.09	90	−141.41	0.771	−95.50
179	$P2_1/c$ (1)	9.664	10.765	7.700	90	100.60	90	−141.30	0.736	−91.65
8367	*Pbca* (1)	5.876	7.109	36.550	90	90	90	−141.29	0.761	−86.60
1631	$P2_1/n$ (2)	9.712	7.021	22.546	90	95.75	90	−141.24	0.759	−91.60
1383	*C*2/*c* (1)	18.437	5.756	14.572	90	101.19	90	−141.21	0.764	−88.85
2249	*Pcab* (2)	18.446	6.993	23.737	90	90	90	−140.97	0.757	−91.68
1532	$C222_1$ (0.5)	5.634	18.589	7.188	90	90	90	−140.74	0.766	−90.20
268	$P2_1/c$ (1)	10.048	6.170	13.016	90	109.67	90	−140.45	0.766	−90.35
8415	*Pbcn* (0.5)	5.813	7.175	18.235	90	90	90	−140.21	0.744	−86.90

[a]Structure ID: CrystalPredictor rank; [b]Packing Index calculated using PLATON[8]; [c]Calculated using Crystal Explorer (see manuscript section 2.3).

3.3. Structure family similarity trees and Packing comparisons

The structure family similarity trees were calculated using the CCDC API packing similarity dendrogram script with clustering type settings “complete”.

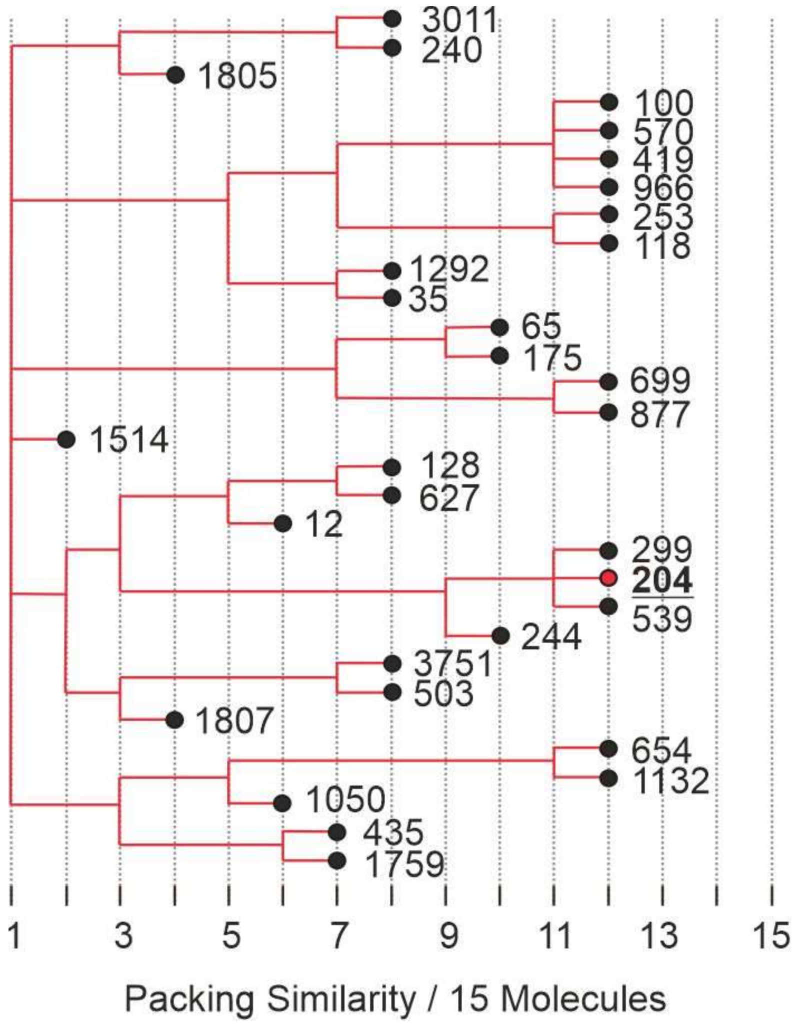

Figure S19. Structure family similarity tree, showing the crystal packing similarity between the lowest-energy crystal structures from the 2,2’-BIPY CSP search. Experimental structure: 204.

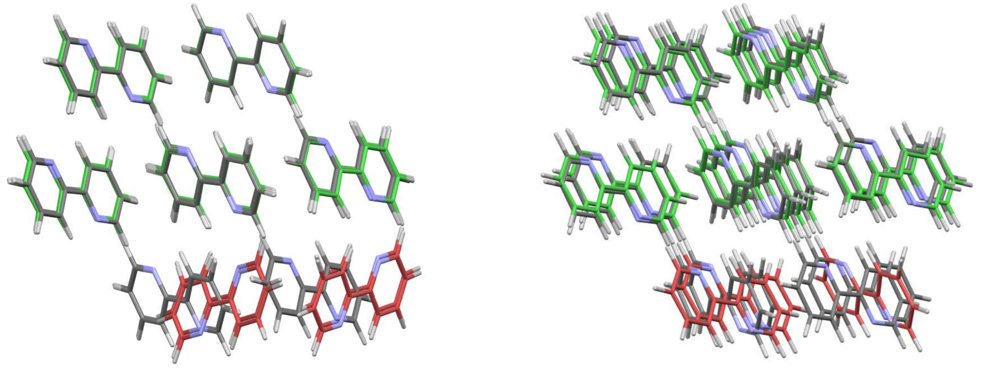

(a) 204 (**AH₂₂**) and 299 ($rmsd_{11}$ = 0.15 Å)   (b) 204 (**AH₂₂**) and 539 ($rmsd_{11}$ = 0.26 Å)

Figure S20. Packing comparisons of selected 2,2'-BIPY structures. Missed matches are shown in red.

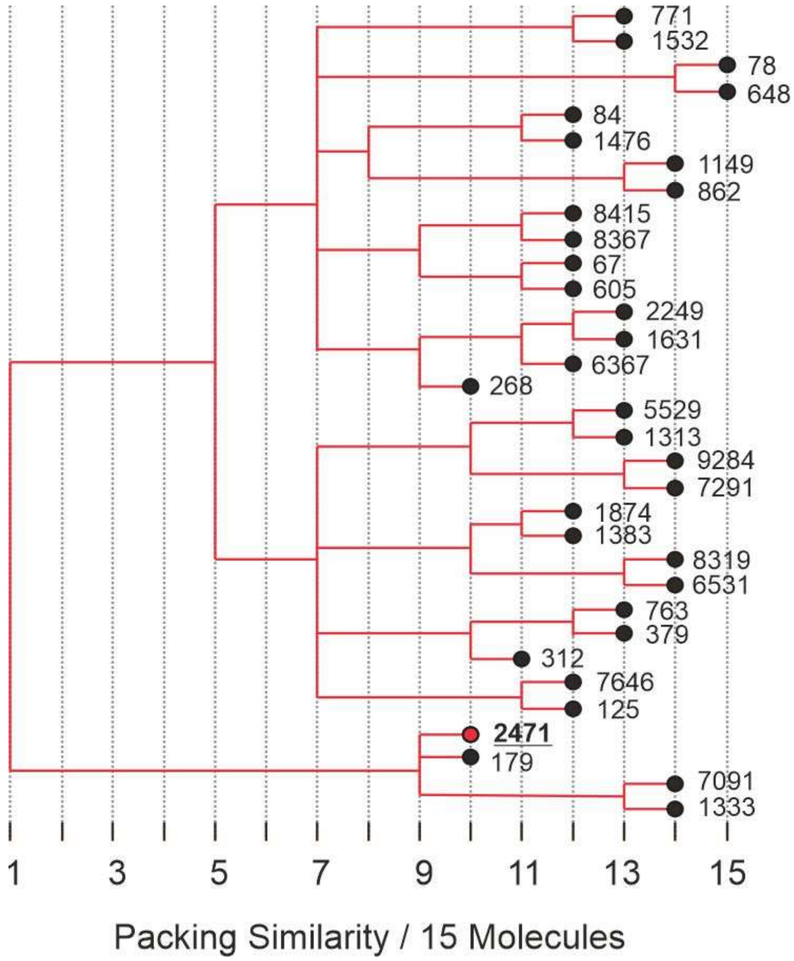

Figure S21. Structure family similarity tree, showing the crystal packing similarity between the lowest-energy crystal structures from the 4,4'-BIPY CSP searches. Experimental structure: 2471.

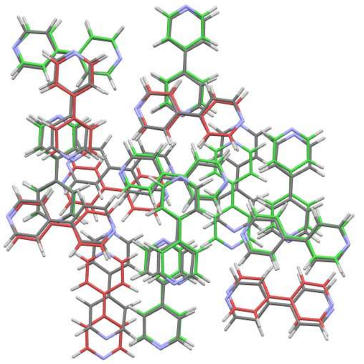

(a) 2471 ($\mathbf{AH}_{\mathbf{44}}$) and 179 ($rmsd_9$ = 0.55Å)

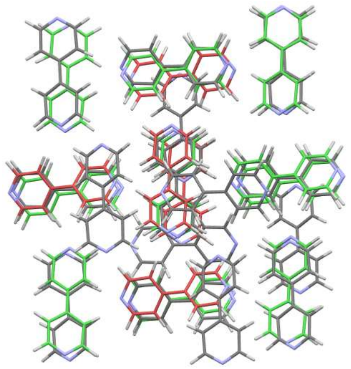

(b) 2471 ($\mathbf{AH}_{\mathbf{44}}$) and 7091 ($rmsd_9$ = 1.02 Å)

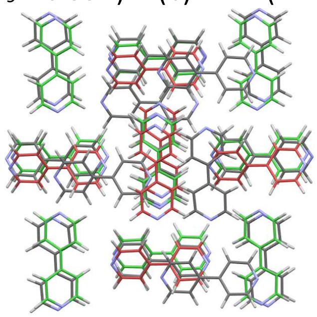

(c) 2471 ($\mathbf{AH}_{\mathbf{44}}$) and 1333 ($rmsd_9$ = 0.92 Å)

Figure S22. Packing comparisons of selected 4,4'-BIPY structures. Missed matches are shown in red. Note that one layer is identical for all structures.

4. Substitution calculations

Substitution calculations were performed to investigate the potential of isostructural solvates.

The acetic acid molecule was pasted into the **S-C1$_{44}$** structure ($P2_1$) and optimized with CASTEP as described in the manuscript in section 2.2. The obtained structure was calculated to be 0.71 kJ mol^{-1} less stable in lattice energy. An overlay of **S-C1$_{44}$** and the hypothetical $P2_1$ structure is given in Figure S23.

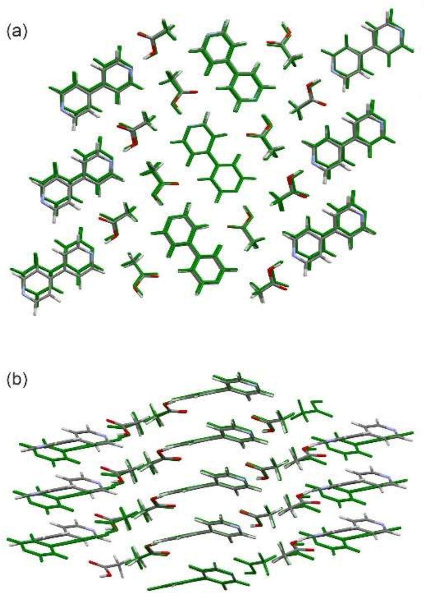

Figure S23. Overlay of **S-C2$_{44}$** (colored by element) and a hypothetical $P2_1$ acetic acid solvate structure (green, isostructural with **S-C1$_{44}$**), viewed along the (a) *a* and (b) *b* crystallographic axes.

Similarly, the acetic acid molecule was pasted into the triclinic 4,4'-BIPY solvate structures (**S-C3$_{44}$**, **S-C4$_{44}$**, **S-C5$_{44}$**, **S-C6$_{44}$** and **S-C8$_{44}$**). The isomorphous acetic acid solvates were calculate to be higher in lattice energy: in **S-C3$_{44}$** +14.88 kJ mol^{-1}, in **S-C4$_{44}$** +30.39 kJ mol^{-1} and other solvate structures even less stable.

The .res file for the $P2_1$ acetic acid solvate structure is given below:

```
TITL S-C2-44 in S-C1-44
CELL 0.71073 3.7256 8.0602 22.4415 90 90.775 90
ZERR 2 0.0000 0.0000 0.0000 0 0.000 0
LATT -1
SYMM -x,1/2+y,-z
SFAC C H N O
UNIT 28 32 4 8
FVAR 1.00
C1      1  0.382840  0.560000  0.195490  1.000000 0.050000
C2      1  0.600760  0.292900  0.227810  1.000000 0.050000
C3      1  0.458390  0.449310  0.241410  1.000000 0.050000
C4      1  0.655770  0.254720  0.167680  1.000000 0.050000
C5      1  0.574220  0.373780  0.124810  1.000000 0.050000
C6      1  0.789570 -0.104350  0.311800  1.000000 0.050000
C7      1  0.685100  0.001900  0.265950  1.000000 0.050000
C8      1  0.697400  0.173820  0.275420  1.000000 0.050000
C9      1  0.813830  0.230160  0.331560  1.000000 0.050000
C10     1  0.914210  0.115100  0.374710  1.000000 0.050000
C11     1  0.185780  0.891920  0.080940  1.000000 0.050000
C12     1  0.097620  1.019020  0.033730  1.000000 0.050000
C13     1  0.255510  0.607870  0.424670  1.000000 0.050000
C14     1  0.409500  0.492300  0.470980  1.000000 0.050000
H1      2  0.269660  0.682770  0.203680  1.000000 0.060000
H2      2  0.402370  0.484660  0.287210  1.000000 0.060000
H3      2  0.772200  0.136120  0.154690  1.000000 0.060000
H4      2  0.617460  0.349120  0.077630  1.000000 0.060000
H5      2  0.787660 -0.238850  0.306520  1.000000 0.060000
H6      2  0.594350 -0.048610  0.223380  1.000000 0.060000
H7      2  0.837050  0.361900  0.341380  1.000000 0.060000
H8      2  1.010670  0.155280  0.418510  1.000000 0.060000
H9      2  0.386170  0.667390  0.092220  1.000000 0.075000
H10     2  0.342030  1.069650  0.014040  1.000000 0.075000
H11     2 -0.057630  0.960990 -0.002470  1.000000 0.075000
H12     2 -0.056650  1.121610  0.052130  1.000000 0.075000
H13     2  0.031870  0.827100  0.411950  1.000000 0.075000
H14     2  0.193050  0.421170  0.491670  1.000000 0.075000
H15     2  0.548490  0.560860  0.506420  1.000000 0.075000
H15     2  0.592020  0.403470  0.450610  1.000000 0.075000
N1      3  0.442600  0.524190  0.138040  1.000000 0.050000
N2      3  0.902520 -0.049120  0.365260  1.000000 0.050000
O1      4  0.348740  0.759200  0.058980  1.000000 0.050000
O2      4  0.113160  0.909430  0.133890  1.000000 0.050000
O3      4  0.129340  0.748040  0.446970  1.000000 0.050000
O4      4  0.240480  0.573690  0.371170  1.000000 0.050000
END
```

5. Morphology of the solvates

The morphologies of the solvates were recoded using an Olympus SZX12 stereo-microscope equipped with an Olympus DP71 digital camera (Olympus, A).

5.1. 2,2'-Bipyridine formic acid solvate

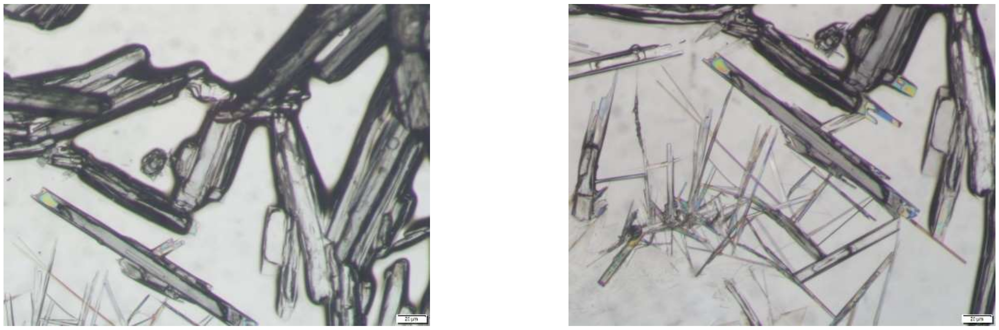

Figure S24. Photographs of **S-C1**$_{22}$. The thin crystals crystallized during taking the photographs (solvent evaporation).

5.2. 4,4'-Bipyridine formic acid solvate

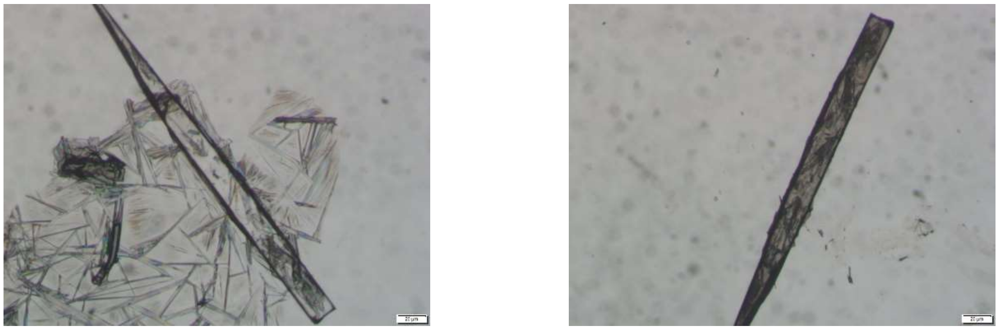

Figure S25. Photographs of **S-C1**$_{44}$. Crystal on the right already started to desolvate.

5.3. 4,4'-Bipyridine acetic acid solvate

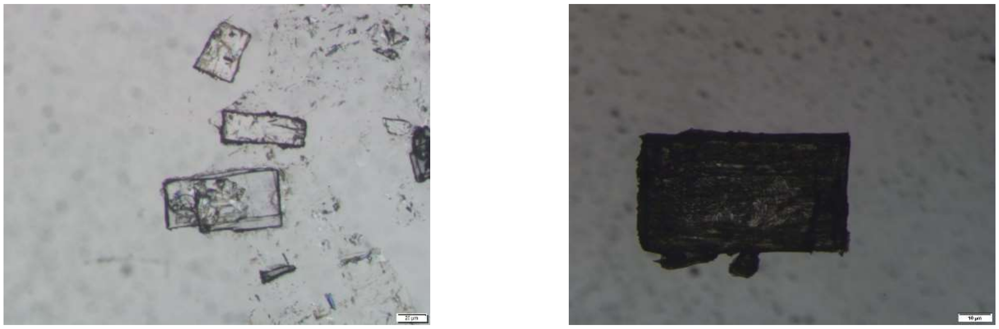

Figure S26. Photographs of **S-C2**$_{44}$. Crystal on the right shows pseudomorphosis (desolvated to the anhydrate).

5.4. 4,4’-Bipyridine propionic acid solvate

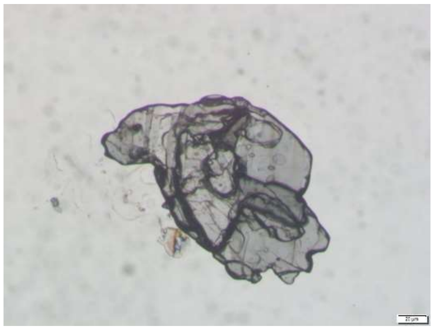

Figure S27. Photograph of **S-C3$_{44}$**.

5.5. 4,4’-Bipyridine butyric acid solvate

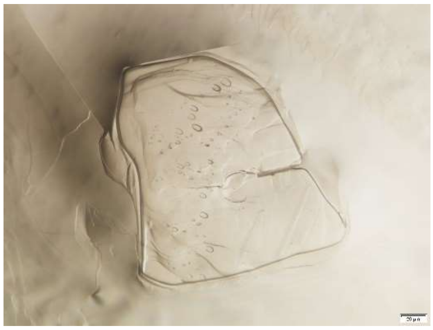

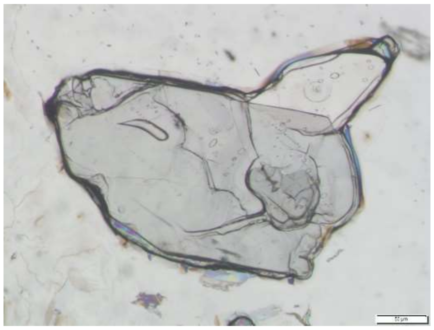

Figure S28. Photographs of **S-C4$_{44}$**.

5.6. 4,4’-Bipyridine valeric acid solvate

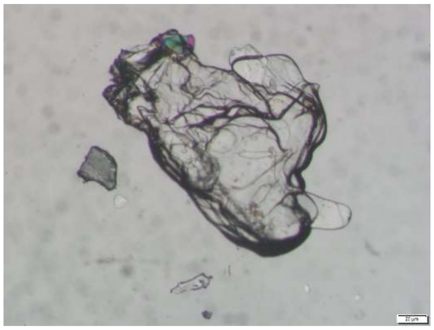

Figure S29. Photograph of **S-C5$_{44}$**.

5.7. 4,4'-Bipyridine caproic acid solvate

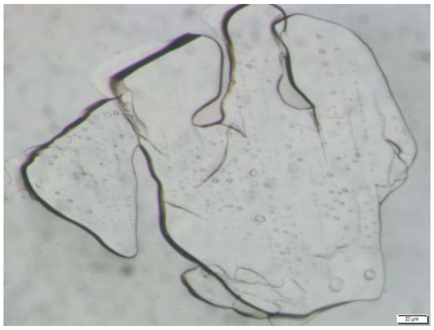

Figure S30. Photograph of **S-C6₄₄**.

5.8. 4,4'-Bipyridine caprylic acid solvate

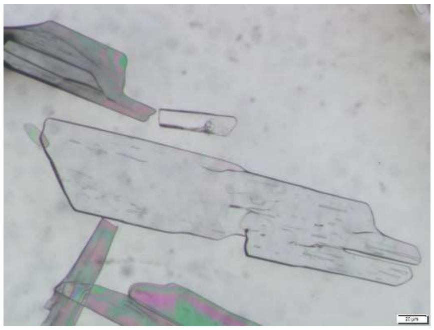

Figure S31. Photograph of **S-C8₄₄**.

6. Crystallographic information

6.1. 2,2'-BIPY formic acid solvate (S-C1$_{22}$)

Table S9. Crystallographic data for **S-C1$_{22}$**.

Crystal data	
Chemical formula	$C_{10}H_8N_2·2(CH_2O_2)$
M_r	248.24
Crystal system, space group	Orthorhombic, *Pbc*2$_1$
Temperature (K)	193
a, *b*, *c* (Å)	3.7087 (6), 15.9130 (2), 19.8320 (3)
V (Å^3)	1170.42 (19)
Z	4
Radiation type	Mo *K*a
m (mm^{-1})	0.11
Crystal size (mm)	0.59 × 0.22 × 0.16
Data collection	
Diffractometer	Xcalibur, Ruby, Gemini ultra
Absorption correction	*CrysAlis PRO* 1.171.40.84a (Rigaku Oxford Diffraction, 2020) Empirical absorption correction using spherical harmonics, implemented in SCALE3 ABSPACK scaling algorithm.
T_{min}, T_{max}	0.959, 0.987
No. of measured, independent and observed [*I* > 2s(*I*)] reflections	5849, 2462, 1613
R_{int}	0.066
$(\sin \theta/l)_{max}$ (Å^{-1})	0.670
Refinement	
$R[F^2 > 2s(F^2)]$, $wR(F^2)$, *S*	0.08, 0.226, 1.06
No. of reflections	2462
No. of parameters	177
No. of restraints	7
H-atom treatment	H atoms treated by a mixture of independent and constrained refinement
Largest diff. peak and hole (e Å^{-3})	0.53, -0.42

6.2. 4,4'-BIPY propionic acid solvate (S-C3$_{44}$)

Table S10. Crystallographic data for **S-C3$_{44}$**.

Crystal data	
Chemical formula	$C_3H_6O_2 \cdot 0.5(C_{10}H_8N_2)$
M_r	152.17
Crystal system, space group	Triclinic, $P\bar{}1$
Temperature (K)	193
a, *b*, *c* (Å)	5.2183 (6), 6.2686 (7), 13.0250 (2)
a, b, g (°)	96.043 (1), 100.824 (1), 103.952 (1)
V (Å^3)	401.04 (6)
Z	2
Radiation type	Mo *K*a
m (mm^{-1})	0.09
Crystal size (mm)	0.97 × 0.61 × 0.15
Data collection	
Diffractometer	Xcalibur, Ruby, Gemini ultra
Absorption correction	*CrysAlis PRO* 1.171.40.84a (Rigaku Oxford Diffraction, 2020) Empirical absorption correction using spherical harmonics, implemented in SCALE3 ABSPACK scaling algorithm.
T_{min}, T_{max}	0.651, 1.000
No. of measured, independent and observed [*I* > 2s(*I*)] reflections	2822, 1846, 1282
R_{int}	0.020
(sin θ/l)$_{max}$ (Å^{-1})	0.701
Refinement	
$R[F^2 > 2s(F^2)]$, $wR(F^2)$, S	0.051, 0.147, 1.03
No. of reflections	1846
No. of parameters	126
No. of restraints	37
H-atom treatment	H atoms treated by a mixture of independent and constrained refinement
Largest diff. peak and hole (e Å^{-3})	0.16, -0.21

6.3. 4,4'-BIPY butyric acid solvate (S-C4$_{44}$)

Table S11. Crystallographic data for **S-C4$_{44}$**.

Crystal data	
Chemical formula	$C_4H_8O_2 \cdot 0.5(C_{10}H_8N_2)$
M_r	166.2
Crystal system, space group	Triclinic, $P\bar{1}$
Temperature (K)	193
a, *b*, *c* (Å)	5.230 (3), 6.240 (2), 14.119 (10)
a, b, g (°)	87.52 (4), 83.59 (5), 78.10 (4)
V (Å^3)	448.0 (4)
Z	2
Radiation type	Mo *K*a
m (mm^{-1})	0.09
Crystal size (mm)	0.76 × 0.28 × 0.11
Data collection	
Diffractometer	Xcalibur, Ruby, Gemini ultra
Absorption correction	*CrysAlis PRO* 1.171.40.84a (Rigaku Oxford Diffraction, 2020) Empirical absorption correction using spherical harmonics, implemented in SCALE3 ABSPACK scaling algorithm.
T_{min}, T_{max}	0.306, 1.000
No. of measured, independent and observed [I > 2s(I)] reflections	2825, 1838, 586
R_{int}	0.053
(sin θ/l)$_{max}$ (Å^{-1})	0.676
Refinement	
$R[F^2 > 2s(F^2)]$, $wR(F^2)$, S	0.119, 0.397, 0.98
No. of reflections	1838
No. of parameters	112
H-atom treatment	H-atom parameters constrained
Largest diff. peak and hole (e Å^{-3})	0.34, -0.30

6.4. 4,4'-BIPY valeric acid solvate (S-C5$_{44}$)

Table S12. Crystallographic data for **S-C5$_{44}$**.

Crystal data	
Chemical formula	$0.5(C_{10}H_8N_2)\cdot C_5H_{10}O_2$
M_r	180.22
Crystal system, space group	Triclinic, *P*¯1
Temperature (K)	193
a, *b*, *c* (Å)	5.1355 (11), 6.3797 (12), 15.658 (4)
a, b, g (°)	82.443 (19), 89.93 (2), 80.595 (17)
V (Å^3)	501.60 (19)
Z	2
Radiation type	Mo *K*a
m (mm^{-1})	0.08
Crystal size (mm)	0.54 × 0.31 × 0.14
Data collection	
Diffractometer	Xcalibur, Ruby, Gemini ultra
Absorption correction	*CrysAlis PRO* 1.171.40.84a (Rigaku Oxford Diffraction, 2020) Empirical absorption correction using spherical harmonics, implemented in SCALE3 ABSPACK scaling algorithm.
T_{min}, T_{max}	0.637, 1.000
No. of measured, independent and observed [$I > 2s(I)$] reflections	3024, 2036, 1032
R_{int}	0.040
$(\sin \theta/l)_{max}$ (Å^{-1})	0.683
Refinement	
$R[F^2 > 2s(F^2)]$, $wR(F^2)$, S	0.064, 0.168, 1.00
No. of reflections	2036
No. of parameters	124
No. of restraints	1
H-atom treatment	H atoms treated by a mixture of independent and constrained refinement
Largest diff. peak and hole (e Å^{-3})	0.20, -0.24

6.5. 4,4'-BIPY caproic acid solvate (S-C6$_{44}$)

Table S13. Crystallographic data for **S-C6$_{44}$**.

Crystal data	
Chemical formula	$C_6H_{12}O_2 \cdot 0.5(C_{10}H_8N_2)$
M_r	194.25
Crystal system, space group	Triclinic, $P\bar{1}$
Temperature (K)	193
a, *b*, *c* (Å)	5.0207 (7), 6.654 (1), 16.974 (2)
a, b, g (°)	100.654 (12), 93.111 (12), 97.602 (12)
V (Å^3)	550.58 (13)
Z	2
Radiation type	Mo *K*a
m (mm^{-1})	0.08
Crystal size (mm)	0.54 × 0.31 × 0.14
Data collection	
Diffractometer	Xcalibur, Ruby, Gemini ultra
Absorption correction	*CrysAlis PRO* 1.171.40.84a (Rigaku Oxford Diffraction, 2020) Empirical absorption correction using spherical harmonics, implemented in SCALE3 ABSPACK scaling algorithm.
T_{min}, T_{max}	0.619, 1.000
No. of measured, independent and observed [I > 2s(I)] reflections	3567, 2310, 1309
R_{int}	0.027
(sin θ/l)$_{max}$ (Å^{-1})	0.687
Refinement	
$R[F^2 > 2s(F^2)]$, $wR(F^2)$, S	0.056, 0.155, 0.98
No. of reflections	2310
No. of parameters	180
No. of restraints	127
H-atom treatment	H atoms treated by a mixture of independent and constrained refinement
Largest diff. peak and hole (e Å^{-3})	0.16, -0.15

6.6. 4,4’-BIPY caprylic acid solvate (S-C8$_{44}$)

Table S14. Crystallographic data for **S-C8$_{44}$**.

Crystal data	
Chemical formula	$C_{10}H_8N_2 \cdot 2(C_8H_{16}O_2)$
M_r	444.6
Crystal system, space group	Triclinic, $P\bar{1}$
Temperature (K)	193
a, *b*, *c* (Å)	8.9108 (9), 9.6304 (12), 16.4710 (2)
a, b, g (°)	98.997 (11), 93.962 (10), 113.384 (11)
V (Å^3)	1267.9 (2)
Z	2
Radiation type	Mo *K*a
m (mm^{-1})	0.08
Crystal size (mm)	0.98 × 0.23 × 0.11
Data collection	
Diffractometer	Xcalibur, Ruby, Gemini ultra
Absorption correction	*CrysAlis PRO* 1.171.40.84a (Rigaku Oxford Diffraction, 2020) Empirical absorption correction using spherical harmonics, implemented in SCALE3 ABSPACK scaling algorithm.
T_{min}, T_{max}	0.955, 1.000
No. of measured, independent and observed [I > 2s(I)] reflections	9155, 5833, 3635
R_{int}	0.050
(sin θ/l)$_{max}$ (Å^{-1})	0.705
Refinement	
$R[F^2 > 2s(F^2)]$, $wR(F^2)$, S	0.054, 0.145, 1.02
No. of reflections	5833
No. of parameters	300
No. of restraints	2
H-atom treatment	H atoms treated by a mixture of independent and constrained refinement
Largest diff. peak and hole (e Å^{-3})	0.27, -0.21

6.7. 4,4'-BIPY butyric acid solvate (S-$C4_{44}$) – res file

```
TITL S-C444
CELL 0.71073 5.23 6.24 14.119 87.52 83.59 78.10
ZERR 2 0.003 0.002 0.010 0.04 0.05 0.04
LATT 1
SFAC C H N O
UNIT 18 24 2 4
FVAR 1.00
C1    1  0.396900  0.966400  0.534800  1.000000 0.036000 0.040000 =
       0.070000 -0.007000 -0.008000 -0.013000
N1    3  0.013200  0.840200  0.665800  1.000000 0.060000 0.054000 =
       0.086000 -0.005000 -0.001000 -0.020000
C2    1  0.286200  0.788300  0.519300  1.000000 0.061000 0.033000 =
       0.083000 -0.007000 -0.009000 -0.011000
H2    2  0.340542  0.705670  0.462999  1.000000 0.070000
C3    1  0.311400  1.075800  0.617300  1.000000 0.061000 0.054000 =
       0.079000 -0.009000 0.007000 -0.031000
H3    2  0.384624  1.195955  0.632031  1.000000 0.075000
C4    1  0.097700  0.732800  0.586100  1.000000 0.062000 0.049000 =
       0.096000 0.004000 -0.003000 -0.023000
H4    2  0.024190  0.610601  0.574277  1.000000 0.081000
C5    1  0.116000  1.010300  0.679700  1.000000 0.062000 0.059000 =
       0.086000 -0.021000 0.001000 -0.022000
H5    2  0.053166  1.092755  0.735606  1.000000 0.081000
O1    4  0.650800  0.461100  0.699600  1.000000 0.103000 0.063000 =
       0.115000 -0.020000 0.026000 -0.043000
O2    4  0.662300  0.724800  0.796300  1.000000 0.091000 0.066000 =
       0.091000 -0.010000 0.021000 -0.035000
H1    2  0.769444  0.757248  0.751913  1.000000 0.123000
C6    1  0.574900  0.554500  0.772700  1.000000 0.076000 0.046000 =
       0.099000 0.005000 0.015000 -0.015000
C7    1  0.375000  0.491300  0.846300  1.000000 0.094000 0.058000 =
       0.148000 0.009000 0.050000 -0.020000
H7'1  2  0.366349  0.579149  0.903746  1.000000 0.125000
H7'2  2  0.200904  0.529478  0.821708  1.000000 0.125000
C8    1  0.425300  0.259800  0.874200  1.000000 0.094000 0.069000 =
       0.125000 0.005000 0.005000 -0.037000
H8'1  2  0.594937  0.222887  0.901995  1.000000 0.114000
H8'2  2  0.442059  0.171228  0.816490  1.000000 0.114000
C9    1  0.212000  0.197500  0.945900  1.000000 0.085000 0.096000 =
       0.113000 0.016000 0.008000 -0.047000
H9'1  2  0.235850  0.240349  1.009748  1.000000 0.144000
H9'2  2  0.223543  0.038809  0.945232  1.000000 0.144000
H9'3  2  0.039266  0.273190  0.928784  1.000000 0.144000
END
```

7. 2,2'-BIPY and 4,4'-BIPY Anhydrates and Hydrate

7.1. Powder X-ray diffractometry

The experimentally measured PXRD patterns are contrasted to the from the single crystal structure simulated PXRD patterns (Figure S32).

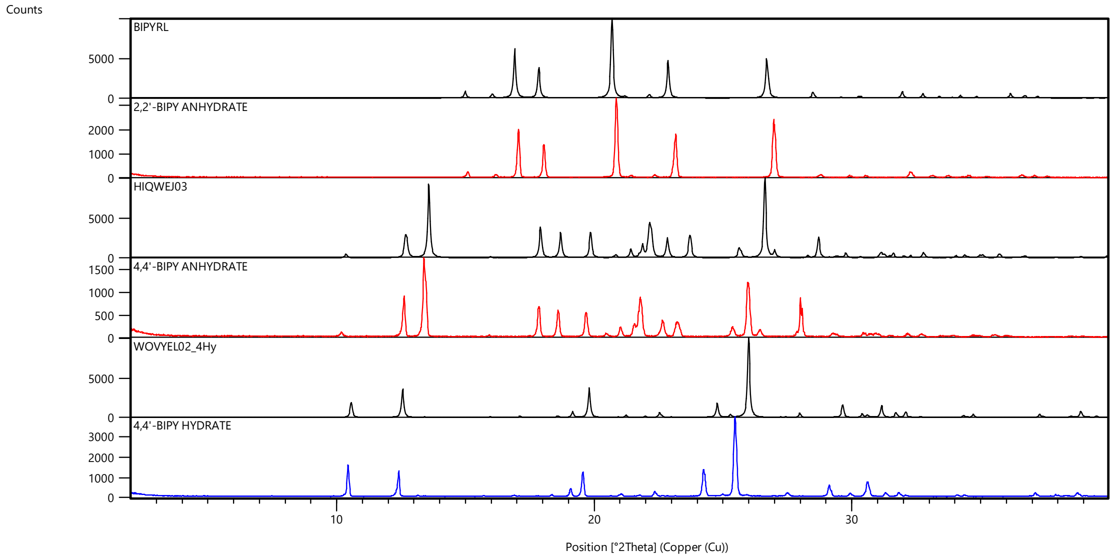

Figure S32. Comparison of experimental 2,2'- and 4,4'-BIPY powder patterns (red – anhydrates, blue – hydrate) to the from the single crystal structures simulated PXRD patterns (black).

7.2. Infrared spectroscopy

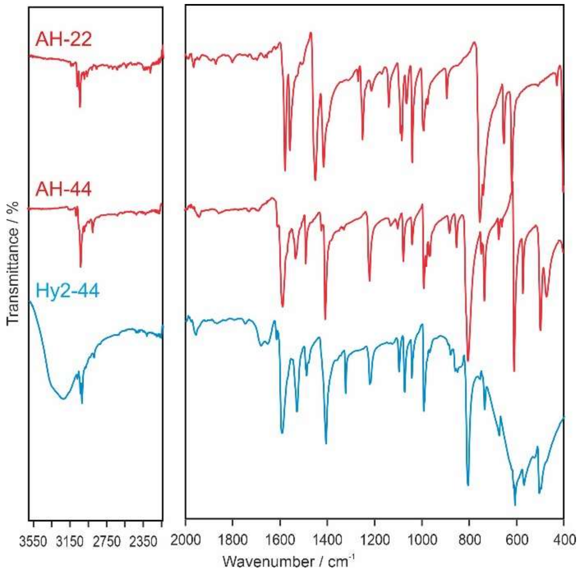

Figure S33. Comparison of 2,2'- and 4,4'-BIPY IR spectra (red – anhydrates, blue – hydrate).

7.3. Gravimetric moisture (de)sorption

The time vs. mass curve (water content) of 2,2'-BIPY shows a continuous mass loss, nearly independent of the relative humidity (Figure S34). The mass loss can be related to sublimation of the compound.

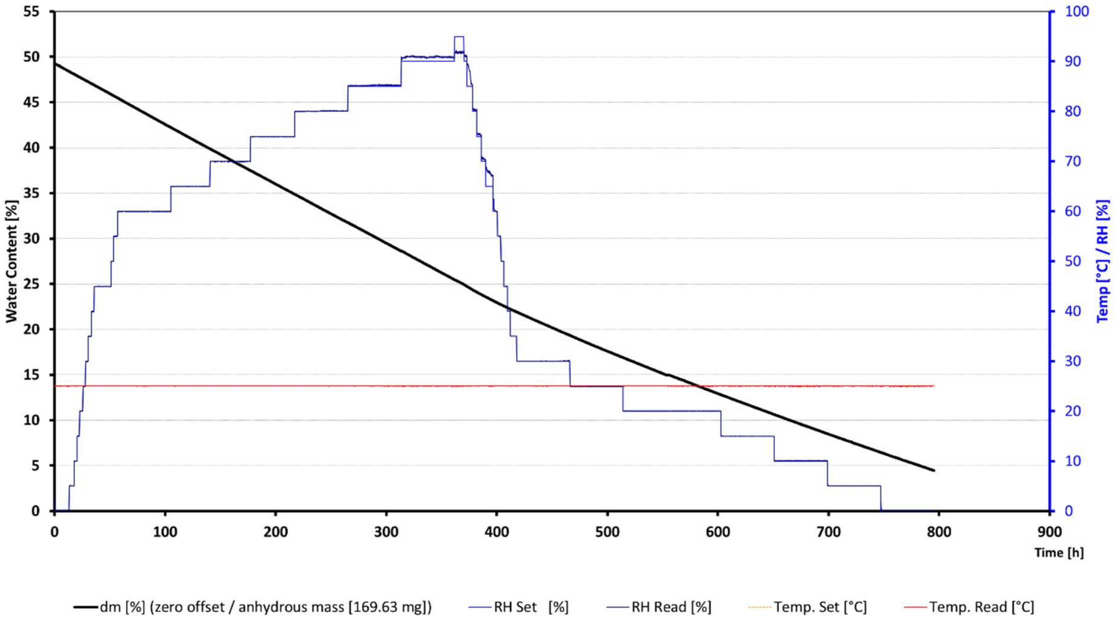

Figure S34. Time vs. mass (expressed as water content) curve of the 2,2'-BIPY anhydrate moisture sorption/desorption experiment recorded at 25 °C.

8. Solvates of 2,2'-BIPY and 4,4'-BIPY

8.1. Powder X-ray diffractometry

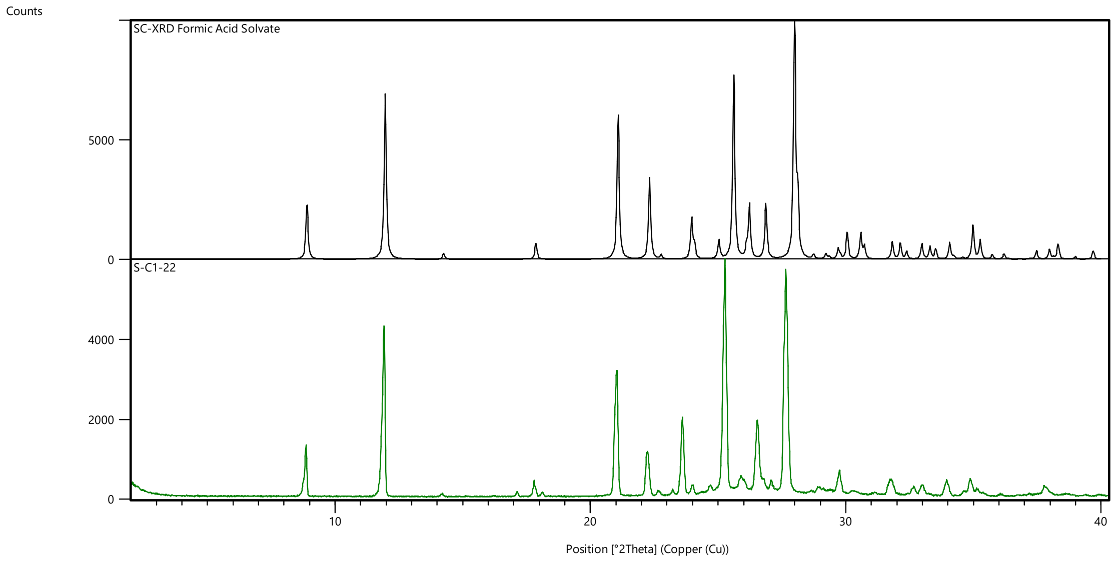

Figure S35. Comparison of the PXRD pattern of **S-C1$_{22}$** (green) to the from the single crystal structure simulated PXRD pattern (black).

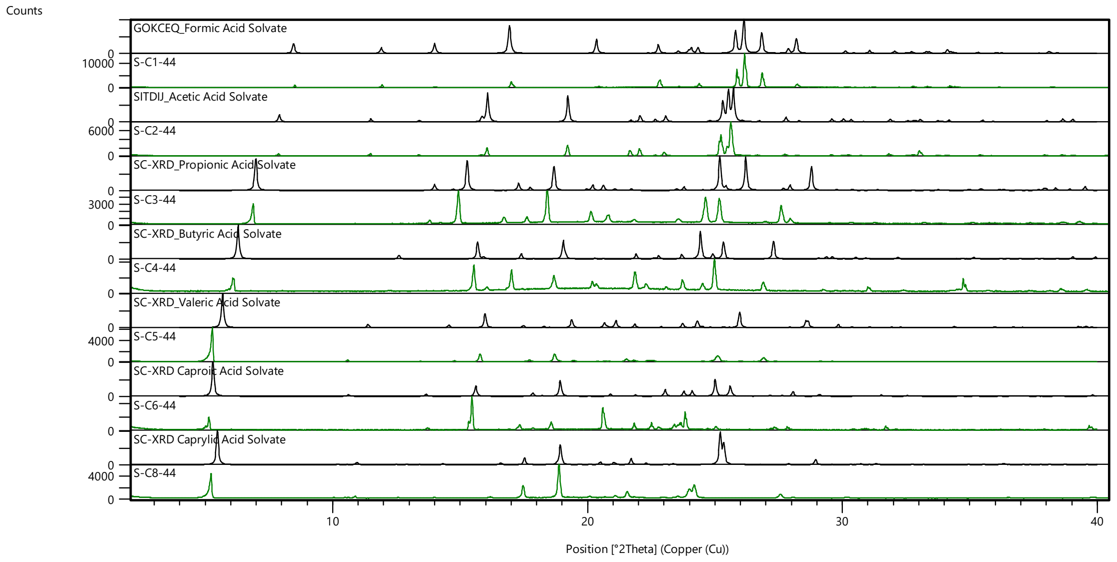

Figure S36. Comparison of the PXRD patterns of 4,4'-BIPY solvates (green) to the from the single crystal structures simulated PXRD patterns (black).

8.2. Infrared spectroscopy

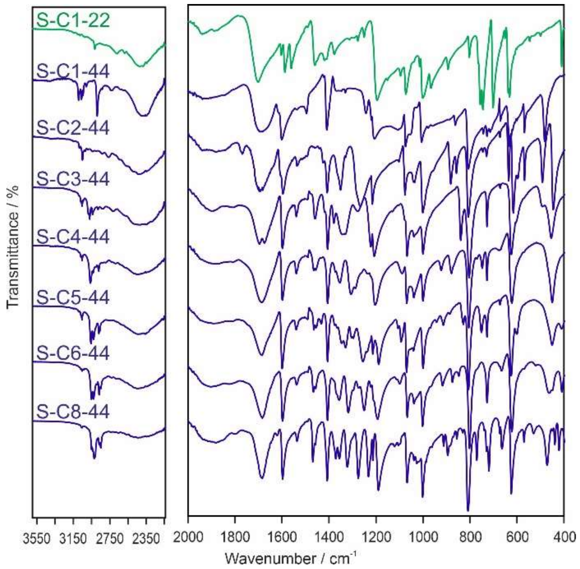

Figure S37. Comparison of the IR spectra of 2,2'-BIPY (green) and 4,4'-BIPY solvates (blue).

8.3. Hot-stage microscopy

Figure S38 shows exemplarily a contact preparation (melt film) of **S-C2$_{44}$** and **AH$_{44}$**. The "black line" at the contact zone of the two compounds relates to the eutectic temperature and forms at approx. 70 °C.

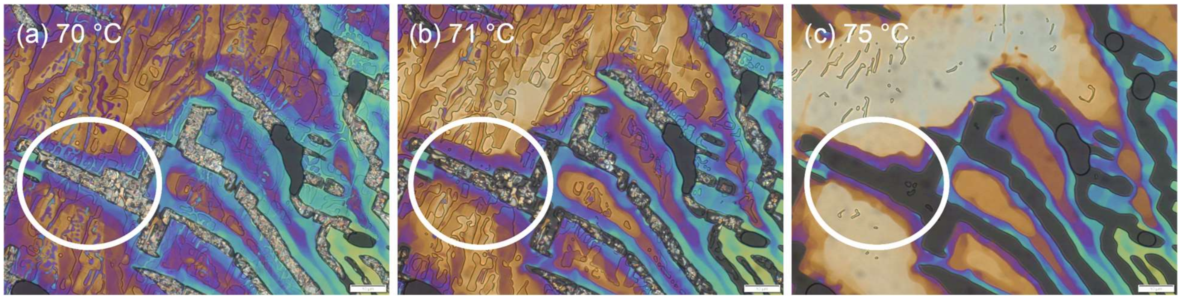

Figure S38. Contact preparation of **S-C2$_{44}$** and **AH$_{44}$** showing the eutectic temperature at 70 °C (encircled).

9. Stability at ambient conditions

All solvates were subjected to storage stability experiments at ambient conditions (RT, 30 – 40% RH), and transformations monitored with PXRD. The diffractograms were recorded at first hourly and then daily. Only a selection of the recorded diffractograms is given.

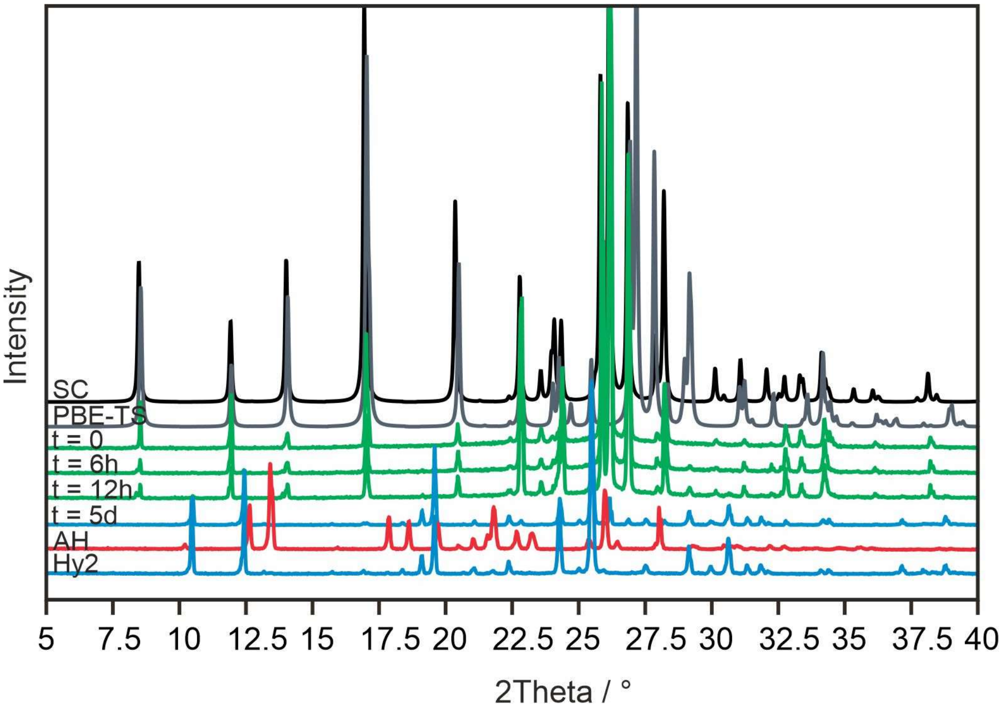

Figure S39. PXRD measurements monitoring the desolvation process of **S-C1$_{44}$** at ambient conditions (t = x hours). Reference patterns are provided for the anhydrate (AH), hydrate (Hy2) and the solvate (SC and PBE-TS – simulated from the single crystal structure and optimized solvate structure, respectively).

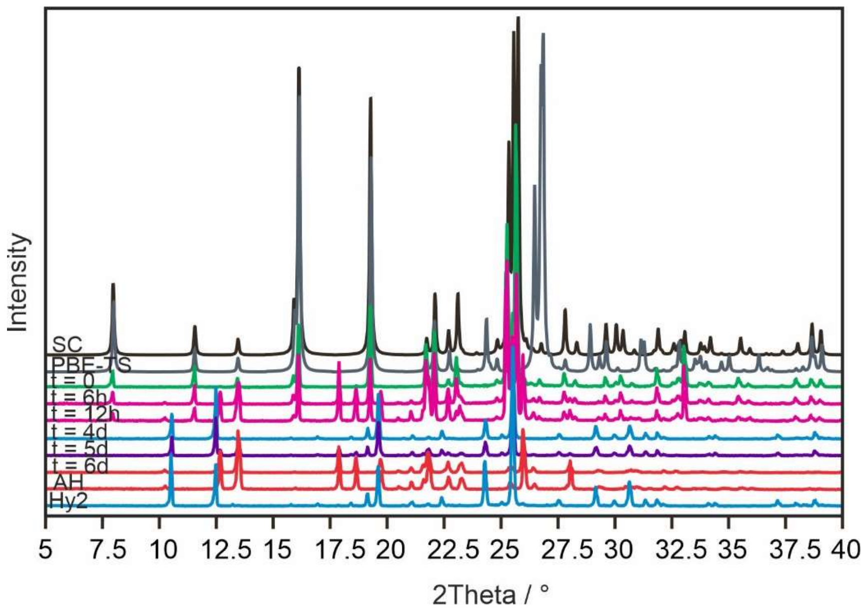

Figure S40. PXRD measurements monitoring the desolvation process of **S-C2₄₄** at ambient conditions (t = x hours). Reference patterns are provided for the anhydrate (AH), hydrate (Hy2) and the solvate (SC and PBE-TS – simulated from the single crystal structure and optimized solvate structure, respectively).

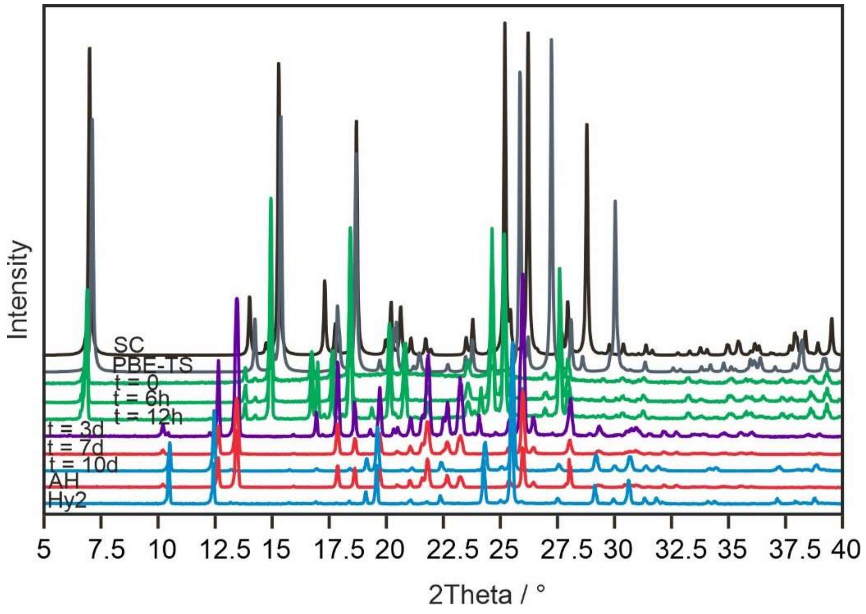

Figure S41. PXRD measurements monitoring the desolvation process of **S-C3₄₄** at ambient conditions (t = x hours). Reference patterns are provided for the anhydrate (AH), hydrate (Hy2) and the solvate (SC and PBE-TS – simulated from the single crystal structure and optimized solvate structure, respectively).

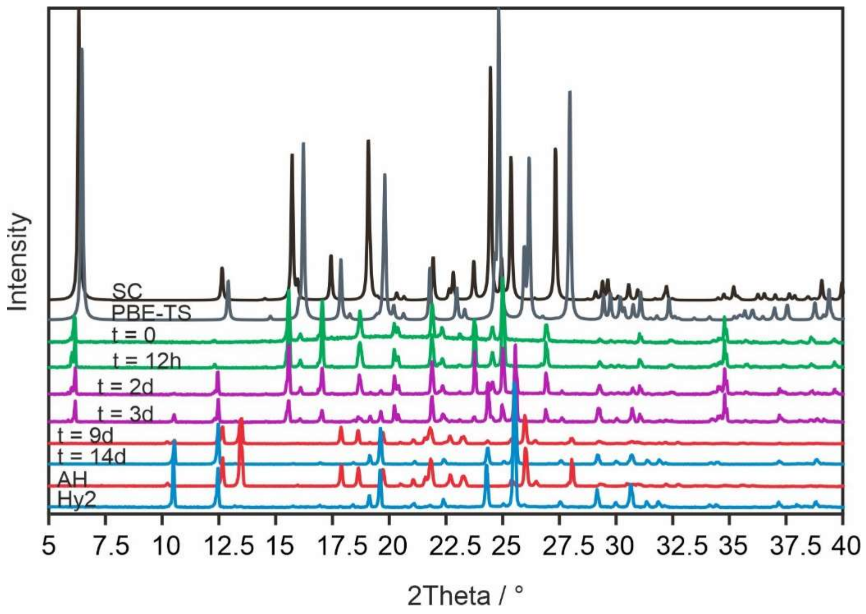

Figure S42. PXRD measurements monitoring the desolvation process of **S-C4$_{44}$** at ambient conditions (t = x hours). Reference patterns are provided for the anhydrate (AH), hydrate (Hy2) and the solvate (SC and PBE-TS – simulated from the single crystal structure and optimized solvate structure, respectively).

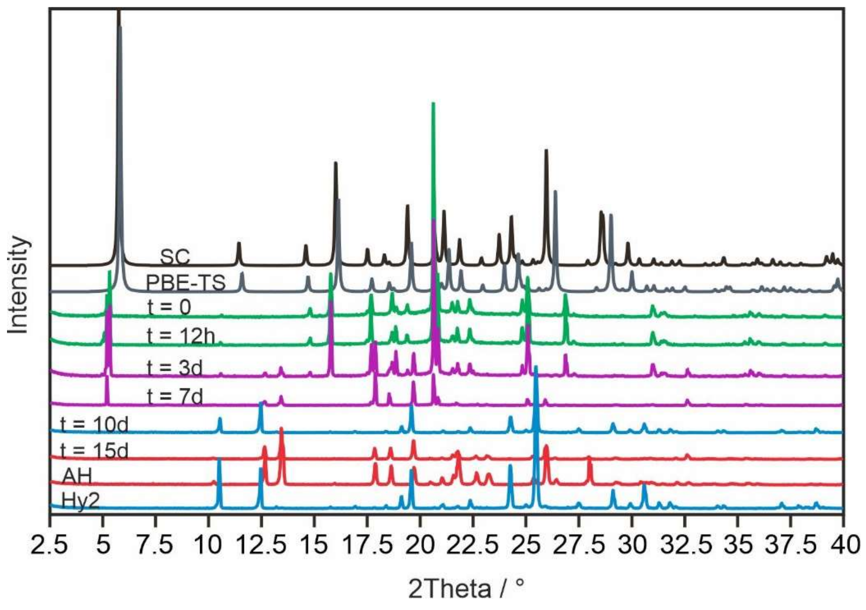

Figure S43. PXRD measurements monitoring the desolvation process of **S-C5$_{44}$** at ambient conditions (t = x hours). Reference patterns are provided for the anhydrate (AH), hydrate (Hy2) and the solvate (SC and PBE-TS – simulated from the single crystal structure and optimized solvate structure, respectively).

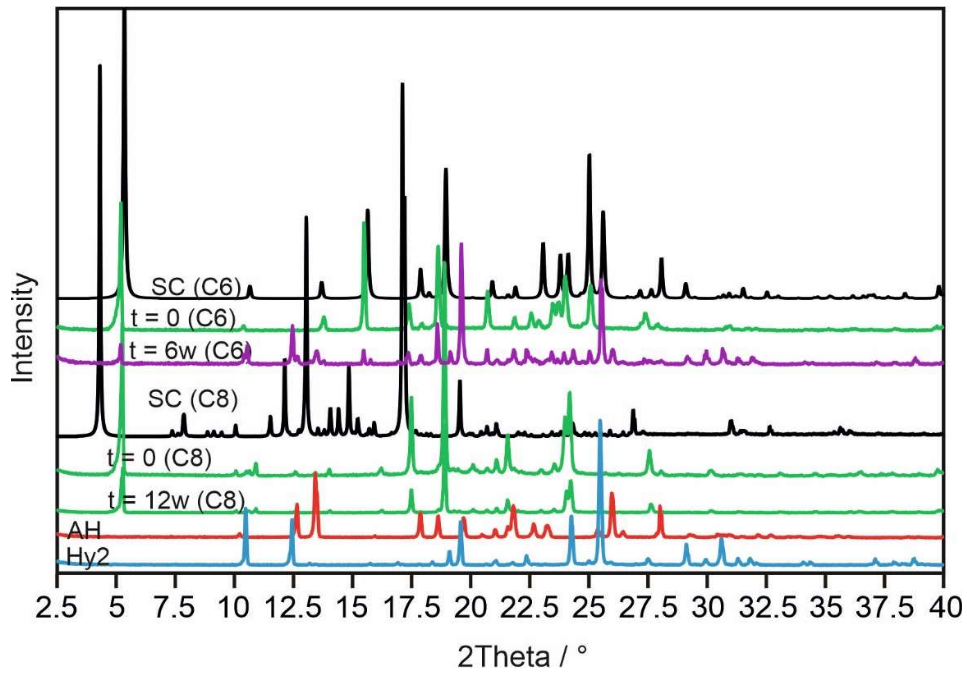

Figure S44. PXRD measurements monitoring the desolvation process of **S-C6₄₄** and **S-C8₄₄** at ambient conditions (t = x hours). Reference patterns are provided for the anhydrate (AH), hydrate (Hy2) and the solvate (SC – simulated from the single crystal structures).

References

(1) Frisch, M. J.; Trucks, G. W.; Schlegel, H. B.; Scuseria, G. E.; Robb, J. M. A.; Cheeseman, R.; Scalmani, G.; Barone, V.; Mennucci, B.; Petersson, G. A.; Nakatsuji, H.; Caricato, M.; Li, X.; Hratchian, H. P.; Izmaylov, A. F.; Bloino, J.; Zheng, G.; Sonnenberg, J. L.; Hada, M.; Ehara, M.; Toyota, K.; Fukuda, R.; Hasegawa, J.; Ishida, M.; Nakajima, T.; Honda, Y.; Kitao, O.; Nakai, H.; Vreven, T.; Montgomery, J. A.; Peralta, J. E.; Ogliaro, F.; Bearpark, M.; Heyd, J. J.; Brothers, E.; Kudin, K. N.; Staroverov, V. N.; Kobayashi, R.; Normand, J.; Raghavachari, K.; Rendell, A.; Burant, J. C.; Iyengar, S. S.; Tomasi, J.; Cossi, M.; Rega, N.; Millam, J. M.; Klene, M.; Knox, J. E.; Cross, J. B.; Bakken, V.; Adamo, C.; Jaramillo, J.; Gomperts, R.; Stratmann, R. E.; Yazyev, O.; Austin, A. J.; Cammi, R.; Pomelli, C.; Ochterski, J. W.; Martin, R. L.; Morokuma, K.; Zakrzewski, V. G.; Voth, G. A.; Salvador, P.; Dannenberg, J. J.; Dapprich, S.; Daniels, A. D.; Farkas, O.; Foresman, J. B.; Ortiz, J. V.; Cioslowski, J.; Fox, D. J. *Gaussian 09*, Gaussian Inc.: Wallingford CT, 2009.

(2) Chisholm, J. A.; Motherwell, S., COMPACK: a program for identifying crystal structure similarity using distances. *Journal of Applied Crystallography* **2005,** 38, 228-231.

(3) Kuhn, F. E.; Groarke, M.; Bencze, E.; Herdtweck, E.; Prazeres, A.; Santos, A. M.; Calhorda, M. J.; Romao, C. C.; Goncalves, I. S.; Lopes, A. D.; Pillinger, M., Octahedral bipyridine and bipyrimidine dioxomolybdenum(VI) complexes: Characterization, application in catalytic epoxidation, and density functional mechanistic study. *Chemistry - A European Journal* **2002,** 8, (10), 2370-2383.

(4) Kraft, S.; Hanuschek, E.; Beckhaus, R.; Haase, D.; Saak, W., Titanium-Based Molecular Squares and Rectangles: Syntheses by Self-Assembly Reactions of Titanocene Fragments and Aromatic N-Heterocycles. *Chemistry - A European Journal* **2005,** 11, (3), 969-978.

(5) Ikemoto, K.; Yang, S.; Naito, H.; Kotani, M.; Sato, S.; Isobe, H., A nitrogen-doped nanotube molecule with atom vacancy defects. *Nature Communications* **2020,** 11, (1), 1807.

(6) Ilyukhin, A., CSD Communication. **2011**.

(7) Ye, L., 4,4'-Bipyridine acetic acid disolvate. *Acta Crystallographica, Section E: Structure Reports Online* **2008,** 64, (1), o46, o46/1-o46/7.

(8) Spek, A. L. *PLATON, A Multipurpose Crystallographic Tool*, Utrecht University: Utrecht, The Netherlands, 2003.
